# Global impact of somatic structural variation on the DNA methylome of human cancers

**DOI:** 10.1186/s13059-019-1818-9

**Published:** 2019-10-15

**Authors:** Yiqun Zhang, Lixing Yang, Melanie Kucherlapati, Angela Hadjipanayis, Angeliki Pantazi, Christopher A. Bristow, Eunjung Alice Lee, Harshad S. Mahadeshwar, Jiabin Tang, Jianhua Zhang, Sahil Seth, Semin Lee, Xiaojia Ren, Xingzhi Song, Huandong Sun, Jonathan Seidman, Lovelace J. Luquette, Ruibin Xi, Lynda Chin, Alexei Protopopov, Peter J. Park, Raju Kucherlapati, Chad J. Creighton

**Affiliations:** 1 Dan L. Duncan Comprehensive Cancer Center, Baylor College of Medicine, Houston, TX 77030 USA; 2 Ben May Department for Cancer Research and Department of Human Genetics, The University of Chicago, Chicago, IL 60637 USA; 3 Department of Genetics, Harvard Medical School, Boston, MA 02115 USA; 4 Division of Genetics, Brigham and Women’s Hospital, Boston, MA 02115 USA; 5 Department of Chemistry and Biochemistry, University of Lethbridge, Lethbridge, AB T1K 3M4 Canada; 6 Department of Genomic Medicine, Institute for Applied Cancer Science, The University of Texas MD Anderson Cancer Center, Houston, TX 77030 USA; 7 Division of Genetics and Genomics, Boston Children’s Hospital, Harvard Medical School, Boston, MA 02115 USA; 8 Center for Biomedical Informatics, Harvard Medical School, Boston, MA 02115 USA; 9 The Eli and Edythe L. Broad Institute of Massachusetts Institute Of Technology and Harvard University, Cambridge, MA 02142 USA; 10 Sanofi US, Cambridge, MA 02139 USA; 11 Department of Bioinformatics and Computational Biology, The University of Texas MD Anderson Cancer Center, Houston, TX 77030 USA; 12 Department of Medicine, Baylor College of Medicine, Houston, TX 77030 USA; 13 Human Genome Sequencing Center, Baylor College of Medicine, Houston, TX 77030 USA

## Abstract

**Background:**

Genomic rearrangements exert a heavy influence on the molecular landscape of cancer. New analytical approaches integrating somatic structural variants (SSVs) with altered gene features represent a framework by which we can assign global significance to a core set of genes, analogous to established methods that identify genes non-randomly targeted by somatic mutation or copy number alteration. While recent studies have defined broad patterns of association involving gene transcription and nearby SSV breakpoints, global alterations in DNA methylation in the context of SSVs remain largely unexplored.

**Results:**

By data integration of whole genome sequencing, RNA sequencing, and DNA methylation arrays from more than 1400 human cancers, we identify hundreds of genes and associated CpG islands (CGIs) for which the nearby presence of a somatic structural variant (SSV) breakpoint is recurrently associated with altered expression or DNA methylation, respectively, independently of copy number alterations. CGIs with SSV-associated increased methylation are predominantly promoter-associated, while CGIs with SSV-associated decreased methylation are enriched for gene body CGIs. Rearrangement of genomic regions normally having higher or lower methylation is often involved in SSV-associated CGI methylation alterations. Across cancers, the overall structural variation burden is associated with a global decrease in methylation, increased expression in methyltransferase genes and DNA damage response genes, and decreased immune cell infiltration.

**Conclusion:**

Genomic rearrangement appears to have a major role in shaping the cancer DNA methylome, to be considered alongside commonly accepted mechanisms including histone modifications and disruption of DNA methyltransferases.

## Introduction

The cancer genome is characterized by widespread genomic rearrangement in addition to point mutations. Somatic structural variations (SSVs) are rearrangements of large DNA segments, which may accompany DNA copy number alterations (CNAs) [1]. Different types of SSVs include deletions, insertions, inversions, tandem duplications, translocations, and more complex rearrangements [2]. In contrast to whole exome sequencing, which focuses on the ~ 1% of the human genome that encodes protein, whole genome sequencing (WGS) may be used to identify SSVs resulting from rearrangements within the cancer genome, each SSV being identified as two distinct genomic coordinates being joined together at a breakpoint junction. Recently, large-scale initiatives including The Cancer Genome Atlas (TCGA) and the Pan-Cancer Analysis of Whole Genomes (PCAWG) have systematically analyzed WGS datasets to identify SSVs across over 3000 cancer cases in total [2–4]. These TCGA and PCAWG datasets both include RNA-seq data uniformly processed and harmonized across the various cancer types, allowing for data integration approaches of gene expression with SSVs. SSVs may impact expression of nearby genes in a number of ways, including forming fusion transcripts or disrupting or repositioning *cis*-regulatory elements near genes. In two separate studies of TCGA and PCAWG data, respectively [3, 5], we developed a systematic analytical approach to integrate SSV breakpoints with the expression of nearby genes, whereby we cataloged hundreds of genes appearing deregulated by rearrangement-mediated *cis*-regulatory alterations.

In addition to genetic mutations and genomic rearrangements, epigenetic alterations, including DNA methylation, play a major role in the development and progression of cancer. CpG islands (CGIs) are short interspersed DNA sequences that deviate significantly from the average genomic pattern by being GC-rich, CpG-rich, and predominantly nonmethylated, with ~ 70% of annotated gene promoters being associated with a CGI [6]. Classical DNA methylation occurs at the 5 position of the pyrimidine ring of the cytosine residues within CpG sites to form 5-methylcytosines, where the presence of multiple methylated CpG sites in CGIs of promoters causes stable silencing of genes [7]. Aberrant DNA methylation in cancer can lead to transcriptional silencing of tumor suppressor genes or a loss of regulation of genes that promote tumorigenesis. Human cancers are characterized by widespread and pervasive changes in the patterns of DNA methylation, which may be attributable to several different mechanisms [8]. It is understood that DNA repair of double-stranded breaks—which would be involved in genomic rearrangements—can lead to altered CpG methylation at the repair site, with corresponding changes in expression of the genes being associated with the repaired region [9–11]. Although previous studies have examined the relationship between CNAs and DNA methylation in cancer [12], to date, there has been no global survey to identify CGIs with altered methylation associated with nearby SSV breakpoints, independently of any associated CNA.

In this present study, taking advantages of the unique resources and opportunities offered by TCGA and PCAWG—which include cancer profiles of DNA methylation for nearly 1500 cases with corresponding SSV data—we set out here to survey genes impacted by SSVs at the levels of either mRNA expression or DNA methylation. By bringing together all available data, a much larger sample set was available for study over that of previous studies, allowing us to further refine the catalog of genes consistently altered in association with nearby SSV breakpoints. Based on previous observations, here we modified our analytical approaches to identify gene alterations by breakpoints occurring across a larger genomic region as well as within specific cancer types. In this present study, we applied approaches that we had originally developed for mRNA data to DNA methylation data, allowing us to identify CGIs and associated genes with methylation being consistently altered by nearby breakpoints. Finally, we identified pan-cancer molecular signatures and involved pathways associated with the overall burden of structural variation across cases—involving both mRNA and DNA methylation.

## Results

### A compendium of SSVs and gene expression across 2334 cases

Our study brought together all available WGS and RNA-seq data for 2334 cases, of which 1482 cases had DNA methylation array data (450K platform) being uniformly generated and processed as part of TCGA consortium. RNA-seq data were previously processed as part of efforts by PCAWG consortium or by TCGA consortium or both, with batch correction being carried out here to harmonize the two sets of data into one unified set of 2334 sample profiles (Additional file 1: Figure S1A). Gene-level CNA values by WGS or SNP array were similarly harmonized together (Additional file 1: Figure S1B). WGS data were generated with either low depth of coverage (low pass, ~ 6–8×) or high coverage (high pass, ~ 30–60×), with both types of WGS being utilized effectively to identify SSVs in previous studies [3, 5]. We compiled SSV calls for 1232 cases from PCAWG [4], for 1207 low-pass WGS cases from our recent study [3], and for 764 high-pass WGS cases from TCGA [2] (Additional files 2 and 3). Cases sequenced with high coverage will have more SSVs detected on average and fewer false negatives [3]. Of the 2334 unique cases, 1033 had only low-pass WGS data available. Data integration (e.g., between SSVs and RNA-seq or between SSVs and DNA methylation arrays) was a key aspect of our study in identifying gene features with significance levels (whether by statistical modeling or permutation testing) rising above any noise inherent in one data platform, with statistical corrections being considered as warranted for technical covariates such as sequencing coverage or tumor sample purities.

**Fig. 1:**
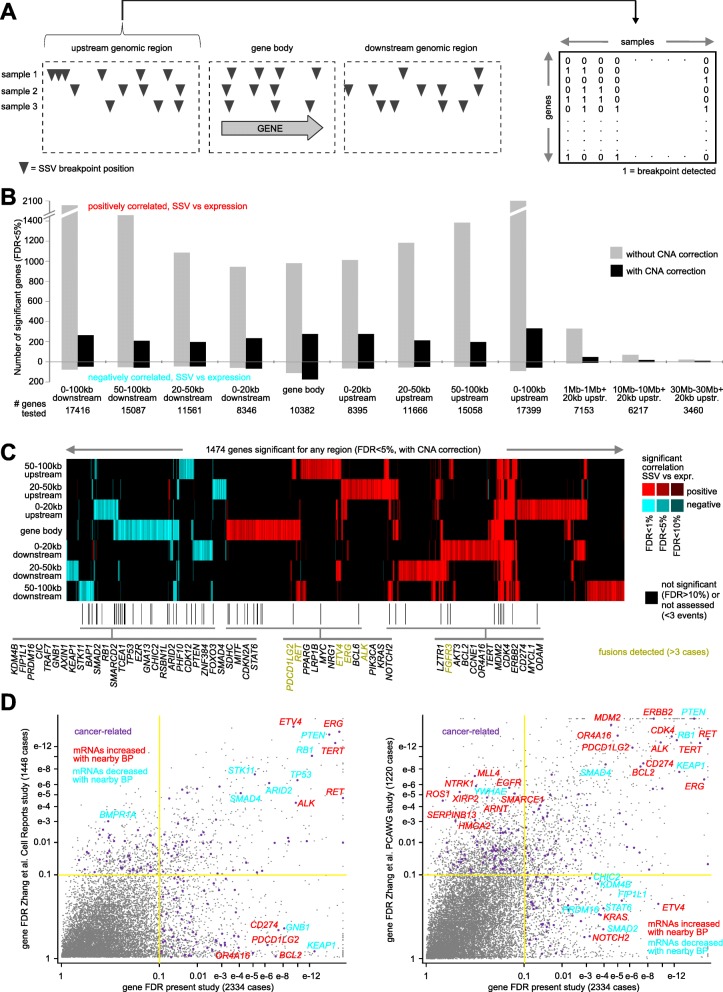
Genes with altered expression associated with nearby SSV breakpoint by the genomic region window method. **a** Schematic of the method. For each of several specified genomic region windows in relation to genes (upstream, downstream, or within the gene body), an SSV breakpoint matrix annotates for each sample the presence or absence of at least one SSV breakpoint within the given region. Across samples, the association between expression and SSV breakpoint pattern for each gene is then assessed. **b** For each of the indicated genomic region windows examined, numbers of significant genes (FDR < 5%), showing correlation between expression and associated SSV event (correcting for sample cancer type), across 2334 cases with WGS and expression data. Numbers above and below zero point of the *Y*-axis denote positively and negatively correlated genes, respectively. Linear regression models also evaluated significant associations when correcting for both cancer type and gene-level CNA. Genes tested for the given region had at least three cases with SSV breakpoint. **c** Heat map of significance patterns for genes from **b** (from the model correcting for both cancer type and CNA). Red, significant positive correlation; blue, significant negative correlation; black, not significant (*p* > 0.05) or not assessed (less than 3 cases with SSV breakpoint events for given gene in the given genomic region). Genes listed are cancer-related [14–16] and with FDR < 1%. **d** Significance of genes in the present study (2334 cases, best FDR from the following regions: 0–20 kb upstream, 20–50 kb upstream, 50–100 kb upstream, 0–20 kb downstream, 20–50 kb downstream, 50–100 kb downstream, or within the gene body), as compared to their significance in both the previous study utilizing low-pass WGS [3], left, and the previous study of PCAWG high-pass WGS [5]. The *X*-axis indicates the best FDR for the present study, and the *Y*-axis indicates the best FDR for the corresponding previous study. Genes in the lower right quadrant reached significance only in the present study. Cancer-related, according to refs [14–16]. See also Additional file 1: Figure S1 and Additional files 2, 3, and 4

Using a previously described integration approach between SSVs and gene expression [3, 13], we assessed gene-level associations between expression and nearby SSV breakpoints within several specified genomic region windows in relation to genes (upstream, downstream, or within the gene body, Fig. 1a). For each of the genomic regions relative to genes considered (e.g., within the gene, 0–20 kb upstream, 20–50 kb upstream, 50–100 kb upstream, 0–20 kb downstream, 20–50 kb downstream, and 50–100 kb downstream), we found widespread associations between SSV event and expression across the 2334 cases and 17,798 genes as expected, after correcting for expression patterns associated with tumor type or CNA (Fig. 1b, c and Additional file 4), indicative of SSV-mediated gene regulatory disruption. For each of the significant gene sets for a given genomic region window (using false discovery rate, or FDR, of < 5% [17]), many more genes were positively correlated with SSV event (i.e., expression was higher when SSV breakpoint was present) than were negatively correlated, though notably a substantial number of genes—including tumor suppressors such as *PTEN*, *STK11*, *TP53*, and *RB1*—were negatively correlated with SSV breakpoints occurring within the gene body, indicative of direct disruption of gene coding regions. In a few cases, within-gene SSV breakpoints associated with increased expression represented gene fusions, e.g., involving *ERG*, *ALK*, or *RET* (Fig. 1c and refs [3, 18]). Without statistical corrections for CNA, we found even larger numbers of genes with SSVs associated with increased expression (Fig. 1b), in line with previous observations of SSV breakpoints being strongly associated with copy gain [3]. The numbers of statistically significant genes were much lower when considering genomic region windows very far away from the gene, e.g., greater than 1 Mb.

The significances of association between expression and nearby SSV breakpoint, as made for all genes in the present study, were compared to that of both the previous study utilizing low-pass WGS [3] and the previous study of PCAWG high-pass WGS [5], involving 1448 and 1220 cases, respectively. Overall, we observed high concordances, between the results from the present study utilizing all 2334 cases and previous results from studies utilizing different subsets of the 2334 cases (Fig. 1d). Genes with known associations with cancer [14–16] that were significantly positively correlated with nearby SSV breakpoints in each of the three separate studies (FDR < 10% for any one of the following regions: 0–20 kb upstream, 20–50 kb upstream, 50–100 kb upstream, 0–20 kb downstream, 20–50 kb downstream, 50–100 kb downstream, or within the gene body, with corrections for cancer type and CNA) included *AKT3*, *ALK*, *BCL9*, *CCND3*, *CDK4*, *ERBB2*, *ERG*, *IRF4*, *LRP1B*, *LZTR1*, *MDM2*, *MYC*, *PPARG*, *RET*, *TERT*, and *TP63*; genes significantly negatively correlated included *ARID1B*, *ARID2*, *GRLF1*, *PHF10*, *PTEN*, *RB1*, *SMAD4*, *STK11*, *TP53*, and *ZNF384*. Here we found many genes that were not significant in one of the previous studies to be significant in the analysis of the combined datasets. This could be attributable in part to greater statistical power represented by the additional cases in the larger dataset, as well as to differences in the representation of cancer types between different studies. For example, SSVs associated with the upregulation of *CD274* and *PDCD1LG2* involved ~ 1% of non-amplified cases in the PCAWG cohort of 1220, but these significant patterns involved lymphomas and other cancer cases that were not represented in the other previous study of 1448 cases, and so these genes were not significant in that study.

### An analytical approach to identify gene features consistently altered by nearby SSV breakpoints

**Fig. 2:**
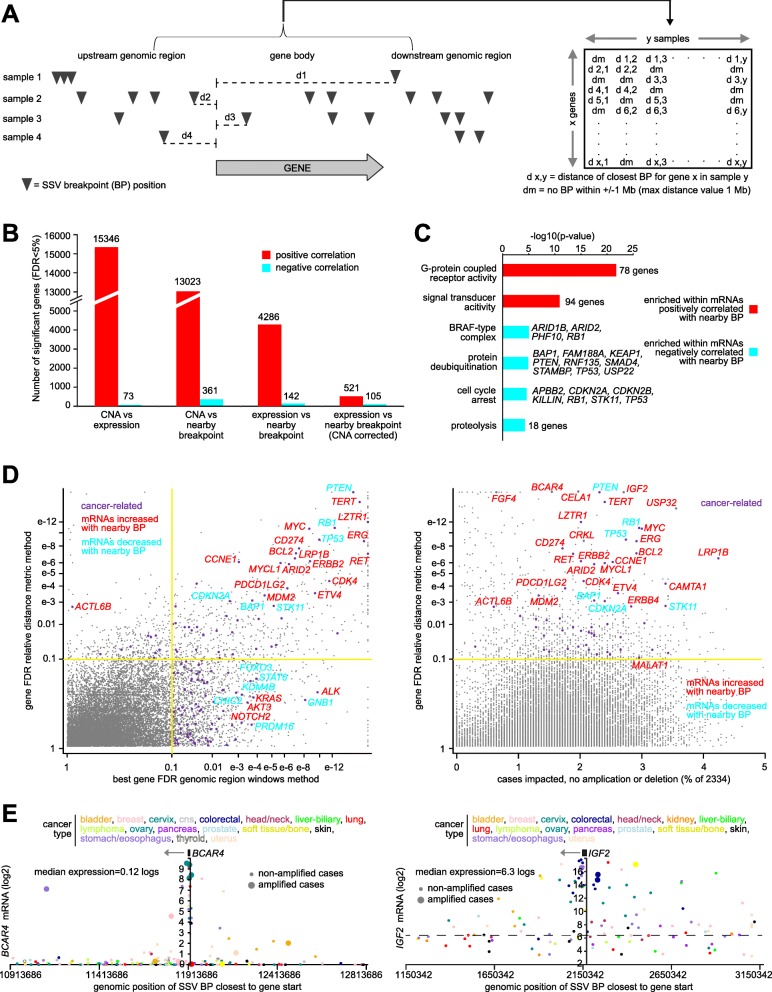
Genes with altered expression associated with nearby SSV breakpoint by distance metric method. **a** Schematic of the method. For each sample, the relative distances of the SSV breakpoint (BP) closest to the start of each gene are tabulated, with a gene X sample relative distance matrix being assembled. Across samples, the association between expression and relative SSV breakpoint distance for each gene (with a maximum distance of 1 Mb) is then assessed. **b** Numbers of significant genes (FDR < 5%, linear model correcting for sample cancer type), across 2334 cases with WGS and expression data, for each of the indicated analyses: (1) gene-level CNA versus expression, (2) CNA versus relative distance to closest SSV breakpoint, (3) expression versus SSV breakpoint distance, and (4) expression versus SSV breakpoint distance with correction for gene-level CNA. **c** Significantly enriched Gene Ontology (GO) terms for genes correlated (FDR < 5% by distance metric method, with corrections for cancer type and CNA) with occurrence of SSV breakpoint in proximity to the gene (for any region considered). *p* values by one-sided Fisher’s exact test. **d** Significance of genes in the present study by distance metric method, as plotted (*Y*-axis) versus significance by genomic region windows method (left, from Fig. 1d, based on 2334 cases, best FDR from the following regions: 0–20 kb upstream, 20–50 kb upstream, 50–100 kb upstream, 0–20 kb downstream, 20–50 kb downstream, 50–100 kb downstream, or within the gene body), and versus the percent of cases impacted (expression > 0.4SD from sample median) by nearby SSV breakpoint (within 1 Mb) without associated amplification or deletion event (defined as log2 tumor/normal copy ratio > 1 or < 1, respectively). Cancer-related, according to refs [14–16]. **e** As examples of significant genes, gene expression levels of *BCAR4* (left) and of *IGF2* (right), corresponding to SSVs located in the genomic region 1 Mb downstream to 1 Mb upstream of the gene. Each point represents a single case (closest SSV breakpoint represented for each case). Cases with gene amplification are indicated. See also Additional file 1: Figures S2 and S3 and Additional file 5

In light of previous findings using the above genomic region windows method, we developed an alternative analytical approach to associate the expression of each gene with nearby SSV breakpoints. Given that genomic rearrangements may involve the translocation of enhancers, which may impact genes within a distance of ~ 1 Mb [3, 19], and given that examining relatively small regions on the order of ~ 20 kb may result in breakpoints falling just outside the window that would otherwise contribute to a significant pattern, we computed a “relative distance metric” for each sample and gene (Fig. 2a), which was the relative distance of the SSV breakpoint closest to the gene start site (upstream or downstream). A data matrix of absolute relative distances for all 17,998 genes and 2334 samples was assembled, with a relative distance metric of 1 Mb being applied for any sample with no breakpoints within 1 Mb of the gene. Using this breakpoint pattern matrix, the correlation between expression of each gene and the presence of nearby SSV breakpoints could be assessed, using linear regression models (on log-transformed expression and relative distance data) that would allow for incorporation of any relevant covariates. Among other things, the distance metric method provides a single result for each gene across the samples, representing genes consistently altered across the entire ± 1 Mb region examined, avoiding the issue of multiple testing of several adjacent regions.

By the distance metric method, we found hundreds of genes consistently altered in expression by SSV breakpoints occurring within a region ± 1 Mb of the given gene, after correcting for CNA (Fig. 2b and Additional file 1: Figure S2 and Additional file 5). Most genes surveyed (13,023 out of 17,798) showed a significant increase in gene copy number with nearby SSV breakpoints, but after corrections for CNA and cancer type, 626 genes showed altered expression with nearby breakpoints at a statistical cutoff of FDR < 5%, 521 of these genes being positively correlated with breakpoints and 105 genes being negatively correlated. We found more genes significant by the distance metric method than were significant for any single region examined by genomic region windows method (Fig. 1b). In addition to CNA, other possible covariates considered included tumor purity, tumor ploidy, low-pass versus high-pass WGS, total number of SSV breakpoints detected per sample, and patient age, none of which represented major confounders (Additional file 1: Figure S3A). In addition, permutation testing, whereby we randomly shuffled the SSV events (by shuffling the patient ids) and distance metric method carried out for each gene, again demonstrated far more significant differences over chance expected (Additional file 1: Figure S3B), consistent with the above FDR estimates by Storey and Tibshirani method [17]. Cases involving overexpression of a gene with nearby SSV spanned all cancer types examined (Additional file 1: Figure S3C). Gene fusion events accounted for a small minority of SSV-mediated gene overexpression (Additional file 1: Figure S3C), and cases with low-pass WGS as well as cases with high-pass WGS contributed substantially to the significant gene patterns observed (Additional file 1: Figure S3C). Significant SSV-gene associations involved all SSV classes and sizes (Additional file 1: Figure S3D and S3E). A set of 37 microRNAs also showed significant associations between nearby breakpoint and increased expression (Additional file 1: Figure S3F and S3G and Additional file 5), which is likely due in part to many microRNAs residing within host genes [20].

Significantly enriched gene categories (by Gene Ontology or GO, Fig. 2c and Additional file 5) within the set of 521 genes positively correlated with nearby SSV breakpoints (FDR < 5%, corrections for CNA and cancer type) included G-protein-coupled receptor activity (78 genes, *p* < 1E−20 by one-sided Fisher’s exact test) and signal transducer activity (94 genes, *p* < 1E−10); enriched gene categories within the set of 105 genes negatively correlated included BRAF-type complex (*ARID1B*, *ARID2*, *PHF10*, *RB1*), protein deubiquitination (*BAP1*, *FAM188A*, *KEAP1*, *PTEN*, *RNF135*, *SMAD4*, *STAMBP*, *TP53*, *USP22*), cell cycle arrest (*APBB2*, *CDKN2A*, *CDKN2B*, *KILLIN*, *RB1*, *STK11*, *TP53*), and proteolysis (18 genes). Overall, the results obtained by the distance metric method were consistent with those of genomic region windows method, although a number of cancer-associated genes were significant by the latter method but not the former method (Fig. 2d); while the distance metric method may aid in our obtaining a more focused set of genes impacted by breakpoints occurring across a larger genomic region, there were other genes impacted just within a smaller and very specific region, as uncovered by the genomic region windows method. A number of well-known oncogenes and tumor suppressor genes had expression impacted by SSV breakpoints in a sizable number of cases—on the order of 1 to 4% of the 2334—that did not harbor amplifications or deletions in the given gene (Fig. 2d), with oncogenes including *TERT* [13], *CRKL* [21], *FGF4* [22], *IGF2* [23], and long noncoding RNA (lncRNA) *BCAR4* [24] (Fig. 2e and Additional file 1: Figure S3H). lncRNA *MALAT1* was also positively correlated with SSV breakpoints (Additional file 1: Figure S3H), though likely not representing an oncogene itself but rather a correlate of aggressive cancers [25].

**Fig. 3:**
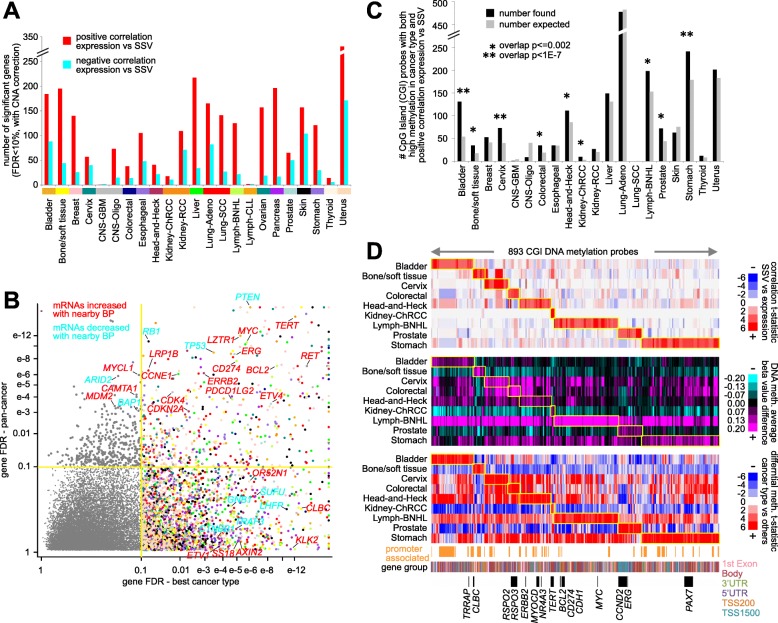
Genes with altered expression associated with nearby SSV breakpoint according to cancer type. **a** For each cancer type, numbers of significant genes showing correlation between expression and nearby SSV breakpoint (FDR < 10% by distance metric method, linear model correcting for CNA). **b** The *X*-axis indicates the FDR in the most significant of the 23 cancer types. The *Y*-axis indicates the FDR when the 2334 cases are analyzed as a combined pan-cancer cohort. Genes in the upper left quadrant reached significance only in the pan-cancer analysis. Genes in the lower right quadrant reached significance only in one or more single-type analyses. Genes in the upper right quadrant were significant in both the pan-cancer set and in individual cancer types. The color of data points represents the most significant cancer type (following **a** color scheme). **c** For each cancer type, numbers of DNA methylation probes (Illumina 450K array platform) targeting CpG islands (CGIs) with high methylation in the given cancer type versus other cancer types (FDR < 0.001, *t* test using logit-transformed data), for which the associated gene also shows a positive correlation between expression and nearby SSV breakpoint for that same cancer type (FDR < 0.1, from **a**). The numbers of CGI probes expected to overlap by chance between the differential methylation results and the expression vs SSV results are also indicated (gray bars), along with any significance of overlap represented by the actual results (asterisks, *p* values by chi-squared test). **d** For 893 CGI DNA methylation probes showing both high cancer type-specific methylation and significant positive correlation between expression and SSV breakpoint for any one of the 20 cancer types surveyed (from **c**), the associated SSV versus expression correlations (from **a**), average DNA methylation by cancer type, and differential methylation in each cancer type versus other cases (t-statistic using logit-transformed values). See also Additional file 1: Figure S4 and Additional file 6

In contrast to the genomic region windows method, the distance metric method allows for inferring SSV-gene associations by individual cancer type, as this method incorporates SSV breakpoint information across a larger genomic region, where SSV events may be sparse. For each of the 23 major cancer types represented in our patient cohort, we assessed the gene-level associations between expression and the presence of nearby breakpoints. For most cancer types surveyed, on the order of hundreds of genes were significantly associated (FDR < 10%, correcting for cancer type and CNA) with SSV breakpoints (Fig. 3a and Additional file 6). As with the pan-cancer analysis, more genes were positively correlated with breakpoints than were negatively correlated. Analogous to findings from genomic surveys for significantly mutated genes [15], a large number of genes found significant in the analysis of individual cancer types did not reach significance when analyzing the combined pan-cancer set of 2334 cases (Fig. 3b). Out of 1280 genes significant for any one individual cancer type (FDR < 10%, correcting for cancer type and CNA), just 41 were significant in pan-cancer analysis (Additional file 1: Figure S4A). The set of SSV-associated genes for each cancer type was distinct from those of the other cancer types (Additional file 1: Figure S4A).

Interestingly, when we compared the cancer type-specific SSV-gene associations with differential DNA methylation patterns (involving *n* = 1482 cases out of the 2334), we observed highly significant overlaps, between the genes with positive correlations between expression and nearby SSV breakpoint for a given cancer type and the genes with high overall DNA methylation being associated with that same cancer type (Fig. 3c and Additional file 1: Figure S4B). We examined DNA methylation array probes for 11,203 CpG islands (CGIs), defining the top CGI probes for each cancer type having high methylation versus the rest of the cancers (FDR < 0.001, *t* test using logit-transformed data). Of the 20 cancer types with methylation data, nine showed a significant overlap (*p* ≤ 0.002, chi-squared test) between genes associated with the top differentially methylated features and genes positively correlated between expression and SSV breakpoints in cancer type-specific analyses. In all, 893 CGI DNA methylation probes—involving 193 genes—were involved in the significant patterns of overlap as described above (Fig. 3d and Additional file 5). One of the involved genes was *TERT*, for which several CGI probes were highly methylated in chromophobe renal cell carcinoma (chRCC) and for which associations between SSV breakpoints and *TERT* expression were significant specifically for that cancer type (Fig. 3d). The *TERT*-associated CGI probes with high methylation were located within the *TERT* gene boundaries and did not include the CGI probe known to represent a repressive regulatory element (probe cg02545192, Additional file 1: Figure S4C). Of note, the discovery of SSV-mediated deregulation of *TERT* in solid tumors was first made in the chRCC cancer type [13], though the associations involving DNA methylation had not previously been made.

### Widespread impact of SSVs on methylation of specific CpG islands (CGIs) across 1482 cases

**Fig. 4:**
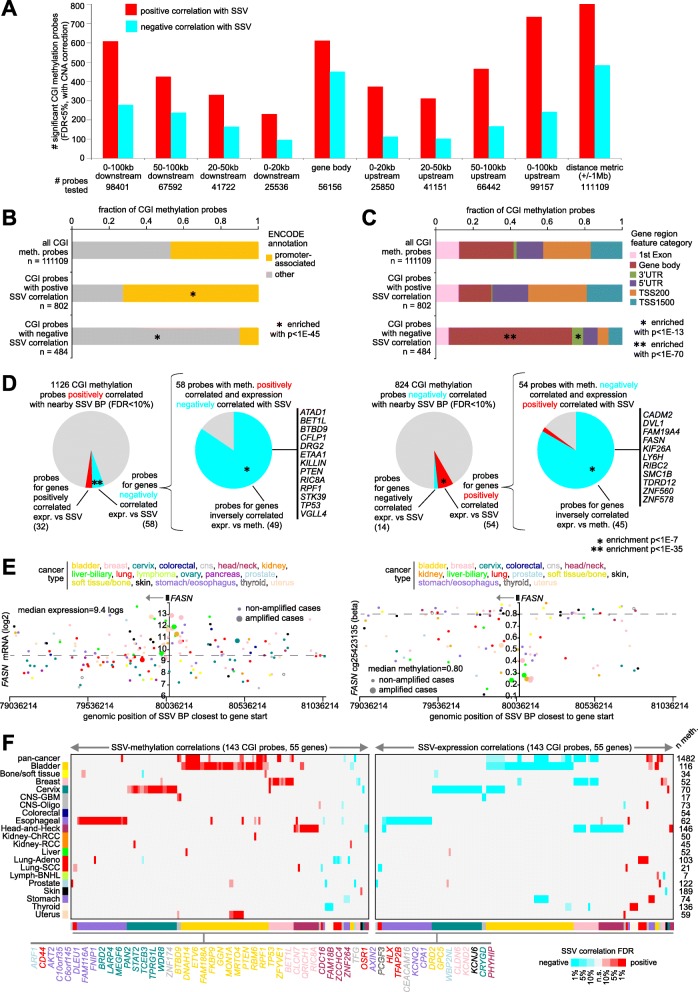
CpG islands (CGIs) with altered DNA methylation associated with nearby SSV breakpoint. **a** For each of the indicated genomic region windows in relation to genes associated with CGIs, numbers of significant CGIs (FDR < 5%, correcting for both cancer type and gene-level CNA), showing correlation between DNA methylation and associated SSV event, across 1482 cases with WGS and DNA methylation data. Results from distance metric method (± 1 Mb) as well as genomic region windows methods are shown. CGI DNA methylation probes tested for the given region had at least three cases with SSV breakpoint. **b** Fraction of promoter-associated CGIs, for the CGIs associated with increased methylation (by distance metric method from **a**), and for the CGIs associated with decreased methylation. *p* values by chi-square test. **c** Breakdown by probe position relative to gene, for the CGIs associated with increased or decreased methylation, respectively. *p* values by chi-square test. **d** Overlap between CGI probes with SSV-associated altered methylation and nearby genes with corresponding SSV-associated altered expression. For expression- and DNA methylation-SSV breakpoint associations inverse to each other for the same genes (e.g., gene expression positively correlated and DNA methylation negatively correlated with nearby breakpoint, using FDR < 10%), the subset of CGI methylation probes for which the associated genes are negatively correlated between methylation and expression (FDR < 10% by Pearson’s correlation on logit- or log-transformed values) across the 1482 cases are indicated. Genes highlighted are represented by multiple CGI probes. *p* value for significance of overlap by chi-squared test. **e** As an example of a significant gene, gene expression levels (left) and DNA methylation levels (right) of *FASN*, corresponding to SSVs located in the genomic region 1 Mb downstream to 1 Mb upstream of the gene. Each point represents a single case (closest SSV breakpoint represented for each case). Cases with gene amplification are indicated. **f** A set of 143 CGI methylation probes significantly associated with nearby SSV breakpoints for one or more individual cancer types (FDR < 10%), for which the expression versus SSV association for the corresponding gene within the same cancer type are significant (FDR < 10%) and in the opposite direction. Left panel represents SSV-methylation associations; right panel represents SSV-expression associations. FDR values by distance metric method, correcting for CNA. Gene name coloring corresponds to associated cancer type. See also Additional file 1: Figures S5 and S6 and Additional file 7

While previous studies have examined the global influence of SSVs on the expression of individual genes, an analogous survey of associations between SSVs and DNA methylation patterns remained to be carried out. The gene by sample breakpoint matrices as constructed above for analysis of gene expression (Figs. 1a and 2a) were joined to the DNA methylation data matrix of 1482 cases, in terms of the genes associated with CGIs. The correlation between methylation of each CGI and the presence of an SSV breakpoint in relation to the CGI-associated gene was assessed using linear regression models, with the inclusion of relevant covariates. We examined 111,203 CGI DNA methylation probes, involving 13,043 associated genes. After correcting for any associations that would be attributable to CNA [12], we found hundreds of CGI probes consistently altered in methylation by nearby SSV breakpoints, whether by examining smaller regions by genomic region windows method or by surveying the ± 1 Mb region surrounding each gene by the distance metric method (Fig. 4a and Additional file 7). More CGI features were positively correlated (i.e., showed increased methylation) with SSV breakpoints than were negatively correlated. By the distance metric method, 1286 significant CGI probes (FDR < 5%, Fig. 4a and Additional file 1: Figure S5) included 802 probes positively correlated with nearby breakpoint and 484 probes negatively correlated. By and large, a number of possible covariates considered did not represent major confounders of the methylation-SSV associations (Additional file 1: Figure S6A), although a number of CGI probes negatively correlated with nearby SSV appeared influenced in part by the total number of SSV breakpoints detected per sample and by sample-wide DNA methylation (Additional file 1: Figure S6A), which associations were further explored below. Along with statistical modeling, permutation testing by random shuffling of the SSV events also demonstrated far more significant differences over chance expected (Additional file 1: Figure S6B).

Strikingly, CGI probes with SSV-associated increased methylation were predominantly promoter-associated, while CGI probes with SSV-associated decreased methylation were enriched for gene body CGIs (Fig. 4b, c; Additional file 1: Figure S6C). Of the 802 probes positively correlated (FDR < 5%) with nearby breakpoint, 581 (72%) were promoter-associated according to ENCODE annotation of the 450K platform (Additional file 7), where ~ 377 probes (47%) would have been expected by chance (*p* < 1E−45, chi-square test). Of the 484 probes negatively correlated with nearby breakpoint, these were anti-enriched for promoter-associated probes (Fig. 4b), but included 321 probes located within the gene body (between the ATG and stop codon, by 450K platform annotation), as compared to ~ 142 expected by chance (*p* < 1E−70, chi-square test). Interestingly, while promoter methylation is known to silence genes, gene body methylation is often positively correlated with gene expression [26], though our significant CGIs would represent only a fraction of the total surveyed. Significant SSV-CGI associations involved all SSV classes and sizes (Additional file 1: Figure S6D and S6E).

We examined the overlap between CGI probes with SSV-associated altered methylation and the related genes with corresponding SSV-associated altered expression (Fig. 4d), taking particular note of inverse correlations between methylation and expression. Using FDR cutoffs of < 10% (distance metric method, with cancer type and CNA corrections), 1126 CGI methylation probes were positively correlated with nearby SSV breakpoints, of which 58 probes involved genes negatively correlated between expression and SSV breakpoints, a highly significant overlap (*p* < 1E−35, chi-squared test), with 49 of the 58 probes also showing inverse correlation between expression and methylation across the 1482 cases (FDR < 10%, Pearson’s correlation using log- or logit-transformed values, respectively, with corrections for cancer type and CNA), which genes included *TP53* and *PTEN* (Fig. 4d and Additional file 1: Figure S6F). Out of 824 CGI methylation probes negatively correlated with nearby SSV breakpoints, 54 involved genes positively correlated between expression and SSV breakpoints (*p* < 1E−7, chi-squared test), which genes included *DVL1* and *FASN* (Fig. 4e and Additional file 1: Figure S6F). Previous findings made elsewhere [5] of other types of DNA methylation patterns associated with genes positively correlated with nearby SSV breakpoints were also observable in the present study, namely an enrichment for genes positively correlated between expression and methylation (Additional file 1: Figure S6G), as well as an association of methylation at the *TERT*-associated cg02545192 site (which involves a repressor element) with increased *TERT* expression (Additional file 1: Figure S6H). For most individual cancer types, on the order of hundreds of CGI probes were significantly associated with altered methylation with nearby SSV breakpoints (Additional file 1: Figure S7A). Out of 16,096 probes significant by methylation analysis for any one individual cancer type (FDR < 10%, Additional file 1: Figure S7B), 143, representing 55 genes, showed an opposite and significant correlation between expression and SSVs for the corresponding gene within the same cancer type (Fig. 4f).

**Fig. 5:**
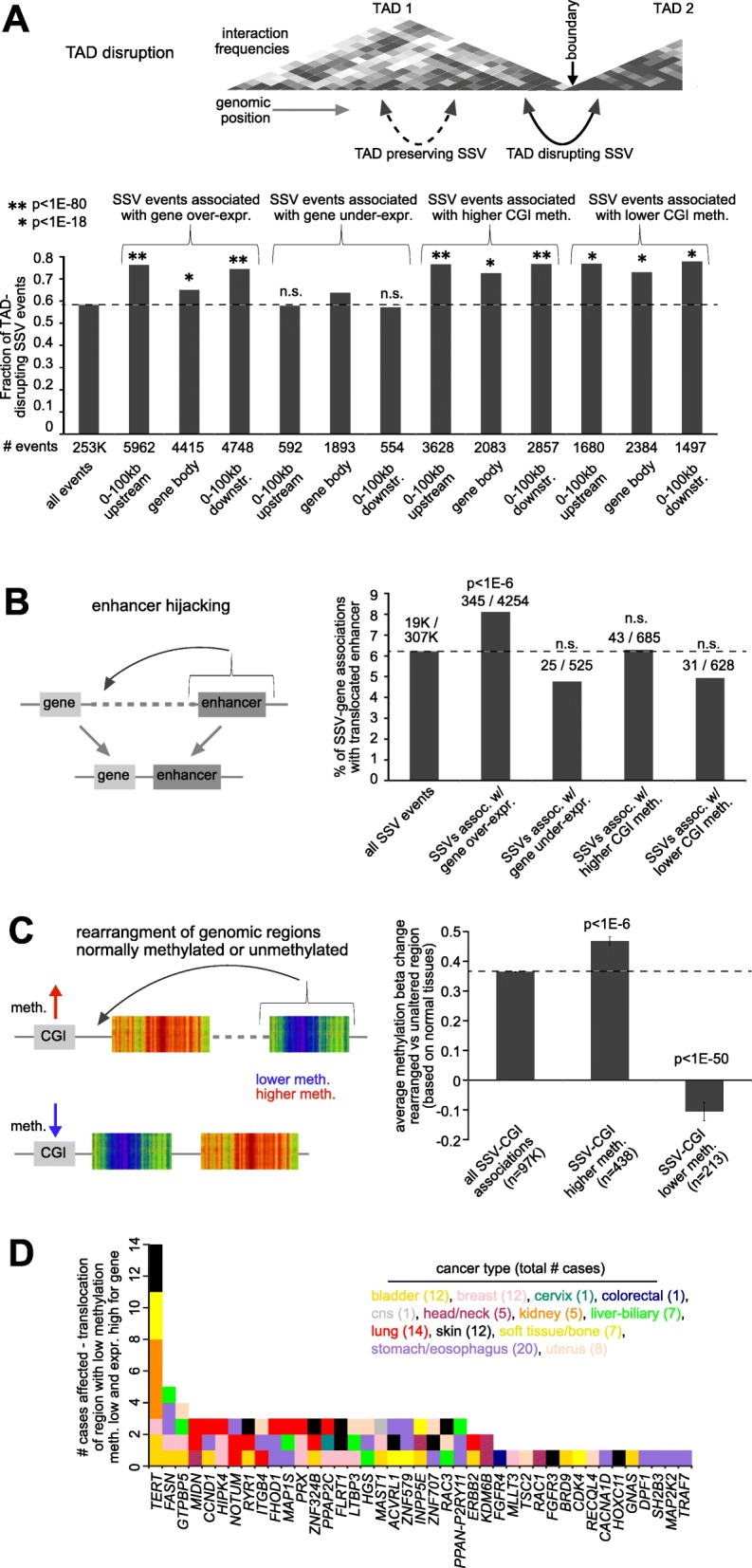
SSVs associated with TAD disruption, enhancer hijacking, and rearrangement of regions with high or low methylation. **a** As compared to all SSVs, fractions of SSVs involving topologically associated domain (TAD) disruption and altered gene expression or DNA methylation (defined as FDR < 5% for the gene or CGI probe within the given region window, with corrections for cancer type and CNA, and expression > 0.4SD or < − 4SD from median for the case harboring the breakpoint). *p* values by chi-squared test. **b** Percentages of SSV breakpoint associations involving the translocation of an active, in vivo-transcribed enhancer [28] within 0.5 Mb of the gene (where the unaltered gene had no enhancer within 1 Mb), as tabulated for the entire set of SSV breakpoint associations occurring 0–500 kb upstream of a gene and with breakpoint mate on the distal side from the gene, as well as for the subsets of SSV breakpoint associations involving altered gene expression or CGI methylation (using FDR < 5% by distance metric and > 0.4SD or < − 4SD, with corrections for cancer type and CNA). *p* values for enrichment as compared to all SSV events by chi-squared test. **c** Using a dataset of DNA methylation of normal tissues (Additional file 1: Figure S7), the average DNA methylation represented by the rearranged region (using window of 50 kb) was compared with that of the CGI nearby the gene on the other side of the SSV breakpoint, with the average difference in methylation beta values computed for all SSV-CGI associations, as well as for the subset of SSV-CGI associations involving higher or lower DNA methylation (defined as for **b**). *p* values by Spearman’s rank correlation. Bars represent standard error. **d** By gene and by cancer type, the number cancer cases involving the rearrangement of a region of low methylation (average methylation beta difference < − 0.1), with corresponding decrease in methylation and increase in expression being observed (< − 4SD and > 0.4SD from median, respectively), involving 41 genes and 105 cases (genes affected in > 2 cases or cancer-associated genes [14–16] being represented here). See also Additional file 1: Figure S7 and Additional files 8, 9, and 10

We explored potential mechanisms involving SSV-mediated alterations in gene expression and in DNA methylation, including disruption of topologically associated domains (TADs) and enhancer hijacking. Using published data on TAD coordinates in human cells [27], we categorized all SSVs in our pan-cancer dataset, by those that were TAD disrupting (i.e., the breakpoints span two different TADs) versus those that were non-disrupting (i.e., both breakpoints fell within the same TAD). As expected [3], for SSVs with breakpoints located in proximity to a gene and associated with its overexpression, a high enrichment (*p* < 1E−18, chi-square test) for TAD-disrupting SSVs was observed (Fig. 5a), though no similar enrichment pattern was observed for SSVs associated with gene underexpression. Interestingly, SSVs associated with higher CGI methylation or with lower CGI methylation were both highly enriched for TAD-disrupting SSVs (Fig. 5a and Additional file 8, *p* < 1E−18, chi-square test). We went on to examine potential enhancer hijacking events involving SSVs, focusing here on a set of active, in vivo-transcribed enhancers as cataloged previously [28]. For all SSV breakpoint associations occurring 0–500 kb upstream of a gene and with breakpoint mate on the distal side from the gene, we tabulated SSV breakpoint associations involving the translocation of an active, in vivo-transcribed enhancer within 0.5 Mb of the gene (assuming no other disruptions involving the region), where the unaltered gene had no enhancer within 1 Mb. In line with previous findings [3], the subset of SSV breakpoint associations involving gene overexpression showed a statistically significant percentage (8.1% versus 6.2%) involving putative enhancer translocation events (Fig. 5b and Additional file 9, *p* < 1E−6, chi-squared test). However, as might be expected, we observed no such enrichment patterns for SSVs associated with gene underexpression or with altered DNA methylation (Fig. 5b).

Interestingly, rearrangement of regions with higher or lower methylation from other parts of the genome was associated with SSV-associated DNA methylation alterations. Using the TCGA dataset of DNA methylation of 16 types of normal adjacent tissues and ~ 450K probes (Additional file 1: Figure S7C), for each SSV breakpoint, the average DNA methylation in normal tissues represented by the rearranged region (using window of 50 kb) was compared with the normal tissue methylation of the CGI nearby the gene on the other side of the breakpoint. This average difference in normal methylation represented by SSV breakpoint was computed for all SSV-CGI associations, as well as for the subset of SSV-CGI associations involving higher or lower DNA methylation for the CGI in the cancer sample (using FDR < 5% for the CGI by distance metric and > 0.4SD or < −4SD from sample median for the case harboring the breakpoint, with corrections for cancer type and CNA). On average, all SSV-CGI associations showed an increase in methylation represented by the rearranged region relative to the CGI on the other side of the breakpoint (average beta difference of + 0.37, Fig. 5c, Additional file 1: Figure S7C, S7D, and S7E), which reflects the fact that regions of the genome outside of CGIs tend to have higher DNA methylation (Additional file 1: Figure S7C). However, when considering the subset of SSV-CGI associations involving higher methylation in the cancer, the increase in methylation involving the rearranged region was even greater (average beta difference of + 0.47, Spearman’s *p* < 1E−6, Fig. 5c), and SSV-CGI associations involving lower methylation showed a highly significant decrease in methylation represented by the rearranged region (average beta difference of − 0.11, Spearman’s *p* < 1E−50, Fig. 5c). The top SSV-CGI associations involving the rearrangement of a region of low methylation (average methylation beta difference < − 0.1), with corresponding decrease in methylation and increase in expression being observed in the cancer case (< − 4SD and > 0.4SD from median, respectively), involved 314 unique cancer cases and 549 unique genes (Additional file 10), including genes such as *TERT* (14 cases) and *FASN* (5 cases, Fig. 5d).

### Widespread molecular alterations associated with the overall burden of structural variation

**Fig. 6:**
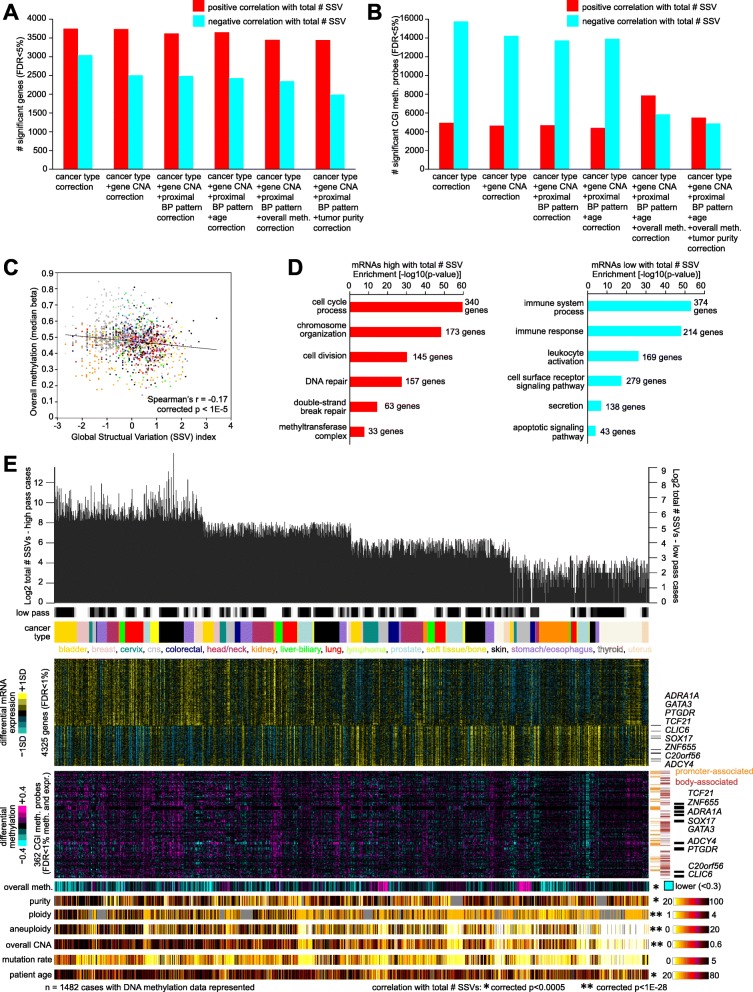
Global alterations in transcription and DNA methylation associated with the overall burden of structural variation across cancers. **a** Numbers of significant genes (FDR < 5%), showing correlation between expression and the total number of SSV events detected across the 2334 cases with RNA-seq data. Linear regression models evaluated significant associations when correcting for specific covariates (in addition to low-pass versus high-pass WGS), as indicated. **b** Numbers of significant CGI probes (FDR < 5%), showing correlation between DNA methylation and the total number of SSV events detected across the 1482 cases with methylation data. Linear regression models evaluated significant associations when correcting for specific covariates (in addition to low-pass versus high-pass WGS), as indicated. **c** Scatter plot of global SSV index (measuring total number of SSV events, correcting for high-pass versus low-pass WGS) versus overall methylation (median beta of all 450K probes within the sample profile). *p* value by linear model correcting for cancer type. **d** Significantly enriched GO terms for genes correlated (FDR < 1%, with corrections for cancer type, CNA, and low-pass versus high-pass WGS) with the total number of SSV events. *p* values by one-sided Fisher’s exact test. **e** Across the 1482 cases with DNA methylation data, with cases ranked high to low by global SSV index quartiles, selected molecular features are represented, including top expression correlates with total number of SSV events (from **d**), CGI probes with DNA methylation high with total number of SSV events (FDR < 1%, correcting for cancer type, low-pass versus high-pass WGS, CNA, proximal BP pattern, age, and overall methylation) and with associated mRNAs low with total number of SSV events (as well as anti-correlation between mRNA and methylation, Pearson’s FDR < 10%), overall methylation (from **c**), tumor purity, tumor ploidy, aneuploidy [29], overall CNA, exome mutation rate, and patient age. Expression and methylation values are normalized or centered within each cancer type. Highlighted genes are represented by multiple CGI probes. *p* values by linear model correcting for both cancer type and low-pass versus high-pass WGS. See also Additional file 1: Figure S8 and Additional file 11

As another line of investigation, we examined gene expression and DNA methylation features that were correlated with the total number of SSV breakpoints detected per sample, independent of where the breakpoints were located in relation to genes. The hypothesis explored here is that cancer cases with a high burden of structural variation may show an altered molecular profile as a result of the extensive DNA damage involved. As low-pass WGS on average would yield more false negative SSV events, low-pass versus high-pass WGS was incorporated as a covariate in the statistical modeling, along with the TCGA or ICGC project to factor in other technical differences between projects. We found thousands of molecular correlates at both the mRNA and DNA methylation levels (FDR < 10% by linear regression model), when factoring the above covariates as well as other potential biological or technical covariates (Fig. 6a, b and Additional file 11). Most covariates considered—which included CNA, proximal BP pattern (from Fig. 2a), patient age, tumor purity, and overall methylation—did not represent major confounders in inferring molecular correlations with the total number of SSV events detected across samples, with the one notable exception being overall methylation (the median beta across all 450K probes in the sample profile) as applied to the DNA methylation analyses, whereby thousands of CGI probes that had been significantly negatively correlated with the total number of SSVs lost significance when overall methylation was factored into the model, indicating that globally lower methylation levels overall could be associated with the total number of SSVs.

In addition to specific mRNA and DNA methylation features, other molecular variables were associated with the total number of SSVs across samples. A slight but significant decrease in overall DNA methylation (median beta of the 450K probes) with increasing numbers of SSV was observed, independent of high-pass or low-pass WGS (Fig. 6c, Additional file 1: Figure S8A-D), Spearman’s *r* = − 0.17, corrected *p* < 1E−5), and accounting for many of the individual CGI probes that were significantly negatively correlated with the total number of SSVs (Fig. 6b). Significantly enriched GO gene categories (Fig. 6d and Additional file 11) within the set of 2661 genes positively correlated with the total number of SSVs per sample (FDR < 1%, with corrections for cancer type, CNA, and low-pass versus high-pass WGS) included cell cycle process (340 genes, *p* < 1E−50 by one-sided Fisher’s exact test), chromosome organization (173 genes, *p* < 1E−45), cell division (145 genes, *p* < 1E−30), DNA repair (157 genes, *p* < 1E−25), double-strand break repair (63 genes, *p* < 1E−14), and methyltransferase complex (33 genes, *p* < 1E−7); enriched gene categories within the set of 1611 genes negatively correlated included immune system process (374 genes, *p* < 1E−50), immune response (214 genes, *p* < 1E−45), leukocyte activation (169 genes, *p* < 1E−25), and apoptotic signaling pathway (43 genes, *p* < 0.0001). Consistent with the observed overexpression of cell cycle genes, a trend of increased number of SSVs detected in tumor samples and overall patient survival was observed (Additional file 1: Figure S8E). Other tumor sample variables associated with the total number of SSVs (*p* < 0.0005, corrected Pearson’s) included tumor sample purity, tumor ploidy, tumor aneuploidy, overall CNA, and patient age (Fig. 6e and Additional file 1: Figure S8F and S8G). Genes associated with both low expression and high DNA methylation with increasing numbers of SSVs included *ADRA1A*, *GATA3*, *TCF21*, and *SOX17* (Fig. 6e and Additional file 1: Figure S8H).

**Fig. 7:**
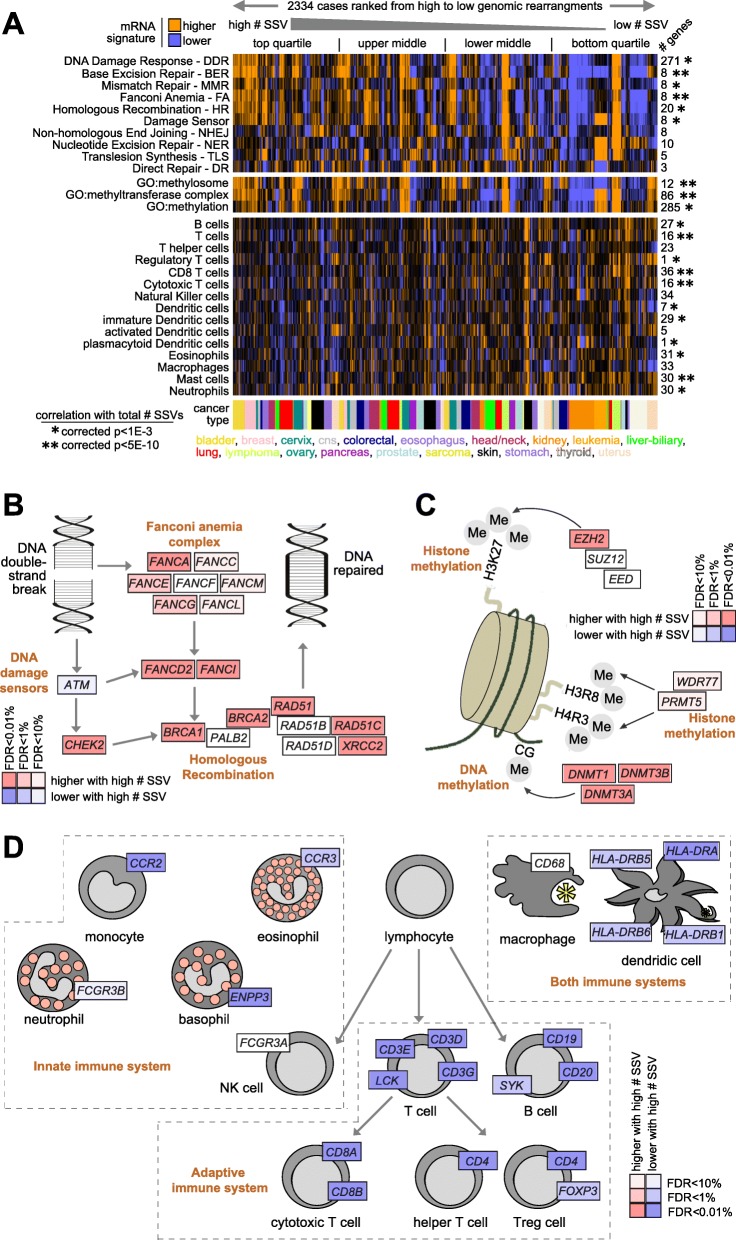
Gene signatures and pathways associated with the overall burden of structural variation across cancers. **a** Across the 2334 cases with both WGS and RNA-seq data, heat maps of gene expression-based signatures scoring for DNA damage response pathways [30], methylation (by Gene Ontology or GO categories), and immune cell infiltrates [31]. Gene signature scores are the average of normalized expression values (for immune signatures, expression values normalized within each cancer type; for other signatures, expression values normalized across sample profiles). *p* values by linear model correcting for both cancer type and low-pass versus high-pass WGS. **b** Diagram of key genes involved with DNA double-strand break repair pathway, with corresponding correlation with the overall structural variation burden (red, significantly higher with increasing number of SSVs). **c** Similar to **b**, with diagram of key genes involved in methylation of DNA and histones. **d** Similar to **b**, with diagram of immune cell types and associated gene markers. FDR values in **b** and **c** (based on the entire set of 17,798 genes profiled) by linear model correcting for both cancer type and low-pass versus high-pass WGS

Based on the above GO term enrichment patterns (Fig. 6d), we examined gene transcription signatures of DNA damage response, of methylation, and of immune cell types across the 2334 cases with expression data (Fig. 7a). Gene signature analyses using a previously curated list of 276 genes encompassing all major DNA repair pathways [30] showed several of these to be elevated in cases with high numbers of SSVs, including signatures of base excision repair, mismatch repair, Fanconi anemia, and homologous recombination (Fig. 7a). The associations involving DNA double-strand break repair pathway and Fanconi anemia, in particular, were also evident when examining key individual genes, including *BRCA1*, *BRCA2*, *FANCD2*, *FANCI*, and *RAD51* (Fig. 7b). Key genes involved in histone methylation and DNA methylation, which appeared significantly increased with increasing numbers of SSV events, included *EZH2*, *WDR77*, *PRMT6*, *DNMT1*, *DMNT3A*, and *DMNT3B* (Fig. 7c). Analysis of gene expression signatures from Bindea et al. [16, 31] suggested that levels of immune cell infiltrates (e.g., B cells, T cells, and dendritic cells) were higher within tumors harboring fewer SSVs, which was also evident from the analysis of canonical immune cell gene markers (Fig. 7a, d). A DNA methylation signature of leucocyte fraction [32] also showed a similar pattern of anti-correlation with increasing total numbers of SSVs (Additional file 1: Figure S8F, Pearson’s corrected *p* < 1E−8), indicating that some of the global DNA methylation patterns as well as the gene expression patterns identified may involve non-cancer cell types.

## Discussion

This study provides a more comprehensive catalog of mRNAs deregulated by nearby somatic genomic rearrangements, both in pan-cancer analyses and in analyses of individual cancer types. Our distance metric approach to integrate SSVs with altered gene features, as introduced in the present study, provides a framework by which we can assign global significance to a core set of genes, analogous to methods such as MutSig [15] or GISTIC [33] that identify genes appearing non-randomly targeted by somatic mutation or CNA, respectively. As observed with significance of mutation patterns across cancers [15], many genes show significance within a specific cancer type but do not appear significant in pan-cancer analyses. In pan-cancer analyses, many known oncogenes (e.g., *BCAR4*, *BCL2*, *CCNE1*, *CD274*, *CDK4*, *CRKL*, *ERBB2*, *FGF4*, *IGF2*, *PDCD1LG2*, *MDM2*, *MYC*, and *TERT*) are overexpressed with SSV breakpoints located near the gene, and many tumor suppressor genes (notably *PTEN*, *STK11*, *TP53*, and *RB1*) are underexpressed with breakpoints located within the gene. As is the case involving CNA patterns, SSV-altered genes may involve passengers as well as drivers, where other sources of information, including global mutation and CNA patterns, may be brought to bear in uncovering novel potential driver genes as uncovered by SSV analysis. Analogous to observations involving point mutation analysis [15], many cancer-associated genes may be altered by SSVs at relatively small frequencies (e.g., 1–3%), and so as more WGS data are brought into the public domain, the greater samples and power offered by such data can lead to additional significant genes being identified by our approaches, both across all cancers as well as within individual cancer types.

This present study has revealed an apparent widespread impact of SSVs on CGI methylation in human cancer, independently of CNA. Previous studies have defined broad patterns of association involving gene transcription and nearby SSV breakpoints based on RNA-seq and WGS analysis, with these patterns collectively involving large groups of genes [3, 5, 34, 35], though none of these studies had surveyed DNA methylation patterns in the context of SSVs. It has been understood that DNA repair of double-stranded breaks can lead to altered CpG methylation at the repair site [9–11], but the findings made here of specific CGIs appearing recurrently and non-randomly altered in association with nearby SSV breakpoints across cancers, independently of any associated CNA, is intriguing and suggestive of a selection process in the disease. A shorter list of genes was found to be associated with SSV breakpoints inversely between DNA methylation and expression, with some notable genes including Wnt pathway-related *DVL1* and metabolism-related *FASN*. Other genes, such as *TERT*, appear altered in expression in some cases due in part to corresponding DNA methylation changes and in other cases due to other mechanisms including enhancer hijacking [19], and so while *TERT*-associated CGIs may not be globally associated with DNA methylation changes across all cases, in a subset of cases, methylation alterations may still play a role in deregulated expression. In evaluating the true impact of DNA methylation alterations for a given gene, the absolute level of DNA methylation changes as well as domain-specific knowledge regarding regulation of the gene should be taken into account.

This study identified a phenomenon involving the rearrangement of genomic regions with higher or lower methylation as a potential contributor to the observed SSV-associated DNA methylation alterations. TADs can confine physical and regulatory interactions between enhancers and their target promoters and if disrupted can result in ectopic gene expression [23], consistent with observations in the present study. TAD disruption in cancer was also associated here with global changes in DNA methylation (both higher and lower), which would be related to the rearrangement of differentially methylated regions, where such rearrangements involving two regions representing dramatically different methylation landscapes are more likely to span TAD boundaries rather than involving short-range rearrangements within a TAD. A number of possible mechanisms would be at work in DNA methylation alterations in cancer, including disruption of genes involved with methylation such as DNA methyltransferases (DNMTs), modifications of histones (e.g., by deregulation of enhancer of zeste drosophila homologue 2, or EZH2) that mark a gene for hypermethylation, and processes involved in DNA repair of double-stranded breaks [11, 36]. The findings made in the present study regarding rearrangement of differentially methylated genomic regions would seem to represent an under-appreciated mechanism involved in shaping the cancer DNA methylome. For some genes, such as *TERT*, higher levels of DNA methylation for some cancer types might represent a barrier to gene overexpression, which SSVs appear to help overcome in many cases of our patient cohort. Multiple mechanisms of SSV-mediated deregulation at both the gene transcription and DNA methylation levels appear to be involved, which may be further elucidated in future studies.

Our study also identified widespread molecular alterations associated with the overall structural variation burden across cancers, which were distinct from that of the overall mutational burden. Overall structural variation burden (i.e., high number of detected SSVs relative to other cases, correcting for differences in coverage) was also associated with a global decrease in overall methylation across cancers, an observation for which there would be parallels in other contexts from previous studies, as described below. In experimental models, genome-wide hypomethylation has been repeatedly observed in structurally unstable cancer genomes [36, 37]. In the human germline, hypomethylation of genomic DNA associates with local genomic instability and structural variation [38]. DNA methylation may provide a stabilizing effect in preventing chromosomal instability and translocations [36], with other possible mechanistic links including an observed activation of components of the base excision DNA repair pathway at the time of genome-wide DNA demethylation in primordial germ cells [39]. In our study, we observed increased expression in DNA damage response and of DNA methyltransferase genes with increasing structural variation burden, both of these perhaps representing a transcription program initiated without success to combat genomic instability. Elsewhere, genome-wide DNA hypomethylation despite an increase in DNA methyltransferase activity and gene-specific regional hypermethylation has been observed in cancer [40, 41]. In parallel with results from a recent pan-cancer genomic study of aneuploidy [29], our study finds structural variation burden to correlate with cell cycle gene expression and anti-correlate with immune cell infiltration. As suggested in the previous aneuploidy study, tumor cells with high numbers of genomic rearrangements may have to overcome or evade the immune response for tumors to progress.

This present study demonstrates the need to include DNA methylation profiling as a component of ongoing and future cancer genomics studies, where TCGA datasets at present represent a truly unique resource in terms of having multiple data platforms in addition to DNA sequencing being applied uniformly to the same samples. Our results identify a class of molecular alterations that would not currently be a component of ongoing personalized or precision medicine approaches. Our study provides a rich resource, whereby the mRNA and DNA methylation associations provided here may be further explored to establish novel cancer drivers and mechanistic links with cancer phenotypes.

## Methods

### Patient cohorts

The results here are based upon data generated by both The Cancer Genome Atlas (TCGA) Research Network and the International Cancer Research Consortium (ICGC). Combined whole genome sequence (WGS) analysis and RNA-seq analysis was carried out for 2334 cases in total, 1892 of which were from TCGA and 1232 of which (including all ICGC cases and 790 TCGA cases) were included as part of the Pan-Cancer Analysis of Whole Genomes (PCAWG) consortium efforts. Cases profiled spanned a range of cancer types (bladder, sarcoma, breast, liver-biliary, cervix, leukemia, colorectal, lymphoma, prostate, esophagus, stomach, central nervous system or “cns”, head/neck, kidney, lung, skin, ovary, pancreas, thyroid, uterus), as detailed in Additional file 2. Of the 2334 cases with WGS and RNA-seq, 1482 were cases from TCGA that were also uniformly profiled for DNA methylation using Illumina 450K array platform. All publication moratoriums as set by the respective consortiums for utilizing these data were respectfully followed, with the PCAWG datasets being the last to have their moratorium lifted as of July 25, 2019.

### Somatic structural variant (SSV) data

SSV calls were compiled from three different sources: from the PCAWG consortium of high-pass WGS data on 1232 cases [4], from our own recent study utilizing SSV calls based on low-pass WGS data of 1207 cases [3], and from SSV calling by our group of 764 cases with high-pass WGS, using Meerkat algorithm [2]. All coordinates are based on the hg19 human reference genome. Low-pass WGS involved sequencing at ∼ 6–8× coverage, while high-pass WGS involved sequencing at ∼ 30–60× coverage. Somatic variants were defined by comparison between the tumor and matched normal. For PCAWG, SSV calls were made by three different data centers using different algorithms; calls made by at least two algorithms were used in the downstream analyses, along with additional filtering criteria being used as described by the PCAWG consortium [42, 43]. Low-pass WGS calls were previously made using BreakDancer [44] and Meerkat [2] algorithms [3]. SSV calling of high-pass WGS by Meerkat algorithm was carried out as previously described [3, 13, 45]. Briefly, the combined discordant read pairs support and reads spanning the breakpoint junction was at least six for each event. For each variant, if there was more than one discordant read pair supporting the variant in any normal samples of the same tumor type, such variant was considered germline and removed from the somatic variant list. Alterations found in simple or satellite repeats were also excluded from the output. Previous studies show that on the order of 96–98% of high confidence SSVs from high-pass WGS data detected by Meerkat can be validated by PCR [2, 13].

All available SSV calling information was brought together as described above in the interests of the downstream integrative analyses with both gene expression and DNA methylation. True SSV calls missed by one calling dataset would potentially be supplemented by inclusion of results from another dataset. As one notable example, of the five cases of chromophobe renal cell carcinoma (KICH) for which an SSV breakpoint upstream of *TERT* was both identified by Meerkat WGS analysis and independently confirmed by PCR [13], just three cases had the same SSV breakpoints detected by PCAWG analysis, consistent with observations elsewhere that ensembles of SSV analysis pipelines do not always outperform individual SSV calling methods [46]. In addition, low-pass WGS calls, in particular, would involve a high false-negative rate (with ~ 20% of the SSV calls by high-pass data being identifiable using the low-pass data [3]), where 174 cases in our compilation dataset had SSV calls by both high-pass and low-pass WGS. The false-negative SSV call rates represented by variable sequencing coverage and calling methods across the different TCGA and ICGC sequencing projects were offset in part by the inclusion of greater numbers of cases and associated increased statistical power involved in the integrative analyses. The integration of results between orthogonal platforms (e.g., WGS and RNA-seq and WGS and DNA methylation) was therefore a key aspect of our study, as associations identified must be significant enough to rise above any noise involving the respective data platforms.

In addition, we checked the Database of Genomic Variants (DGV) germline call set (2016 hg19 version), consisting of 392,583 structural variants, against our SSV call set, consisting of nearly 270,000 SSVs. Exactly four SSVs from our set overlapped with DGV, and none of these four were actually involved in the top significant mRNAs or CGI methylation features by distance metric method (i.e., either the questionable SSVs were not anywhere in proximity to a gene or CGI called significant, or, in the one case where an SSV was near a significant CGI, the associated sample was non-TCGA and did not have DNA methylation data and was therefore not used in the DNA methylation analysis).

### Gene expression data

Gene expression calls based on RNA-seq data were obtained from two different sources: from PCAWG consortium and from TCGA Network. PCAWG expression calls were available for 1220 cases (including 442 ICGC cases and 778 TCGA cases), which data involved alignments by both STAR (version 2.4.0i,2-pass) and TopHat2 (version 2.0.12) were used to generate a combined set of calls, which efforts substantially reduced potential batch effects due to the use of different computational pipelines between ICGC and TCGA projects [47]. TCGA Network expression calls were uniformly quantified for all 1892 TCGA cases by counting the number of reads overlapping each gene model’s exons and converted to reads per kilobase mapped (RPKM) values by dividing by the transcribed gene length, defined in the GAF and by the total number of reads aligned to genes as previously described [48], with these data obtained from the Broad Institute Firehose pipeline (http://gdac.broadinstitute.org/). Combat software [49] was used to correct for batch effects represented by the two RNA-seq alignment and processing methods (PCAWG versus TCGA, using cancer type as the experimental group). Analysis of the combined RNA-seq dataset of 2334 cases both before and after batch correction (Additional file 1: Figure S1A) found that pre-Combat sample profiles segregate according to the processing method (i.e., batch) and post-Combat sample profiles segregate according to cancer type (representative of tumor biology). Duplicate expression profiles for a sample represented by both PCAWG and TCGA processing methods were averaged to make a single profile. The miRNA-seq dataset was obtained from TCGA PanCanAtlas project (https://gdc.cancer.gov/about-data/publications/pancanatlas) [50], which dataset involved batch correction as carried out by TCGA Network according to Illumina GAIIx or HiSeq 2000 platforms.

### Copy number alteration (CNA) data

Gene-level CNA calls were obtained from two different sources: from PCAWG consortium (based on WGS analysis) and from TCGA Network (based on SNP array analysis). DNA from each tumor or germline-derived sample had been hybridized by TCGA Network to Affymetrix SNP 6.0 arrays as previously described [48, 51]. Significant focal copy number alterations were identified from segmented data using GISTIC 2.0.22. The Broad Institute’s Firehose pipeline (http://gdac.broadinstitute.org/) first filtered out normal samples from the segmented copy number data by inspecting the TCGA barcodes and then executed GISTIC (Firehose task version: 140) to generate gene-level log base 2 (tumor/normal) CNA values as a continuous variable. Gene-level copy data based on WGS data was previously generated by PCAWG consortium from a consensus of multiple CNA callers [52]; log base 2 (tumor/normal) CNA values were then generated from the PCAWG copy calls by dividing the gene copy with the tumor ploidy. Combat software [49] was used to correct for batch effects represented by the two CNA datasets (PCAWG WGS-based versus TCGA SNP array-based, using cancer type as the experimental group, excluding genes on X or Y chromosomes). Duplicate CNA profiles for a sample represented by both WGS and SNP array were averaged to make a single profile. An index of overall CNA (Fig. 6e) was computed as the standard deviation of copy alteration logged ratios across all genes.

### Integrative analyses between SSVs and gene expression

Genes with altered expression associated with nearby SSV breakpoint were defined by two methods: by the “genomic region window” method, which has been previously described [3, 5], and by the “distance metric” method, which is described below. These analyses involved 17,798 unique named genes, where genes located on the X or Y chromosomes in particular were not included, along with genes not represented in the PCAWG gene-level copy number dataset. Both integrative analysis methods incorporated TCGA or ICGC project as a covariate, in order to factor in differences involving either WGS coverage or tissue-specific gene expression. While 1033 cases in our cohort with only low-pass WGS would entail lower sensitivity of SSV detection, the larger sample numbers utilized also provided increased power, which was better able to tolerate false-negative events and other sources of noise in identifying recurrent patterns. To an extent, our analytical approaches were also more tolerant of false negatives, whereby in our examining fixed genomic regions near a given gene, multiple SSV breakpoints may exist, but only one would need to have been identified by WGS in order to contribute to associations found. In addition, where high-pass vs low-pass WGS was explicitly incorporated as a covariate, no substantial differences in the results were observed (e.g., Additional file 1: Figures S3A and S6A).

The genomic region window method starts with a number of specified genomic region windows of interest in relation to genes, where for each region we constructed an SSV breakpoint matrix by annotating for every sample the presence or absence (using “1” or “0”, respectively) of at least one SSV breakpoint within the given region. For the set of SSVs associated with a given gene within a specified region in proximity to the gene (e.g., 0–20 kb upstream, 20–50 kb upstream, 50–100 kb upstream, 0–20 kb downstream, 20–50 kb downstream, 50–100 kb downstream, or within the gene body), correlation between expression of the gene and the presence of an SSV breakpoint was assessed using a linear regression model (with log-transformed expression values). In addition to modeling expression as a function of SSV event, cancer type (as encapsulated by one of the 30 TCGA or ICGC projects listed in Additional file 2) was incorporated into the model as a covariate, where any significant association between genes and SSV breakpoint pattern must rise above any association that would be explainable by cancer type alone. As presented in the “Results” section, other models incorporating additional covariates of interest, such as CNA (using log2 tumor/normal values) were considered, where observed associations between expression and SSV pattern would be required to rise above what would be explainable by the additional covariates in addition to cancer type. For these linear regression models, genes with at least three samples associated with an SSV within the given region were considered. Genes for which SSV associations were significant (FDR < 5%) after correcting for both cancer type and CNA were explored in downstream analyses.

The distance metric method is similar to the genomic region windows approach, but with the gene X sample breakpoint pattern matrix being constructed in a different way. In defining a ± 1 Mb region window in relation to each gene (spanning from 1 Mb upstream of the gene start to 1 Mb downstream of the gene start), for each sample, the relative distances of the SSV breakpoint closest to the start of each gene were tabulated, with a gene X sample relative distance matrix being assembled. Where no breakpoints were found for a particular gene in a given sample, the maximum distance of 1 Mb was imputed. Using this breakpoint pattern matrix (with the absolute relative distances being log2-transformed), the correlation between expression of the gene and the presence of an SSV breakpoint was assessed using a linear regression model (with log-transformed expression values). As with the genomic region windows method, cancer type (i.e., TCGA or ICGC project) and other additional covariates of interest, such as CNA (using log2 tumor/normal values) were incorporated into the linear models. The distance metric method was also used to evaluate the associations of microRNAs with SSV breakpoint patterns, as well as for identifying gene-level SSV associations within each of the 20 major cancer types (cancer type here being more broadly defined according to tissue of origin, following the classifications provided by PCAWG consortium [4]).

In all of the linear models performed in this study, appropriate data transformations were used to make the data align better with the model assumptions. As noted above, gene expression data were log2-transformed, as were the relative SSV breakpoint distances from the gene in the breakpoint pattern matrix. For DNA methylation beta values, the logit transformation was used, also a common practice for making DNA methylation data better align with linear model assumptions [53].

### Integrative analyses between SSVs and DNA methylation

DNA methylation profiles had been generated by TCGA using the Illumina Infinium HumanMethylation450 (HM450) BeadChip array platform (Illumina, San Diego, CA), as previously described [48]. Patterns of association of altered DNA methylation with nearby SSV breakpoint focused on the 111,203 array probes falling within CpG islands (CGIs) that did not involve X or Y chromosomes (these chromosomes not being included as these would be present or not present or differentially methylated according to patient gender). Both the genomic regions window method and the distance metric method (both described above) were applied to the DNA methylation data in a similar manner as that of the gene expression data. The gene X sample breakpoint matrices as constructed above were joined to the DNA methylation data matrix, in terms of the genes associated with CGIs. The correlation between methylation of each CGI and the presence of an SSV breakpoint in relation to the CGI-associated gene was assessed using linear regression models (with logit-transformed DNA methylation beta values). As with the gene expression analyses, cancer type (i.e., TCGA or ICGC project) and other additional covariates of interest, such as CNA (using log2 tumor/normal values), were incorporated into the linear models.

We searched the subset of SSV-CGI associations involving higher or lower DNA methylation (with CGI probe being globally significant by distance metric method and methylation being higher or lower in that particular sample), for those that may involve the rearrangement of regions from other parts of the genome normally having high or low methylation. From TCGA, HM450 methylation profiles of normal adjacent tissues were examined, where for each cancer type, the corresponding normal sample profiles for that tissue type were assigned to represent a surrogate for normal methylation (e.g., TCGA-BRCA for breast and TCGA-PRAD for prostate). For a given SSV breakpoint, the normal tissue DNA methylation levels at the CGI site (nearby the gene on the other side of the SSV breakpoint) were compared with the average DNA methylation represented by the rearranged region adjacent to the breakpoint (using window of 50 kb, averaging the normal methylation beta values for all HM450 probes within this region). The average difference in methylation beta values (CGI versus average of adjacent rearranged region) was computed for each SSV-CGI association (with only the SSV breakpoint closest to the start of each gene being considered for each sample in the instance of multiple breakpoints being detected).

### Integrative analyses using TAD and enhancer genomic coordinates

To identify breakpoints associated with TAD disruption, we used recently published TAD data from the IMR90 cell line [27], where TADs have been found to be largely invariant across cell types [23]. TAD-disrupting SSVs were defined as those SSVs for which the two breakpoints did not fall within the same TAD. The fraction of SSVs involving topologically associated domain (TAD) disruption was evaluated both for SSVs with breakpoints located in proximity to a gene (e.g., 0–100 kb upstream, 0–100 kb downstream, or within gene body) and associated with its altered expression and for SSVs with breakpoints located in proximity to a gene and associated with altered methylation of the nearby CGI, with significance of enrichment as compared to the entire set of SSVs being evaluated by chi-squared test.

For each SSV breakpoint association 0–500 kb upstream of a gene (each association involving unique breakpoint and gene pairing, with only the SSV breakpoint closest to the start of each gene being considered for each sample in the instance of multiple breakpoints being detected), the potential for translocation of an active, in vivo-transcribed enhancer near the gene that would be represented by the rearrangement was determined (based on the orientation of the SSV breakpoint mate). We utilized the enhancer annotations as provided by Andersson et al. [28]. The Andersson study had previously categorized a set of ~ 40 K enhancers according to tissue- or cell-specific expression, with a small subset of enhancers categorized as “ubiquitous” or associated with expression in the majority of tissue and cell types examined. The ubiquitous enhancers were therefore applied to all cases in our pan-cancer cohort. In addition, for each one of the 20 major cancer types represented in our study, any applicable Andersson tissue- or cell-specific enhancer subsets for that particular cancer type were also applied (e.g., mammary epithelial cell-specific enhancers for breast cancer cases, epithelial cell of prostate and prostate gland for prostate cancer cases, see Additional file 9). Only enhancers that were either ubiquitous or with tissue or cell specificity relevant to a given cancer type were applied to the SSVs found for cases of that cancer type.

SSV breakpoint-to-gene associations involving the translocation of an active, in vivo-transcribed enhancer within 0.5 Mb of the gene (assuming no other disruptions involving the region), where the unaltered gene had no enhancer within 1 Mb, were tabulated. Only SSVs with breakpoints on the distal side from the gene were considered in this analysis; in other words, for genes on the negative strand, the upstream sequence of the breakpoint (denoted as positive orientation) should be fused relative to the breakpoint coordinates, and for genes on the positive strand, the downstream sequence of the breakpoint (denoted as negative orientation) should be fused relative to the breakpoint coordinates. Percentages of SSV breakpoint associations involving the translocation of an active, in vivo-transcribed enhancer were tabulated for the subsets of SSV breakpoint associations involving altered gene expression or altered DNA methylation, with evaluation of enrichment as compared to results from the entire set of SSVs being made using chi-squared tests.

### Molecular correlates of the overall extent of genomic rearrangement

SSV calls across the three datasets were collapsed to estimate the number of unique SSVs detected per sample; SSVs less than 10 bases apart were collapsed into a single SSV. For each gene, the correlation between expression and the total number of SSV events detected across the 2334 cases with RNA-seq data was assessed, using linear regression models with both log-transformed expression values and log-transformed SSV event numbers, correcting for low-pass versus high-pass WGS (a technical factor impacting SSV detection) as well as for other specific covariates where indicated. In a similar manner, CGI probes showing correlation between DNA methylation and the total number of SSV events were determined (using logit-transformed DNA methylation values), across the 1482 cases with methylation data. A global SSV index variable, which measured the total number of SSV events while correcting for high-pass versus low-pass WGS, was defined first by taking the log-transformed total SSV numbers within samples with only low-pass WGS (*n* = 1033) and normalizing these values across samples to standard deviations from the median and then by doing the same for the remaining samples with high-pass WGS. Scoring for a transcriptional signature of DNA damage response pathway was carried out by taking a set of 49 genes canonically associated with the pathway [30] and taking the average of the normalized expression values (standard deviations from the median of logged values) for each sample profile.

### Pathway and signature analyses

Enrichment of GO annotation terms within sets of differentially expressed genes was evaluated using SigTerms software [54] and one-sided Fisher’s exact tests. “Cancer-related genes,” as annotated in gene significance plots, are genes with membership in the Sanger Cancer Consensus Gene list (http://www.sanger.ac.uk/science/data/cancer-gene-census) [14], genes significant by pan-cancer mutation significant analysis (from ref [15]), or genes in annotated pathways targeted by mutation in ref [16] (using Additional file 7 of that reference). To computationally infer the infiltration level of specific immune cell types using RNA-seq data (Fig. 7a), we used the set of genes specifically overexpressed in the given immune cell type according to the study from Bindea et al. [31]; expression values were first normalized within each cancer type, and the average of normalized expression values within each sample profile was computed for each immune signature. We also performed signature analyses using a previously curated list of 276 genes encompassing all major DNA repair pathways [30]; both the full set of 276 genes represented in our RNA-seq data and gene subsets in core DNA damage response pathways (using the “core” pathway membership gene sets as provided in [30]) were evaluated, with expression values being first normalized across sample profiles, with the average of normalized expression values within each sample profile being computed for the given DNA repair signature.

### Statistical analysis

All *p* values were two-sided unless otherwise specified. Linear regression models were utilized to associate the expression or methylation of genes with nearby SSV breakpoints and with structural variation burden, as described above. One-sided Fisher’s exact tests or chi-squared tests were used to determine significance of overlap between two given feature lists. The method of Storey and Tibshirani [17] was used to estimate FDR for significant genes. FDR cutoffs of 5% were typically used to define top molecular features, though in situations where fewer samples were involved (e.g., the cancer type-specific analyses of Fig. 3) or where we examined the overlap between two results sets (e.g., the methylation versus expression results of Fig. 4d), more relaxed statistical cutoffs (e.g., FDR < 10%) were used. Visualization using heat maps was performed using JavaTreeview [55].

## Supplementary information

**Additional file 1: Figure S1.** Related to Fig. 1. Batch effects correction involving RNA-seq and DNA copy number alteration (CNA) datasets. **Figure S2.** related to Fig. 2. Additional information regarding the top set of genes with altered expression associated with nearby SSV breakpoint by distance metric method. **Figure S3.** related to Fig. 2. Addition information regarding genes with altered expression associated with nearby SSV breakpoint. **Figure S4.** related to Fig. 3. Additional information on genes with altered expression associated with nearby SSV breakpoint according to cancer type. **Figure S5.** related to Fig. 4. Additional information regarding the top set of CGIs with altered methylation associated with nearby SSV breakpoint by distance metric method. **Figure S6.** related to Fig. 4. Additional information on CGIs with altered DNA methylation associated with nearby SSV breakpoint. **Figure S7.** related to Fig. 5. DNA methylation patterns by cancer type and tissue type. **Figure S8.** related to Fig. 6. Additional information regarding global molecular alterations associated with the overall burden of structural variation across cancers.

**Additional file 2.** Related to Fig. 1. TCGA and ICGC cancer cases examined in this study.

**Additional file 3.** Related to Fig. 1. SSV calls by Meerkat for 827 TCGA cases with high pass WGS data, for 761 of which RNA-seq data were available for analysis as part of the total combined set of 2334 cases.

**Additional file 4.** Related to Fig. 1. By genomic region windows method: complete set of gene-level correlations between expression and nearby SSV event, according to region examined (e.g. 0-20 kb upstream, 20-50 kb upstream, 50-100 kb upstream, 0-20 kb downstream, 20-50 kb downstream, 50-100 kb downstream, or within the gene body) and the regression model applied.

**Additional file 5.** Related to Fig. 2. By distance metric method: complete set of gene-level correlations between expression and nearby SSV event, according to regression model applied (i.e. what covariates were considered). The top significant mRNAs (FDR < 10%, correcting for cancer type and CNA) and related information are highlighted in a separate Excel tab. Results for microRNAs are provided in a separate Excel tab. Also includes Gene Ontology (GO) terms associated with genes positively correlated (FDR < 5%, with corrections for cancer type and CNA) with occurrence of SSV in proximity to the gene.

**Additional file 6.** Related to Fig. 3. By distance metric method: complete set of gene-level correlations between expression and nearby SSV event for each individual cancer type (using regression model with corrections for cancer type and CNA). Data for the 893 CGI DNA methylation probes showing both high cancer type-specific methylation and significant positive correlation between expression and SSV breakpoint for any one of the 20 cancer types surveyed (from Fig. 3d) are in a separate data tab.

**Additional file 7.** Related to Fig. 4. Complete set of probe-level CGI correlations between DNA methylation and nearby SSV event, according to regression model applied (i.e. what covariates were considered). Results by both genomic region windows method (e.g. 0-100 kb upstream, 0-100 kb downstream, or within the gene body) and distance metric method are included. The top significant mRNAs (FDR < 5% by distance metric method, correcting for cancer type and CNA) and related information are highlighted in a separate Excel tab.

**Additional file 8.** Related to Fig. 5. SSV associations with disruption of TADs. All SSVs involving TAD disruption (i.e. SSVs with breakpoints spanning TAD boundaries), as well as the subset of SSVs with breakpoints located in proximity to a gene (0-100 kb upstream, 0-100 kb downstream, or within the gene body) and associated with altered expression or methylation, are included.

**Additional file 9.** Related to Fig. 5. SSV associations with translocated active in vivo-transcribed enhancers. For the enhancer-related analyses of Fig. 5b, the Andersson et al. tissue- or cell-specific enhancer subsets as applied to each TCGA or ICGC project are listed. Results include the subset of SSV breakpoint associations involving the translocation of an active, in vivo-transcribed enhancer within 0.5 Mb of the gene (where the unaltered gene had no enhancer within 1 Mb), for both the entire set of SSV breakpoint associations occurring 0-500 kb upstream of a gene and with breakpoint mate on the distal side from the gene (for cases with WGS), as well as for the subset of SSV breakpoint associations involving altered gene expression or CGI methylation.

**Additional file 10.** Related to Fig. 5. SSVs associated with rearrangement of regions with high or low methylation. Using a dataset of DNA methylation of normal tissues, the average DNA methylation represented by the rearranged region (using window of 50 kb) was compared with that of the CGI nearby the gene on the other side of the SSV breakpoint, with the average difference in methylation beta values computed for all SSV-CGI associations, as well as for the subset of SSV-CGI associations involving higher or lower DNA methylation (defined using distance metric FDR < 5% and methylation in cancer sample either > 0.4SD or < − 0.4SD from sample median for the case harboring the breakpoint).

**Additional file 11.** Related to Fig. 6. Complete set of molecular-level correlations with total number of SSV events detected across samples, according to regression model applied (i.e. what covariates were considered, all models including cancer type according to TCGA/ICGC project and high pass versus low pass WGS as covariates). DNA methylation and mRNA results are included in separate data tabs. Also includes Gene Ontology (GO) terms associated with genes positively correlated (FDR < 1%, with corrections for cancer type, CNA, and low pass versus high pass WGS) with the total number of SSV events.

**Additional file 12.** Review history.

## Data Availability

All data used in this study are publicly available. For PCAWG data, SV calls, copy number data, and expression data are available at the ICGC Data Portal (https://dcc.icgc.org/pcawg). For TCGA data, SV calls for high-pass WGS are available in Additional file 3, and SV calls for low-pass WGS are available in the supplementary data of ref [3]; TCGA expression and SNP array-based CNA data are available from the Broad Institute Firehose pipeline (http://gdac.broadinstitute.org/). Source code in R for the linear modeling integrating SSV with expression data, with example data files, is available at Github https://github.com/chadcreighton/SVexpression_integration [56].
