# Supplementary material for: Global impact of somatic structural variation on the DNA methylome of human cancers
Source: Genome Biol. 2019 Oct 15;20:209. doi: 10.1186/s13059-019-1818-9 (PMC6792267; doi:10.1186/s13059-019-1818-9)
Supplement: Supplementary file 1 — Additional file 1: Figure S1. Related to Fig. 1. Batch effects correction involving RNA-seq and DNA copy number alteration (CNA) datasets. Figure S2. related to Fig. 2. Additional information regarding the top set of genes with altered expression associated with nearby SSV breakpoint by distance metric method. Figure S3. related to Fig. 2. Addition information regarding genes with altered expression associated with nearby SSV breakpoint. Figure S4. related to Fig. 3. Additional information on genes with altered expression associated with nearby SSV breakpoint according to cancer type. Figure S5. related to Fig. 4. Additional information regarding the top set of CGIs with altered methylation associated with nearby SSV breakpoint by distance metric method. Figure S6. related to Fig. 4. Additional information on CGIs with altered DNA methylation associated with nearby SSV breakpoint. Figure S7. related to Fig. 5. DNA methylation patterns by cancer type and tissue type. Figure S8. related to Fig. 6. Additional information regarding global molecular alterations associated with the overall burden of structural variation across cancers. [file 13059_2019_1818_MOESM1_ESM.pdf]

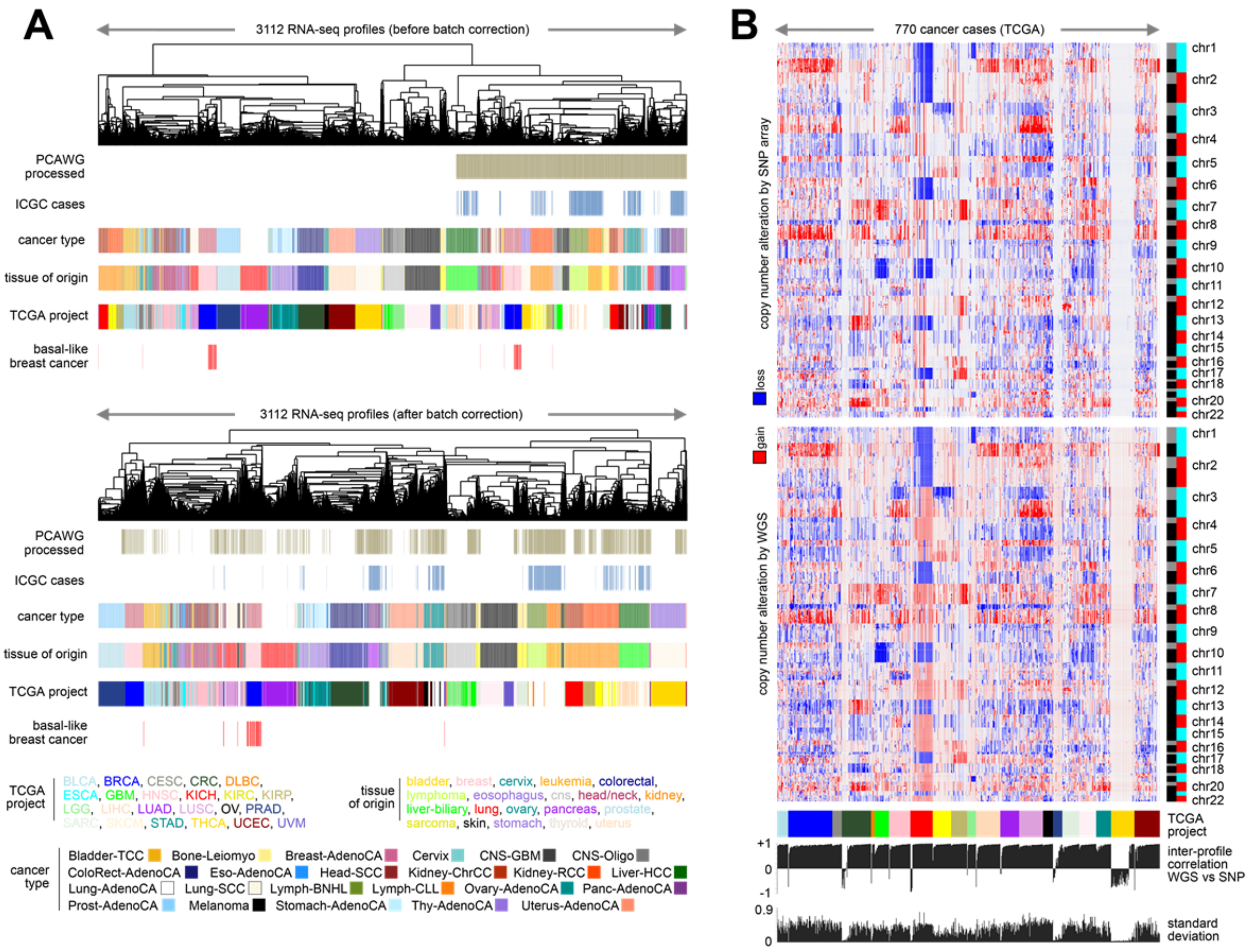

Fig. S1, related to Fig. 1. Batch effects correction involving RNA-seq and DNA copy number alteration (CNA) datasets. (A) Of the 2334 cases with RNA-seq data used in the present study, a portion (including 442 ICGC cases and 778 TCGA cases) were previously processed as part of the Pan-Cancer Analysis of Whole Genomes (PCAWG) consortium efforts. All TCGA cases were previously processed as part of TCGA consortium efforts. ICGC cases were part of the PCAWG processing efforts but were not part of TCGA efforts. Combat software [1] was used to correct for batch effects represented by the two RNA-seq alignment and processing methods (PCAWG versus TCGA). Hierarchical clustering of the top 2000 most variable genes was carried out on the combined RNA-seq data both before and after batch correction (represented by top and bottom clustering trees, respectively), where pre-Combat sample profiles segregate according to processing method and post-Combat sample profiles

segregate according to cancer type. **(B)** For cases included as part of the PCAWG consortium efforts (including all ICGC cases), gene-level CNA was estimated using WGS. TCGA cases (including cases not included in PCAWG) also had gene-level CNA previously estimated using SNP array platform. Combat software was used to correct for batch effects represented by the two gene-level CNA methods. For the 770 cases in TCGA with both WGS and SNP array gene-level CNA estimates, the CNA values were collapsed into cytobands, with the post-Combat data for both SNP array and WGS estimates being shown here (top and bottom CNA heat maps, respectively). For the vast majority of samples, a high cytoband-level CNA inter-profile correlations (Pearson's) was observed between SNP and WGS post-Combat, with the notable exception of cases showing little or no global CNA (where noise is essentially being modeled in the inter-profile correlation).

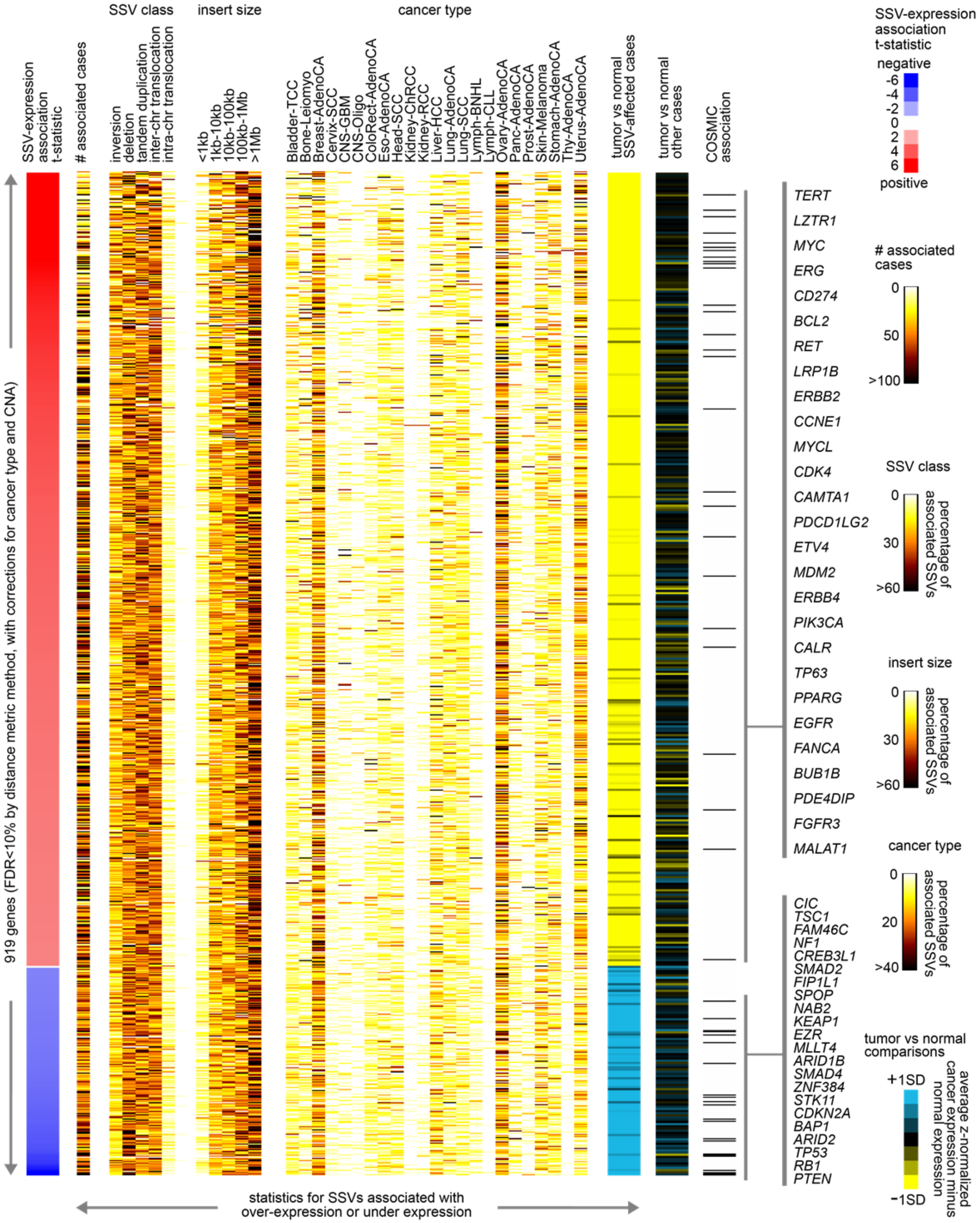
SSV class
insert size
cancer type
SSV-expression association t-statistic
associated cases
inversion
deletion
tandem duplication
inter-chr translocation
intra-chr translocation
<1kb
1kb-10kb
10kb-100kb
100kb-1Mb
>1Mb
Bladder-TCC
Bone-Leiomyo
Breast-AdenoCA
Cervix-SCC
CNS-GBM
CNS-Oligo
ColoRect-AdenoCA
Eso-AdenoCA
Head-SCC
Kidney-ChRCC
Kidney-RCC
Liver-HCC
Lung-AdenoCA
Lung-SCC
Lymph-BNHL
Lymph-CLL
Ovary-AdenoCA
Panc-AdenoCA
Prost-AdenoCA
Skin-Melanoma
Stomach-AdenoCA
Thy-AdenoCA
Uterus-AdenoCA
tumor vs normal SSV-affected cases
tumor vs normal other cases
COSMIC association
919 genes (FDR<10% by distance metric method, with corrections for cancer type and CNA)
statistics for SSVs associated with over-expression or under expression
TERT
LZTR1
MYC
ERG
CD274
BCL2
RET
LRP1B
ERBB2
CCNE1
MYCL
CDK4
CAMTA1
PDCD1LG2
ETV4
MDM2
ERBB4
PIK3CA
CALR
TP63
PPARG
EGFR
FANCA
BUB1B
PDE4DIP
FGFR3
MALAT1
CIC
TSC1
FAM46C
NF1
CREB3L1
SMAD2
FIP1L1
SPOP
NAB2
KEAP1
EZR
MLLT4
ARID1B
SMAD4
ZNF384
STK11
CDKN2A
BAP1
ARID2
TP53
RB1
PTEN
SSV-expression association t-statistic
negative
-6
-4
-2
0
2
4
6
positive
associated cases
0
50
>100
SSV class
0
30
>60
percentage of associated SSVs
insert size
0
30
>60
percentage of associated SSVs
cancer type
0
20
>40
percentage of associated SSVs
tumor vs normal comparisons
+1SD
-1SD
average z-normalized cancer expression minus normal expression

Fig. S2, related to Fig. 2. Additional information regarding the top set of genes with altered expression associated with nearby SSV breakpoint by distance metric method. Annotations are provided for the top set of genes associated with altered expression (FDR<10%, correcting for cancer type and CNA), according to numbers of associated cases (cases with SSV breakpoint within 1Mb, where the case also showed either altered expression>0.4SD from the sample median, for genes positively correlated with SSV breakpoint, or altered expression <-0.4SD from the median, for genes negatively correlated), representation by class and by size (size involving non-translocation SSVs) for the set of SSVs associated with altered expression, representation by cancer type for the affected cases, and tumor versus normal comparisons. For tumor-normal comparisons, samples with SSVs associated with altered expressed (>0.4SD from the median for positively correlated genes, <-0.4SD for negatively correlated genes) had their normalized expression compared with the average normalized expression of corresponding normal adjacent samples (where data were available, using TCGA data only). The gene-level average difference in z-normalized values between SSV-affected cancer cases and the corresponding normals are represented in the first yellow-blue heat map. The second heat map represents cancer versus normal differences for the remaining cases that do not appear impacted by SSVs. Genes highlighted by name are cancer-associated by COSMIC.

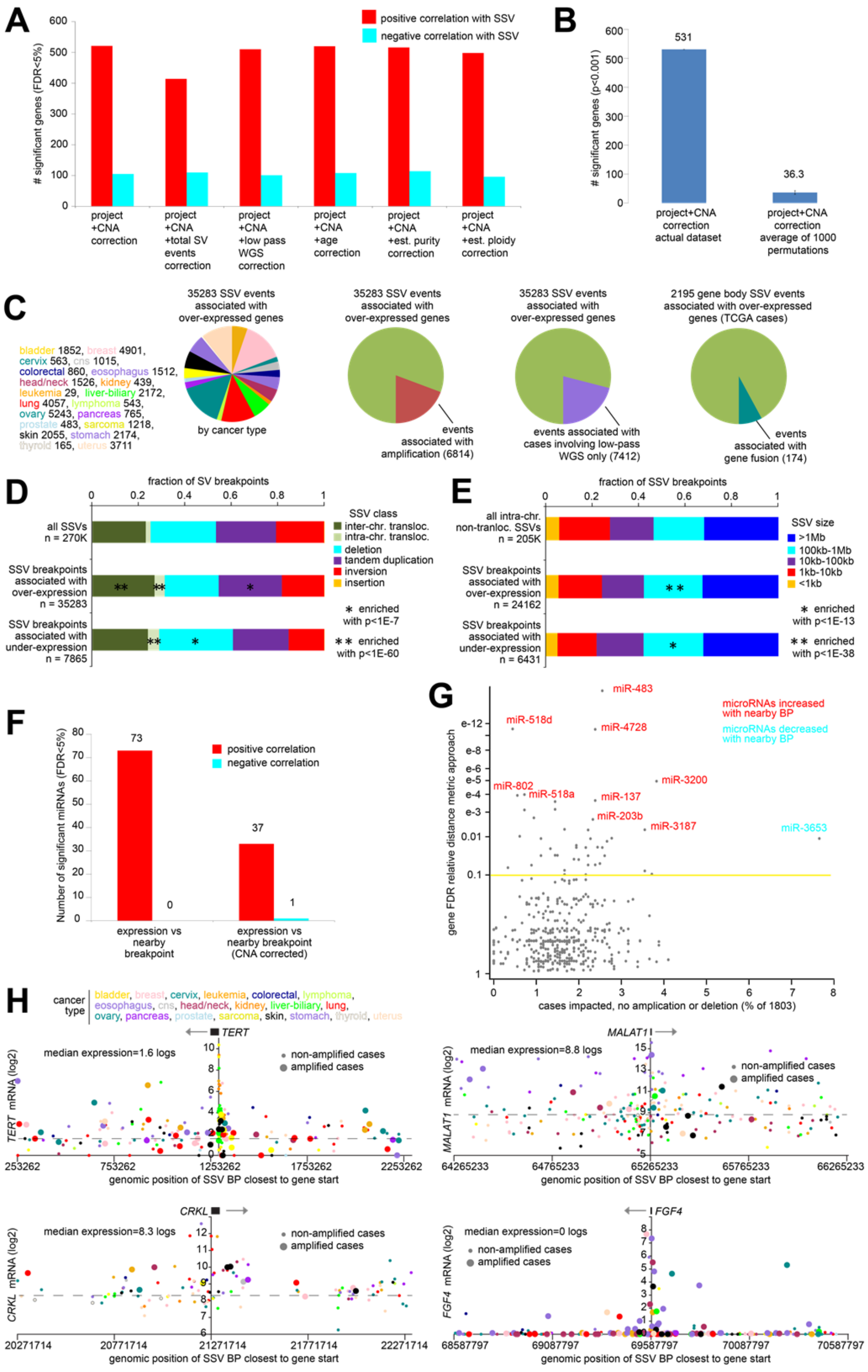

A
positive correlation with SSV
negative correlation with SSV
significant genes (FDR<5%)
project +CNA correction
project +CNA +total SV events correction
project +CNA +low pass WGS correction
project +CNA +age correction
project +CNA +est. purity correction
project +CNA +est. ploidy correction
B
significant genes (p<0.001)
531
36.3
project+CNA correction actual dataset
project+CNA correction average of 1000 permutations
C
35283 SSV events associated with over-expressed genes
by cancer type
bladder 1852, breast 4901, cervix 563, cns 1015, colorectal 860, eosophagus 1512, head/neck 1526, kidney 439, leukemia 29, liver-biliary 2172, lung 4057, lymphoma 543, ovary 5243, pancreas 765, prostate 483, sarcoma 1218, skin 2055, stomach 2174, thyroid 165, uterus 3711
events associated with amplification (6814)
events associated with cases involving low-pass WGS only (7412)
2195 gene body SSV events associated with over-expressed genes (TCGA cases)
events associated with gene fusion (174)
D
fraction of SV breakpoints
all SSVs n = 270K
SSV breakpoints associated with over-expression n = 35283
SSV breakpoints associated with under-expression n = 7865
SSV class
inter-chr. transloc.
intra-chr. transloc.
deletion
tandem duplication
inversion
insertion
* enriched with p<1E-7
** enriched with p<1E-60
E
fraction of SSV breakpoints
all intra-chr. non-tranloc. SSVs n = 205K
SSV breakpoints associated with over-expression n = 24162
SSV breakpoints associated with under-expression n = 6431
SSV size
>1Mb
100kb-1Mb
10kb-100kb
1kb-10kb
<1kb
* enriched with p<1E-13
** enriched with p<1E-38
F
Number of significant miRNAs (FDR<5%)
73
0
37
1
positive correlation
negative correlation
expression vs nearby breakpoint
expression vs nearby breakpoint (CNA corrected)
G
gene FDR relative distance metric approach
cases impacted, no amplification or deletion (% of 1803)
miR-483
miR-518d
miR-4728
miR-3200
miR-802
miR-518a
miR-137
miR-203b
miR-3187
miR-3653
microRNAs increased with nearby BP
microRNAs decreased with nearby BP
H
cancer type
bladder, breast, cervix, leukemia, colorectal, lymphoma, eosophagus, cns, head/neck, kidney, liver-biliary, lung, ovary, pancreas, prostate, sarcoma, skin, stomach, thyroid, uterus
TERT
median expression=1.6 logs
non-amplified cases
amplified cases
TERT mRNA (log2)
genomic position of SSV BP closest to gene start
MALAT1
median expression=8.8 logs
MALAT1 mRNA (log2)
CRKL
median expression=8.3 logs
CRKL mRNA (log2)
FGF4
median expression=0 logs
FGF4 mRNA (log2)

Fig. S3, related to Fig. 2. Addition information regarding genes with altered expression associated with nearby SSV breakpoint. (A) Numbers of significant genes (FDR<5% by distance metric method), showing correlation between expression and nearby SSV breakpoints, based on 2334 cases with RNA-seq data. Linear regression models evaluated significant associations when correcting for specific covariates, as indicated. **(B)** Results of permutation testing. In each of 1000 tests, we randomly shuffled the SSV event profiles and computed correlations with expression. Numbers of significant genes (p<0.001, distance metric method), showing correlation between expression and associated SSV event, are shown, both for the actual dataset and for the average of the permuted datasets (using corrections for both cancer type and gene-level CNA). **(C)** For 35283 events of an SSV breakpoint being associated with over-expression of a nearby gene (FDR<10% by distance metric method, with corrections for cancer type and CNA, and expression>0.4SD from median for the case harboring the breakpoint), breakdowns are provided according to cancer type, events involving amplification of the same gene (defined as log2 tumor/normal copy ratio >1), and events involving cases with low pass WGS data only. For 2195 gene body SSV events associated with gene over-expression (subset of TCGA cases only), the fraction of events involving a predicted gene fusion by RNA-seq analysis [2] is also indicated. **(D)** Breakdown by SSV class, for all SSVs in the dataset, for the 35283 events of an SSV breakpoint being associated with gene over-expression (from part C), and for the 7865 events of an SSV breakpoint being associated with gene under-expression (FDR<10% by distance metric method, with corrections for cancer type and CNA, and expression<-0.4SD from median for the case harboring the breakpoint). P-values by chi-square test. **(E)** Breakdown by SSV size for the SSV and SSV breakpoint sets represented in part D (excluding translocation SSVs). P-values by chi-square test. **(F)** Numbers of significant microRNAs (FDR<5% by distance metric method), when correcting for cancer type and when correcting for both cancer type and CNA. **(G)** Significance of microRNAs by distance metric method, as plotted (Y-axis) versus the percent of cases impacted (expression>0.4SD) by

nearby SSV breakpoint (within 1Mb) without associated amplification or deletion event (defined as log2 tumor/normal ratio >1 or <1, respectively). **(H)** As examples of significant genes, gene expression levels of *TERT*, *MALAT1*, *CRKL*, and *FGF4*, corresponding to SSVs located in the genomic region 1Mb downstream to 1Mb upstream of the given gene. Each point represents a single case (closest SSV breakpoint represented for each case). Cases with gene amplification are indicated.

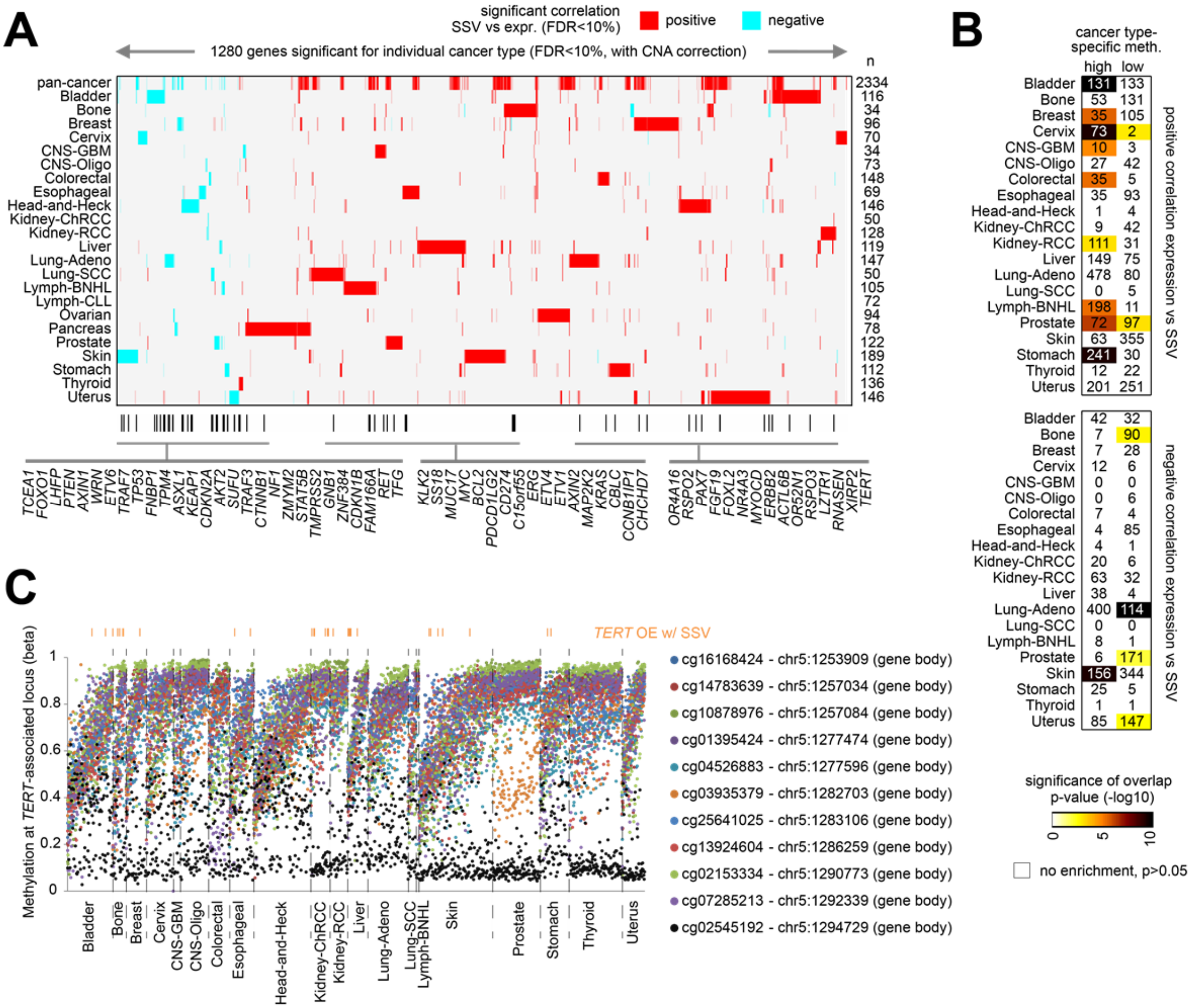

Fig. S4, related to Fig. 3. Additional information on genes with altered expression associated with nearby SSV breakpoint according to cancer type. (A) Heat map of differential t-statistics by cancer type, evaluating gene expression alterations with nearby SSV breakpoint (red, positive correlation with breakpoint; white, not significant with FDR>10%), for 1280 genes significant for one or more individual cancer types (FDR<10% by distance metric method, correcting for CNA). Genes listed by name are cancer related [3-5]. **(B)** For each cancer type, numbers of DNA methylation probes (Illumina 450K array platform) targeting CpG Islands (CGIs) with higher or lower methylation in the given cancer type versus other cancer types (FDR<0.001, t-test using logit-transformed data), for which the associated gene also

shows a positive or negative correlation between expression and nearby SSV breakpoint for that same cancer type (FDR<0.1, from part A). Any significance of overlap between the differential methylation results and the expression vs SSV results is indicated (p-values by chi-squared test). **(C)** Across the 1482 cases with DNA methylation data, with cases ordered by cancer type, the DNA methylation as measured by Illumina 450K probes targeting the *TERT* locus. Except for probe cg02545192, which targets a CpG site known to contain a repressive element in close proximity to the *TERT* core promotor [6, 7], the DNA methylation probes featured fall within the gene boundaries of *TERT*. Cases with *TERT* over-expression (“OE,” defined as >0.4SD from the median) are indicated.

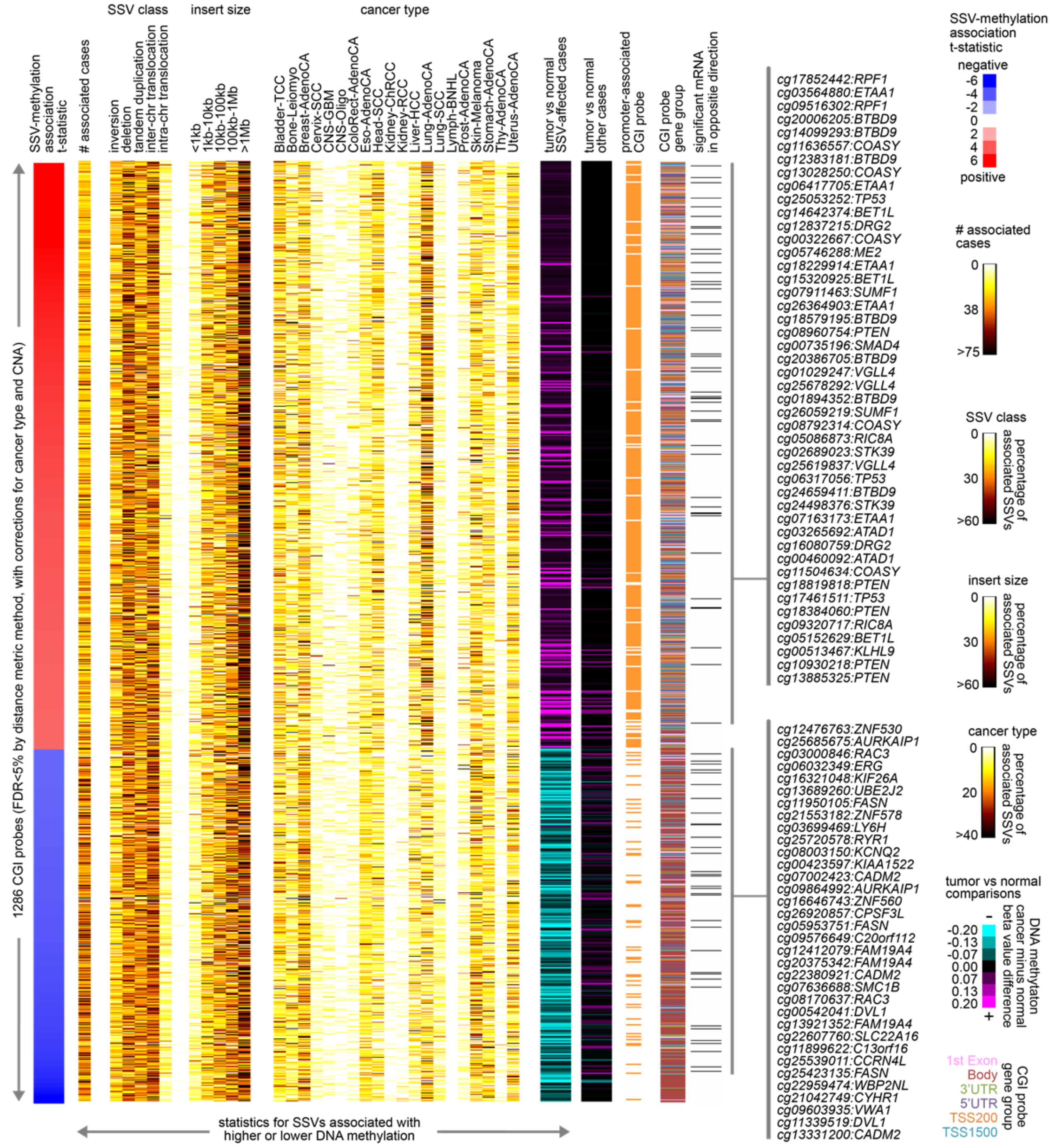

Fig. S5, related to Fig. 4. Additional information regarding the top set of CGIs with altered methylation associated with nearby SSV breakpoint by distance metric method.

Annotations are provided for the top set of CGI probes associated with altered methylation (FDR<5%, correcting for cancer type and CNA), according to numbers of associated cases

(cases with SSV breakpoint within 1Mb of the associated gene, where the case also showed either altered methylation>0.4SD from the sample median, for CGIs positively correlated with SSV breakpoint, or altered methylation <-0.4SD from the median, for CGIs negatively correlated), representation by class and by size (size involving non-translocation SSVs) for the set of SSVs associated with altered methylation, representation by cancer type for the affected cases, Ilumina 450K annotation for promoter-associated CGIs and gene group (1st Exon, Body, 3'UTR, 5'UTR, TSS200, TSS1500), and tumor versus normal comparisons. For tumor-normal comparisons, samples with SSVs associated with altered methylation (>0.4SD from the median for positively correlated CGIs, <-0.4SD for negatively correlated CGIs) had their methylation beta values compared with the average beta value of corresponding normal adjacent samples (where data were available). The cancer minus normal beta value difference between SSV-affected cancer cases and the corresponding normals are represented in the first purple-cyan heat map. The second heat map represents cancer versus normal differences for the remaining cases that do not appear impacted by SSVs. CGI probes/genes highlighted by name involve genes that had significant mRNA associations with SSV breakpoints (FDR<10% by distance metric method, correcting for cancer type and CNA) in the opposite direction from that of the CGI.

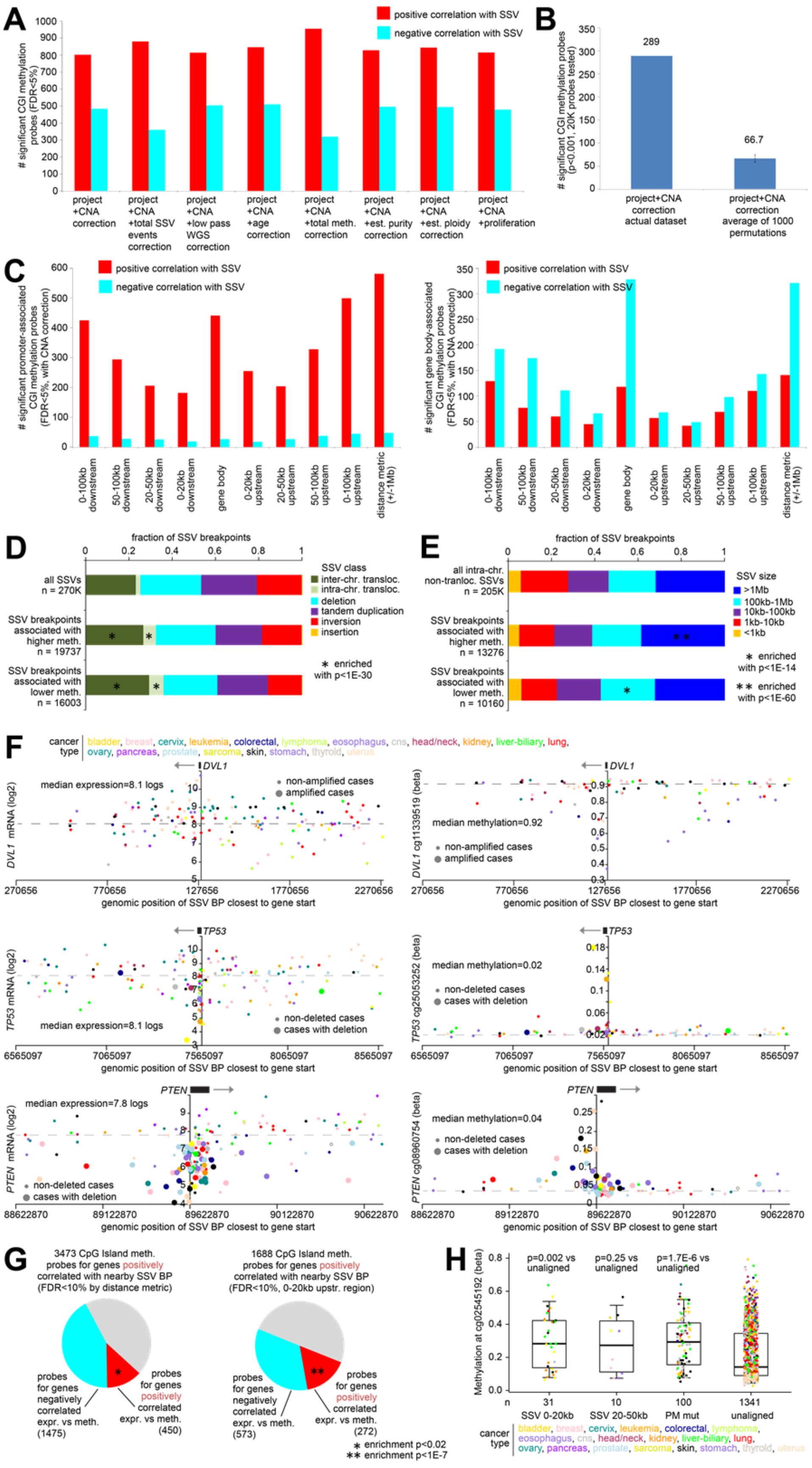

A
positive correlation with SSV
negative correlation with SSV
significant CGI methylation probes (FDR<5%)
project +CNA correction
project +CNA +total SSV events correction
project +CNA +low pass WGS correction
project +CNA +age correction
project +CNA +total meth. correction
project +CNA +est. purity correction
project +CNA +est. ploidy correction
project +CNA +proliferation
B
significant CGI methylation probes (p<0.001, 20K probes tested)
289
66.7
project+CNA correction actual dataset
project+CNA correction average of 1000 permutations
C
significant promoter-associated CGI methylation probes (FDR<5%, with CNA correction)
significant gene body-associated CGI methylation probes (FDR<5%, with CNA correction)
0-100kb downstream
50-100kb downstream
20-50kb downstream
0-20kb downstream
gene body
0-20kb upstream
20-50kb upstream
50-100kb upstream
0-100kb upstream
distance metric (+/-1Mb)
D
fraction of SSV breakpoints
all SSVs n = 270K
SSV breakpoints associated with higher meth. n = 19737
SSV breakpoints associated with lower meth. n = 16003
SSV class
inter-chr. transloc.
intra-chr. transloc.
deletion
tandem duplication
inversion
insertion
* enriched with p<1E-30
E
fraction of SSV breakpoints
all intra-chr. non-tranloc. SSVs n = 205K
SSV breakpoints associated with higher meth. n = 13276
SSV breakpoints associated with lower meth. n = 10160
SSV size
>1Mb
100kb-1Mb
10kb-100kb
1kb-10kb
<1kb
* enriched with p<1E-14
** enriched with p<1E-60
F
cancer type
bladder, breast, cervix, leukemia, colorectal, lymphoma, esophagus, cns, head/neck, kidney, liver-biliary, lung, ovary, pancreas, prostate, sarcoma, skin, stomach, thyroid, uterus
DVL1
median expression=8.1 logs
non-amplified cases
amplified cases
DVL1 mRNA (log2)
genomic position of SSV BP closest to gene start
median methylation=0.92
DVL1 cg11339519 (beta)
TP53
TP53 mRNA (log2)
median expression=8.1 logs
non-deleted cases
cases with deletion
median methylation=0.02
TP53 cg25053252 (beta)
PTEN
median expression=7.8 logs
PTEN mRNA (log2)
median methylation=0.04
PTEN cg08960754 (beta)
G
3473 CpG Island meth. probes for genes positively correlated with nearby SSV BP (FDR<10% by distance metric)
probes for genes negatively correlated expr. vs meth. (1475)
probes for genes positively correlated expr. vs meth. (450)
1688 CpG Island meth. probes for genes positively correlated with nearby SSV BP (FDR<10%, 0-20kb upstr. region)
probes for genes negatively correlated expr. vs meth. (573)
probes for genes positively correlated expr. vs meth. (272)
* enrichment p<0.02
** enrichment p<1E-7
H
Methylation at cg02545192 (beta)
p=0.002 vs unaligned
p=0.25 vs unaligned
p=1.7E-6 vs unaligned
n
31
10
100
1341
SSV 0-20kb
SSV 20-50kb
PM mut
unaligned
cancer type
bladder, breast, cervix, leukemia, colorectal, lymphoma, esophagus, cns, head/neck, kidney, liver-biliary, lung, ovary, pancreas, prostate, sarcoma, skin, stomach, thyroid, uterus

Fig. S6, related to Fig. 4. Additional information on CGIs with altered DNA methylation associated with nearby SSV breakpoint. (A) Numbers of significant CGIs (FDR<5% by distance metric method), showing correlation between DNA methylation and associated SSV event, across the 1482 cases with DNA methylation data. Linear regression models evaluated significant associations when correcting for specific covariates, as indicated. Cell proliferation covariate represents the average of the normalized values for a set of cell cycle genes from Whitfield et al. [8]. **(B)** Results of permutation testing. In each of 1000 tests, we randomly shuffled the SSV event profiles and computed correlations with DNA methylation (using 20K probes out of the 111K probes). Numbers of significant probes (p<0.001, distance metric method), showing correlation between methylation and associated SSV event, are shown, both for the actual dataset and for the average of the permuted datasets (using corrections for both cancer type and gene-level CNA). **(C)** For each of the indicated genomic region windows in relation to genes associated with CGIs, numbers of significant CGIs (FDR<5%, correcting for both cancer type and gene-level CNA) as taken from main Fig. 4A, broken down by promoter-associated CGIs (left) and gene body-associated CGIs (right). Gene body-associated denotes probes between the ATG and stop codon, irrespective of the presence of introns, exons, TSS, or promoters. **(D)** Breakdown by SSV class, for all SSVs in the dataset, for the 19737 events of an SSV breakpoint being associated with higher methylation (FDR<5% by distance metric method, with corrections for cancer type and CNA, and methylation>0.4SD from median for the case harboring the breakpoint), and for the 16003 events of an SSV breakpoint being associated with lower methylation (FDR<5% and methylation<-0.4SD from median). P-values by chi-square test. **(E)** Breakdown by SSV size for the SSV and SSV breakpoint sets represented in part D (excluding translocation SSVs). P-values by chi-square test. **(F)** As examples of significant genes, gene expression levels (left) and DNA methylation levels (right) for *DVL1, TP53,* and *PTEN*, corresponding to SSVs located in the genomic region 1Mb downstream to 1Mb upstream of the gene. Each point represents a single case (closest SSV

breakpoint represented for each case). Cases with gene amplification are indicated. **(G)** Observed association involving CGI methylation probes for genes positively correlated in expression with nearby SSV breakpoint (left, by distance metric method; right, by genomic region windows method) with genes positively correlated between expression and DNA methylation. P-value by chi-square test. **(H)** DNA methylation of the CpG site cg02545192 proximal to the *TERT* core promoter in cases with SSV breakpoint 0-20kb or 20-50kb upstream of *TERT*, in cases with *TERT* promoter (PM) activation mutation (SNV), and in the rest of cases (unaligned). P-values by t-test on logit-transformed methylation beta values. Box plots represent 5%, 25%, 50%, 75%, and 95%. Points in box plots are colored according to cancer type as indicated.

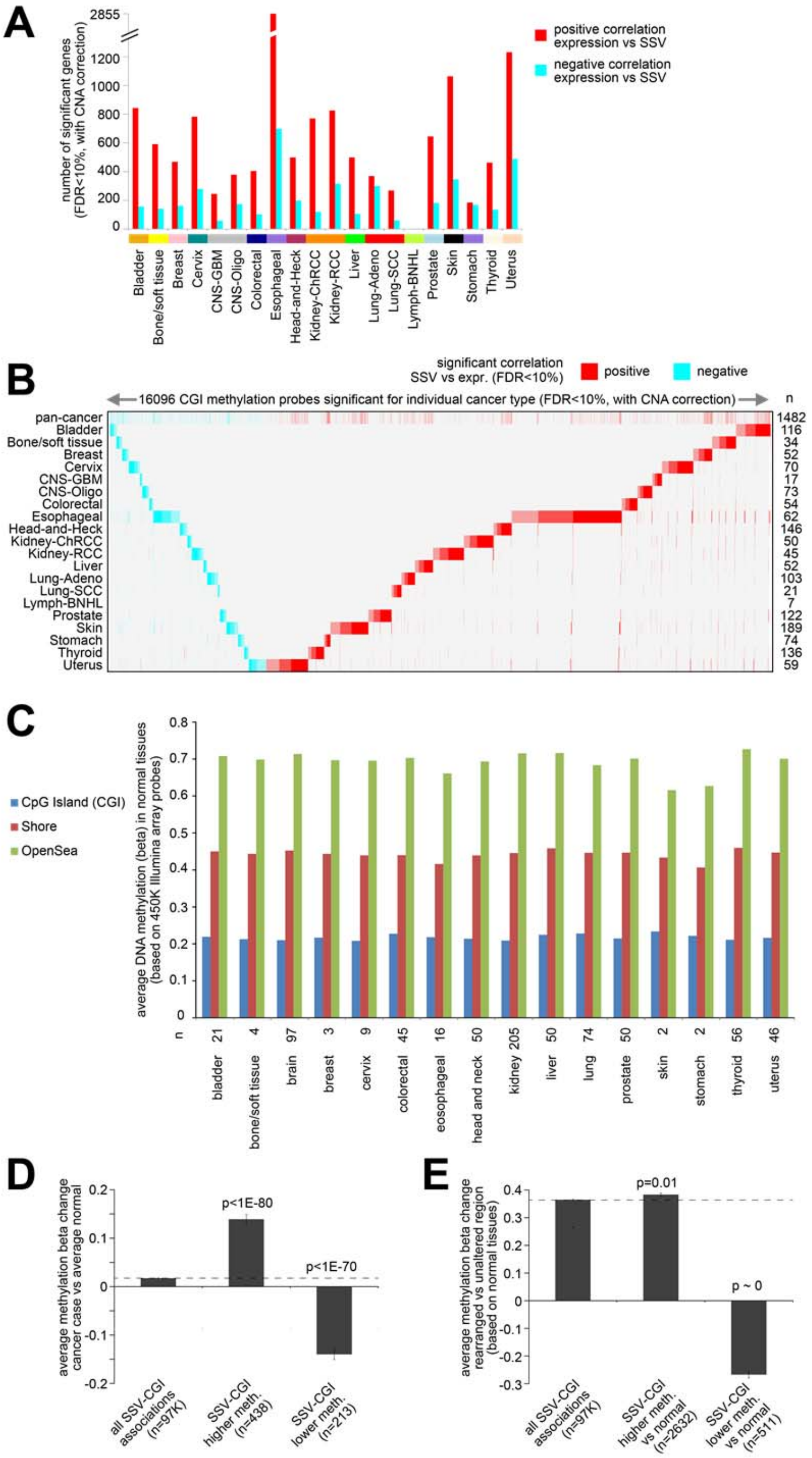

A
number of significant genes (FDR<10%, with CNA correction)
2855
1200
1000
800
600
400
200
0
positive correlation expression vs SSV
negative correlation expression vs SSV
Bladder
Bone/soft tissue
Breast
Cervix
CNS-GBM
CNS-Oligo
Colorectal
Esophageal
Head-and-Heck
Kidney-ChRCC
Kidney-RCC
Liver
Lung-Adeno
Lung-SCC
Lymph-BNHL
Prostate
Skin
Stomach
Thyroid
Uterus
B
significant correlation SSV vs expr. (FDR<10%)
positive
negative
16096 CGI methylation probes significant for individual cancer type (FDR<10%, with CNA correction)
n
pan-cancer 1482
Bladder 116
Bone/soft tissue 34
Breast 52
Cervix 70
CNS-GBM 17
CNS-Oligo 73
Colorectal 54
Esophageal 62
Head-and-Heck 146
Kidney-ChRCC 50
Kidney-RCC 45
Liver 52
Lung-Adeno 103
Lung-SCC 21
Lymph-BNHL 7
Prostate 122
Skin 189
Stomach 74
Thyroid 136
Uterus 59
C
average DNA methylation (beta) in normal tissues (based on 450K Illumina array probes)
CpG Island (CGI)
Shore
OpenSea
0.8
0.7
0.6
0.5
0.4
0.3
0.2
0.1
0
n
21 bladder
4 bone/soft tissue
97 brain
3 breast
9 cervix
45 colorectal
16 eosophageal
50 head and neck
205 kidney
50 liver
74 lung
50 prostate
2 skin
2 stomach
56 thyroid
46 uterus
D
average methylation beta change cancer case vs average normal
0.2
0.1
0
-0.1
-0.2
p<1E-80
p<1E-70
all SSV-CGI associations (n=97K)
SSV-CGI higher meth. (n=438)
SSV-CGI lower meth. (n=213)
E
average methylation beta change rearranged vs unaltered region (based on normal tissues)
0.4
0.3
0.2
0.1
0
-0.1
-0.2
-0.3
p=0.01
p ~ 0
all SSV-CGI associations (n=97K)
SSV-CGI higher meth. vs normal (n=2632)
SSV-CGI lower meth. vs normal (n=511)

Fig. S7, related to Fig. 5. DNA methylation patterns by cancer type and tissue type. (A) For each cancer type, numbers of CGI methylation probes showing correlation between expression and nearby SSV breakpoint (FDR<10% by distance metric method, linear model correcting for CNA). **(B)** Heat map of differential t-statistics by cancer type, evaluating CGI methylation alterations with nearby SSV breakpoint (red, positive correlation with breakpoint; white, not significant with FDR>10%), for 16096 CGI methylation probes significant for one or more individual cancer types (FDR<10% by distance metric method, correcting for CNA). **(C)** By normal adjacent tissue available in TCGA datasets, the average DNA methylation (beta) of 450K probes in normal tissues is shown, with methylation probes broken down by location with CpG Islands (CGI), Island "shores," or "Open sea" (i.e. not associated with CGIs). **(D)** Similar to main Fig. 5C, but comparing methylation of the SSV-harboring tumors to the corresponding normal-adjacent tissue (CGI nearby the gene on the other side of the SSV breakpoint), for the above SSV-CGI association subsets. P-values by Spearman's rank correlation. Bars represent standard error. **(E)** Similar to main Fig. 5C, but comparing methylation of SSV-harboring tumors to the normal-adjacent tissue to define the categories on the x-axis; methylation of SSV-harboring tumors was compared with corresponding normal, and all SSV-CGI associations for which the cancer-normal beta difference was either greater than 0.4 or <-0.4 were respectively examined for the normal tissue differences represented by rearranged region versus unaltered region. P-values by Spearman's rank correlation. Bars represent standard error.

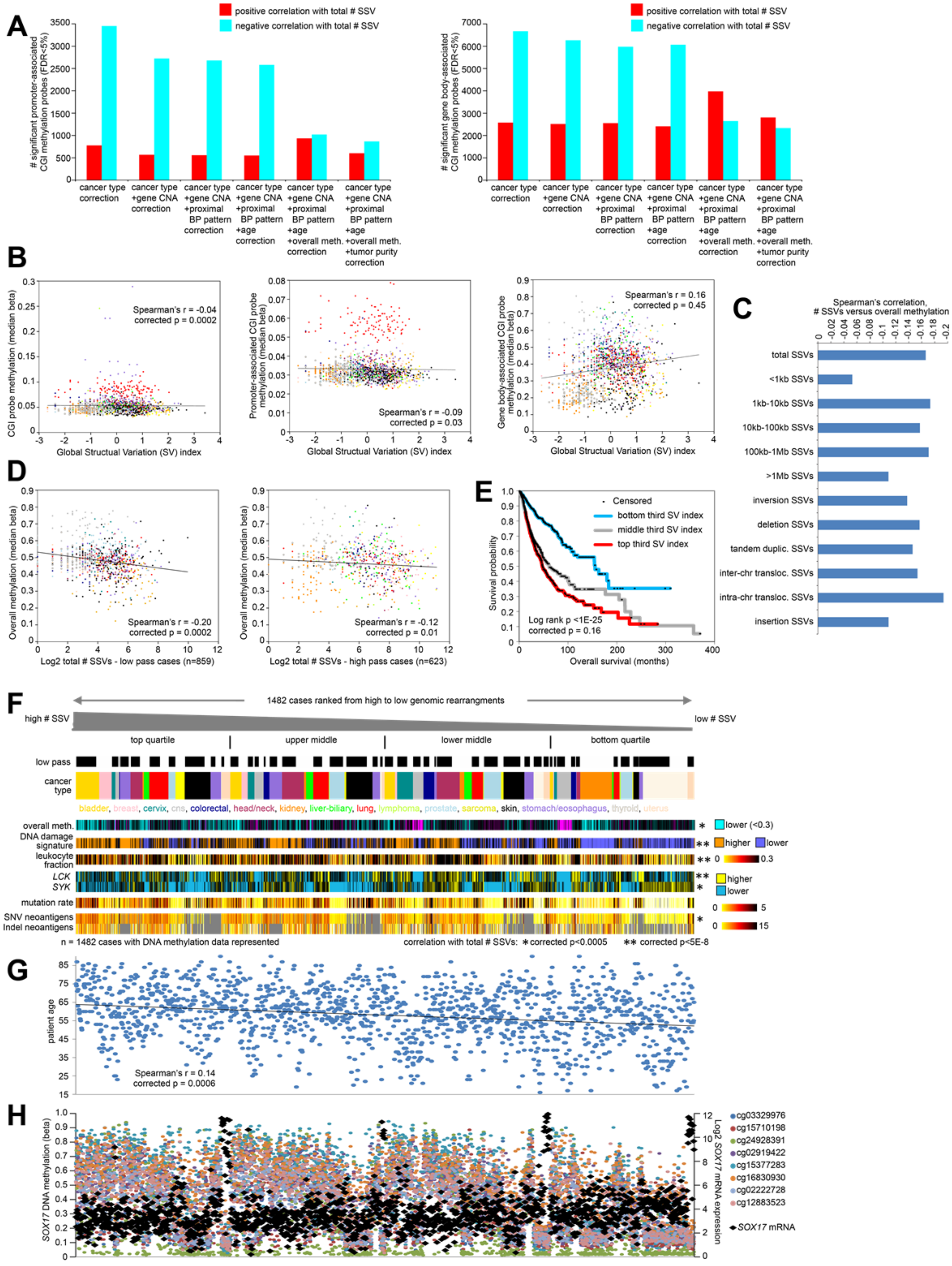

A
positive correlation with total # SSV
negative correlation with total # SSV
significant promoter-associated CGI methylation probes (FDR<5%)
significant gene body-associated CGI methylation probes (FDR<5%)
cancer type correction
cancer type +gene CNA correction
cancer type +gene CNA +proximal BP pattern correction
cancer type +gene CNA +proximal BP pattern +age correction
cancer type +gene CNA +proximal BP pattern +age +overall meth. correction
cancer type +gene CNA +proximal BP pattern +age +overall meth. +tumor purity correction
B
CGI probe methylation (median beta)
Promoter-associated CGI probe methylation (median beta)
Gene body-associated CGI probe methylation (median beta)
Global Structual Variation (SV) index
Spearman's r = -0.04
corrected p = 0.0002
Spearman's r = -0.09
corrected p = 0.03
Spearman's r = 0.16
corrected p = 0.45
C
Spearman's correlation, # SSVs versus overall methylation
total SSVs
<1kb SSVs
1kb-10kb SSVs
10kb-100kb SSVs
100kb-1Mb SSVs
>1Mb SSVs
inversion SSVs
deletion SSVs
tandem duplic. SSVs
inter-chr transloc. SSVs
intra-chr transloc. SSVs
insertion SSVs
D
Overall methylation (median beta)
Spearman's r = -0.20
corrected p = 0.0002
Log2 total # SSVs - low pass cases (n=859)
Spearman's r = -0.12
corrected p = 0.01
Log2 total # SSVs - high pass cases (n=623)
E
Censored
bottom third SV index
middle third SV index
top third SV index
Survival probability
Log rank p <1E-25
corrected p = 0.16
Overall survival (months)
F
1482 cases ranked from high to low genomic rearrangments
high # SSV
low # SSV
top quartile
upper middle
lower middle
bottom quartile
low pass
cancer type
bladder, breast, cervix, cns, colorectal, head/neck, kidney, liver-biliary, lung, lymphoma, prostate, sarcoma, skin, stomach/eosophagus, thyroid, uterus
overall meth.
DNA damage signature
leukocyte fraction
LCK
SYK
mutation rate
SNV neoantigens
Indel neoantigens
lower (<0.3)
higher
lower
n = 1482 cases with DNA methylation data represented
correlation with total # SSVs: * corrected p<0.0005 ** corrected p<5E-8
G
patient age
Spearman's r = 0.14
corrected p = 0.0006
H
SOX17 DNA methylation (beta)
Log2 SOX17 mRNA expression
cg03329976
cg15710198
cg24928391
cg02919422
cg15377283
cg16830930
cg02222728
cg12883523
SOX17 mRNA

Fig. S8, related to Fig. 6. Additional information regarding global molecular alterations associated with the overall burden of structural variation across cancers. (A) Scatter plots of total number of SSV events versus overall methylation (median beta of all 450K probes within the sample profile), with samples that have only low pass WGS data being plotted separately from samples with high pass WGS data. P-values by linear model correcting for cancer type. **(B)** Scatter plots of global SSV index (measuring total number of SSV events, correcting for high pass versus low pass WGS) versus overall methylation (median beta of all of the given set of CGI probes within the sample profile), with overall methylation separately considered for all IlluminaCGI probes (left), for promoter-associated CGI probes (middle), and for gene body-associated CGI probes (right). P-values by linear model correcting for cancer type. **(C)** Spearman's correlations between overall structural variation burden and global decrease in overall methylation (related to Fig. 6C), when breaking down the SSVs according to class and size (size excluding translocation SSVs). **(D)** Association of global SSV index (measuring total number of SSV events, correcting for high pass versus low pass WGS) with patient survival. Corrected p-value is from stratified log-rank test according to cancer type. **(E)** Across the 1482 cases with DNA methylation data, with cases ranked high to low by global SSV index quartiles, selected molecular features are represented, including scoring for a transcriptional signature of DNA damage response pathway [9], a DNA methylation-based signature of lymphocytic infiltration [10], mRNA markers for T-cells and B-cells (*LCK* and *SYK*, respectively), exome mutation rate, and number of neoantigens (SNV, single nucleotide variant; Indel, insertion/deletion). P-values by linear model correcting for both cancer type and low pass versus high pass WGS. **(D)** Patient age corresponding to the cases in part C. P-values by linear model correcting for both cancer type and low pass versus high pass WGS. **(E)** Gene expression and DNA methylation associated with *SOX17*, corresponding to the cases in part C, as an example of a gene with alterations associated with the overall structural variation burden.

References

1. Johnson W, Rabinovic A, Li C: **Adjusting batch effects in microarray expression data using Empirical Bayes methods.** *Biostatistics* 2007, **8:**118-127.
2. Hu X, Wang Q, Tang M, Barthel F, Amin S, Yoshihara K, Lang F, Martinez-Ledesma E, Lee S, Zheng S, Verhaak R: **TumorFusions: an integrative resource for cancer-associated transcript fusions.** *Nucleic Acids Res* 2018, **46:**D1144-D1149.
3. Forbes S, Beare D, Boutselakis H, Bamford S, Bindal N, Tate J, Cole C, Ward S, Dawson E, Ponting L, et al: **COSMIC: somatic cancer genetics at high-resolution.** *Nucleic Acids Res* 2017, **45:**D777-D783.
4. Lawrence M, Stojanov P, Mermel C, Robinson J, Garraway L, Golub T, Meyerson M, Gabriel S, Lander E, Getz G: **Discovery and saturation analysis of cancer genes across 21 tumour types.** *Nature* 2014, **505:**495-501.
5. Chen F, Zhang Y, Gibbons D, Deneen B, Kwiatkowski D, Ittmann M, Creighton C: **Pan-cancer molecular classes transcending tumor lineage across 32 cancer types, multiple data platforms, and over 10,000 cases.** *Clin Cancer Res* 2018, **24:**2182-2193.
6. Peifer M, Hertwig F, Roels F, Dreidax D, Gartlgruber M, Menon R, Krämer A, Roncaioli J, Sand F, Heuckmann J, et al: **Telomerase activation by genomic rearrangements in high-risk neuroblastoma.** *Nature* 2015, **526:**700-704.
7. Lewis K, Tollefsbol T: **Regulation of the Telomerase Reverse Transcriptase Subunit through Epigenetic Mechanisms.** *Front Genet* 2016, **7:**83.
8. Whitfield ML, Sherlock G, Saldanha AJ, Murray JI, Ball CA, Alexander KE, Matese JC, Perou CM, Hurt MM, Brown PO, Botstein D: **Identification of genes periodically expressed in the human cell cycle and their expression in tumors.** *Mol Biol Cell* 2002, **13:**1977-2000.
9. Knijnenburg T, Wang L, Zimmermann M, Chambwe N, Gao G, Cherniack A, Fan H, Shen H, Way G, Greene C, et al: **Genomic and Molecular Landscape of DNA Damage Repair Deficiency across The Cancer Genome Atlas.** *Cell Rep* 2018, **23:**239-254.e236.
10. Thorsson V, Gibbs D, Brown S, Wolf D, Bortone D, Ou Yang T, Porta-Pardo E, Gao G, Plaisier C, Eddy J, et al: **The Immune Landscape of Cancer.** *Immunity* 2018, **48:**812-830.
