# Supplementary material for: Global impact of somatic structural variation on the DNA methylome of human cancers
Source: Genome Biol. 2019 Oct 15;20:209. doi: 10.1186/s13059-019-1818-9 (PMC6792267; doi:10.1186/s13059-019-1818-9)
Supplement: Supplementary file 12 — Additional file 12. Review history. [file 13059_2019_1818_MOESM12_ESM.docx]

Review History

**First round of review**

**Reviewer 1**

**Are you able to assess all statistics in the manuscript, including the appropriateness of statistical tests used?**

No, I do not feel adequately qualified to assess the statistics.

**Comments to author:**

Review manuscript Genome Biology GBIO-D-19-00385 Creighton lab

This study is in principle a very worthy undertaking, asking important questions and using a very impressive collection of data to get answers to these questions. Changes in genome structure, DNA methylation patterns and gene expression levels are each known to be hallmarks of cancer, so it is entirely plausible, and in many ways pressing, to investigate whether and in which way these three categories of molecular changes are associated. Starting with an analysis from '10,000 meters' first, by looking at patterns of association across the molecular categories, in a very large and impressive data set of >1400 cancer genomes, is an entirely plausible point of entry.

The study, as presented, then seems to suffer considerably from one conceptual choice, namely that all classes and sizes of SVs are lumped together, and that no distinction is made between genomic regions within and outside the boundaries of a given SV. It may well still be possible to sort things apart along these lines (i.e. by SV type and size and differentiating between regions within and outside of SVs), the required information to do so would be contained in the raw data that was used and maybe even in the intermediate processing steps. But whether it is something that will take a lot to do, or just a little, it needs to be addressed before one can fully assess the merits of this study.

Using the case of a deletion CNV here as an example to illustrate this: take a heterozygous deletion CNV, of 100,000 bp in size. It will have two breakpoints, and the 100kbp region between these two breakpoints exists now in only one copy. If there is a gene, and a CGI, within that 1-copy region, it should be treated differently in the analysis than, say, a gene or CGI right outside the breakpoints of this CNV, and different again than a gene and/or CGI outside and far away from this CNV. 
The reason is that for a gene or CGI within the CNV boundaries a drop in expression or methylation levels by around 50% would be the expected outcome - but it would actually mean that for this gene or CGI there was no compensation for the copy number change, therefore the remaining gene is simply expressing at its pre-deletion levels and the remaining CGI is still methylated at its pre-deletion levels. 
But for the gene or CGI outside the CNV boundaries, a drop of 50% in the expression or methylation levels would be a potentially important observation, and if it could be shown that such a 50% drop occurs more often for genes/CGI outside, but nearby, deletion CNVs then this would be an interesting and potentially very important observation. 
The same thinking in principle, just with reversed directionality, applies to duplication CNVs, and for copy-number neutral SV such as inversions yet again a different set of scenarios should be considered. 
This is meant to illustrate why it is essential for this type of analysis to break down SVs by type (deletion, duplication, inversion etc.), and ideally also by size class, and to analyze genomic regions that are within SV boundaries separately from regions outside SV boundaries (again, for the latter, with a distinction between SV-proximal and SV-distal outside SV-regions).

From what we understand, this is not what is presented in this manuscript. Rather, the authors treated each SV breakpoint as an individual and separate entity, whether it belongs to a deletion, duplication, inversion or other type of SV, and also did not consider whether a genomic region is within or outside of an SV. 
And there is no breakdown provided regarding the classes and size distributions (per class) of SVs. 
The information required to generate these breakdowns should be already present in the data that was used for this study, but it should be brought out and be made part of the analysis and presentation of results. As it is the results are rather difficult to interpret and no conclusion can be drawn yet about whether "higher or lower methylation was found to contribute to SV-associated CGI methylation alterations" as the authors stated.  
It is also premature to suggest a mechanistic link between SVs and DNA methylation, as it is premature to draw the conclusions that "Genomic rearrangement would represent an under-appreciated mechanism in shaping the cancer methylome" and "Tumor cells with high genomic rearrangement burden may have to overcome or evade the immune response".  

The authors claim that "to date there has been no global survey to identify CGI with altered methylation associated with nearby SV breakpoints."  This does not seem to be entirely correct.  There is one study published in Nucleic Acids Research in April of 2018 that performed association study between somatic copy number changes (a class of somatic structural variation) with DNA methylation and gene expression in tumor samples (PMID:  29529299), apparently using at least a subset of the same data that is used in the present study.  In that study, the authors found that somatic copy number changes affect DNA methylation and gene expression independently.  This is not what the authors found here.  The authors should cite and discuss this other study's results in relationship to theirs.  What might be happening?

One characteristic of the manuscript is that there are quite a few sentences and passages that are confusing at first and require to be read multiple times. This would ideally be fixed. 

Other than that, to recapitulate and give a few more details:

1.      It seems that the authors did not consider the heterogeneous nature of somatic SV events.  There are many SV types including deletions, duplications, inversions, insertions, and translocations.  These events occur at vastly different frequencies and size, ranging from hundreds to millions of base-pairs.  It is not clear what classes or fraction of each class and what size ranges of SVs the authors analyzed.  For instance, it is difficult to imagine, especially if the aim is to gain possible mechanistic insight, that deletions and duplications (although both are genomic rearrangements with breakpoints) should be grouped together for analysis. 

2.     Do the observed trends still exist if SVs are separated by type and size (small, medium, and large)? Are large SVs weighted the same as smaller ones in the analysis?  What about different window sizes outside of the SVs?

3.     The study essentially stands and falls with the accuracy and validity of somatic SVs called.  The authors claim that the Meerkat algorithm used has a >90% accuracy, which would be exceptionally good for an algorithm that uses split-reads and paired-ends but not read-depth (?).  It seems like this value is derived from a handful of validations from a very confident callset.  The authors should thoroughly investigate the false positive rate of their SV calling by class and size and take it into account in their regression models.  Do any somatic SVs actually overlap those in DGV (i.e. many SV calls from the 1000 Genomes project), which would mean that they are germline and incorrectly called as somatic? 
Also, for the genomes where SV calling had already been carried out by others, which analytical principles where used? Meerkat seems to be making calls using split-reads and paired-ends. If for the other sets of calls read-depth analysis was used, that would need to be noted and CNVs coming out of read-depth analysis might have to be treated differently from those coming out of split-read or paired-end analyses.

5.     The authors seem to show that there is a general association between SVs and DNA methylation from their large and impressive datasets, but this association and relationship should be analyzed more in depth particularly looking at SV classes and size into account.  Given the power of the dataset, this should really be looked at more in-depth.

6.      The authors sometimes use an FDR of <5% and sometimes FDR of <10%.  What is the justification?
 
7.     "Translocation of genomic regions normally having higher or lower methylation was found to contribute to SV-associated CGI methylation alterations."  (Also, translocation here is used to describe genomic rearrangement in general, which is not the correct usage).  How do you know that it is contributing when the two variables are simply associated (this is also an example for a confusingly written sentence)?

8.     "Across cancers, the overall structural variation burden was associated with global decrease in CGI methylation, increased expression in methyltransferase genes…"  This would be counterintuitive finding and would merit some additional discussion (if it still holds after the analysis has been done separately on different SV types and size classes).

9.  Why would "many more genes" be positively corrected with SV events considering the generally disruptive nature of SVs (page 6, line 18; page 10 line 13)

10.  from the Discussion, the  first sentence:  "This study provides a more comprehensive catalogue of mRNAs deregulated by nearby somatic genomic rearrangements".  Again this should be revisited after analysis done separately for SV classes/sizes and genes within or outside SV boundaries.

 
Additional minor comments:

DNA methylation also includes 5' hydroxylmethylcytosine (page 4, line 4)

Page 5 line 19, "here to harmonize the two datasets", what does harmonize mean specifically?

Page 11 line 44 "CGI probes were found consistently altered in expression…", what does expression here mean?

Page 11 line 51 "More CGI features were positively correlated", what does CGI features positively correlated mean?

Page 19, line 54:  "Overall structural variation burden (i.e. high number of detected SVs) was also associated … parallels in other contexts from previous studies."  Which previous studies?  What is the "high number"?

**Reviewer 2**

**Are you able to assess all statistics in the manuscript, including the appropriateness of statistical tests used?**

Yes, and I have assessed the statistics in my report.

**Comments to author:**

The paper "Global impact of somatic structural variation on the DNA methylome of human cancers" by Chad Creighton and colleagues presents a pan-cancer correlative analysis of somatic SVs with both gene expression and DNA methylation (DNAm). In the former case, it presents an updated analysis using a larger number of samples from both TCGA and PCAWG, whereas for the DNAm case this is an entirely novel analysis not attempted before, although methodologically the authors use effectively the same statistical framework for DNAm as they do for expression. The study is very comprehensive, somewhat lengthy though and hard to follow, and overall of great interest to the scientific community. The authors find many SVs that do correlate with nearby CGI methylation levels, in some cases targeting specific oncogenes and tumor suppressors where expression is also correspondingly altered. An interesting novel insight is the observation that the methylation level of translocated regions appears to correlate with the methylation of the CGI at the other side of the breakpoint, although as I argue below this analysis was perhaps done in the most sensible way, which means that results hard to interpret and their biological significance hard to ascertain. Overall SV burden is also correlated with measures of global CGI methylation, tumor purity and immune cell infiltration, but unfortunately CGI probes were not stratified according to promoter vs gene-body, in my opinion, a major flaw. The authors also study the different classes of SVs in relation to TADs, which leads to some novel biological insight, such as SVs being associated with overexpression being generally TAD-disruptive. In general, although the paper conducts some complicated analyses, it is actually very descriptive, and the whole motivation/rationale for some of the analyses performed is somewhat unclear. There are also some other specific conceptual weaknesses which make this paper somewhat hard to interpret. I think the authors need to address the major concerns listed below, to ensure that the ensuing interpretations are correct, and also to establish the overall biological significance of the findings:
Major concerns:
1) Justification for linear distance metric model is not provided: In the gene expression case the authors conduct an analysis based on specific genomic regions, as well as one based on using the distance metric, ie. the distance between the SV and the start of the gene. The authors seem to advocate the latter as it gives the authors more power to detect associations, as a region-based analysis may not contain enough SV events, specially when performing the cancer-specific analyses. However, the power-issue can be resolved by other means, for instance by simply aggregating the SV events over all gene regions and then binarizing the count, i.e. if a SV occurs in any of the gene regions up to +/-1Mb than consider it as 1, otherwise as 0. This approach has the same power, but also has another advantage, which is that it does not assume a linear dependence between the distance and expression (or methylation). Indeed, this brings me to the big problem I have with many of the analyses performed, which is that the authors assume, without proof, that a linear model is justified….I did not see the data to justify the use of a linear model. And how about the type of SV, would this also not be a more important factor than the distance? Or if we assume a monotonic function, why should it be linear as opposed to an exponential? I agree that an advantage of using a linear model is that it allows in principle easy integration of covariates, but problem is that if the underlying linear model is not a good one, then the output from the multivariate analysis is also not going to be very meaningful and potentially misleading.
2) SV - expression analysis ignores normal-tissue reference:  I think that a key issue that has been largely ignored (not just in this study but in many others), is that performing these correlations between SVs and gene expression across cancers may in fact identify many SVs where the aberrant expression is not really an event that is being selected for, i.e. did the authors check whether tumors not harboring the given SV exhibit higher/lower expression relative to the corresponding normal tissue? For instance, they may find a positive correlation between a SV and gene expression in one cancer-type, but maybe tumors of the same type not harboring the SV still exhibit much higher expression compared to the corresponding normal tissue, in which case the overexpression has nothing really to do with the SV event. 
3) Fig.3D:  It was unclear to me why the average DNAm difference for the CGI probes was done by comparing one cancer-type to all other cancer-types as opposed to comparing the cancer to the corresponding normal. Why this matters, is because a significant proportion of DNA methylation changes in cancer is the result of cell proliferation, and so comparing across different cancer-types is going to confound interpretation in relation to how proliferative or less proliferative the given cancer is.
4) Promoter vs gene-body CGI methylation: One of the biggest problems with this paper is that the authors don't stratify the CGI probes according to whether they are promoter or gene-body 3'UTR related, which makes the interpretation of most of the findings somewhat unclear. Even when they do make a distinction such as in SuppFig.S3C, the figure itself does not make it clear where the CPGs map to in relation to the gene. Or for instance, in Fig.3D, how many of the CGI probes being shown are gene-body related, how many are promoter related? 
5) Fig.5C: Fig.5C is an important panel in this paper, and yet here again the authors choose to do the analysis in a way which is perhaps not the most sensible one: in the barplot, the y-axis shows the average difference in DNAm between the rearranged region vs unaltered region based on normal-adjacent tissue. However, the x-axis refers to SV-CGIs groups that were deemed to be of higher or lower methylation by comparing the tumors with the SV to tumors without the SV, regardless of tissue-type. Would it not be more sensible to compare methylation of SV+ tumors within a given tumor type to the normal-adjacent tissue to define the categories on the x-axis? It would certainly make the data more interpretable, and I think it is also the more meaningful comparison?
6) Cell-type heterogeneity: the authors do adjust for tumor purity throughout their analysis, but when generating the data for Fig.5C, they also seem to ignore the fact that some of the loci could be cell-type specific markers (say marking immune cells), which could then confound the categorization of the SV-CGI groups on the x-axis i.e. differences in methylation between SV+ and SV- tumors could be due to varying amounts of immune cells.
7) Pan-cancer analysis vs cancer-specific analysis: It would appear that in the expression analysis at least, that most of the cancer-specific associations are indeed not shared with other cancer-types. In the pan-cancer analysis, I worry that some of the associations which were significant in the pan-cancer analysis are perhaps driven by the larger number of cancer samples for one given cancer-type. So, some of the pan-cancer hits may not really be pan-cancer or only appear so driven by the larger number of samples in one cancer-type: whilst an association in another cancer-type, say if seen for 2 samples of this cancer-type may appear to justify the pan-cancer status, statistically this may be the incorrect interpretation….
8) Confounding effect of cell-proliferation on DNAm:  the whole manuscript ignores the effect of cell proliferation on DNA methylation, which is significant and a major reason why the aberrant DNAm landscape across cancer-types is so similar. This, together with the fact that CGI probes were not stratified according to gene region, makes some of the results hard to interpret. For instance, overall SV burden was associated with lower overall DNAm, but treating all CGI probes equally does not allow for this to be correctly interpreted. I would like to see all analyses performed depending on where the CGI probe maps to, so e.g. for Fig.6C we should display this for CGI promoter and CGI gene body probes separately.


Minor points:
-Adjustment for CNA: When adjusting for CNAs it is not clear how the CN-state of the gene was defined, since the copy-number state refers to a genomic segment, and the correlation analysis is done between expression and distance to BP. So for instance, was a CN- gain including the regulatory element of the gene required for a gene to be assigned the status of 1-copy gain? Approximations/errors like these could easily lead to apparent "independence" in multivariate regression, but only because the CNA covariate is subject to error. 
- Correction for CN change when inferring DNAm: I wonder if the authors considered that measured DNAm levels could also be confounded/affected by the underlying CN-state in which the CGI probes are found. Could the authors please comment? See e.g. Zhou W, Triche TJ Jr, Laird PW, Shen H. Nucleic Acids Res. 2018 Nov 16;46(20):e123.

**Authors Response**

**Point-by-point responses to the reviewers’ comments:**

Reviewer #1 comments:

This study is in principle a very worthy undertaking, asking important questions and using a very impressive collection of data to get answers to these questions. Changes in genome structure, DNA methylation patterns and gene expression levels are each known to be hallmarks of cancer, so it is entirely plausible, and in many ways pressing, to investigate whether and in which way these three categories of molecular changes are associated. Starting with an analysis from '10,000 meters' first, by looking at patterns of association across the molecular categories, in a very large and impressive data set of >1400 cancer genomes, is an entirely plausible point of entry.

*We thank the reviewer for evaluating our work.*

The study, as presented, then seems to suffer considerably from one conceptual choice, namely that all classes and sizes of SVs are lumped together, and that no distinction is made between genomic regions within and outside the boundaries of a given SV. It may well still be possible to sort things apart along these lines (i.e. by SV type and size and differentiating between regions within and outside of SVs), the required information to do so would be contained in the raw data that was used and maybe even in the intermediate processing steps. But whether it is something that will take a lot to do, or just a little, it needs to be addressed before one can fully assess the merits of this study.

Using the case of a deletion CNV here as an example to illustrate this: take a heterozygous deletion CNV, of 100,000 bp in size. It will have two breakpoints, and the 100kbp region between these two breakpoints exists now in only one copy. If there is a gene, and a CGI, within that 1-copy region, it should be treated differently in the analysis than, say, a gene or CGI right outside the breakpoints of this CNV, and different again than a gene and/or CGI outside and far away from this CNV.

The reason is that for a gene or CGI within the CNV boundaries a drop in expression or methylation levels by around 50% would be the expected outcome - but it would actually mean that for this gene or CGI there was no compensation for the copy number change, therefore the remaining gene is simply expressing at its pre-deletion levels and the remaining CGI is still methylated at its pre-deletion levels.

But for the gene or CGI outside the CNV boundaries, a drop of 50% in the expression or methylation levels would be a potentially important observation, and if it could be shown that such a 50% drop occurs more often for genes/CGI outside, but nearby, deletion CNVs then this would be an interesting and potentially very important observation.

The same thinking in principle, just with reversed directionality, applies to duplication CNVs, and for copy-number neutral SV such as inversions yet again a different set of scenarios should be considered. This is meant to illustrate why it is essential for this type of analysis to break down SVs by type (deletion, duplication, inversion etc.), and ideally also by size class, and to analyze genomic regions that are within SV boundaries separately from regions outside SV boundaries (again, for the latter, with a distinction between SV-proximal and SV-distal outside SV-regions).

From what we understand, this is not what is presented in this manuscript. Rather, the authors treated each SV breakpoint as an individual and separate entity, whether it belongs to a deletion, duplication, inversion or other type of SV, and also did not consider whether a genomic region is within or outside of an SV.

And there is no breakdown provided regarding the classes and size distributions (per class) of SVs.

The information required to generate these breakdowns should be already present in the data that was used for this study, but it should be brought out and be made part of the analysis and presentation of results.

*By design, our analytical approach does not assume the specific mechanism of altered expression or methylation, and this would include treating SV breakpoints representing different classes and insert sizes the same. Throughout this rebuttal and in the revised manuscript, we do explore differences involving SV class and size, as well as the impact of copy number alterations (CNA).*

*This present study represents our third study utilizing the same basic approach integrating SV data with gene expression data, while the present study is unique in focusing on DNA methylation. In collaboration with the Pan-Cancer Analysis of Whole Genomes (PCAWG) consortium, we carried out a previous study integrating SVs with gene expression, for just the PCAWG cohort, which manuscript was originally uploaded to bioRxiv in 2017 (https://doi.org/10.1101/099861) and is now accepted in principle at Nature Communications (accepted July 2018, to be published in conjunction with the other PCAWG consortium papers now under revision or resubmission to Nature). Another study of ours, published in July 2018 in Cell Reports (https://www.cell.com/cell-reports/fulltext/S2211-1247(18)30920-3), analyzed a different cohort from that of PCAWG, in collaboration with members of the TCGA Genome Characterization Center (GCC) for Whole Genome Sequencing (WGS) analysis, which members were named authors of that study as well as of this third study.*

*By analyzing the set of SVs associated with gene deregulation, we can identify overall trends representing associated mechanisms, e.g. involving TAD disruption or enhancer hijacking. However, for any given gene, there may be no single mechanism affecting all involved cases, for us to confidently explain the observed de-regulated expression patterns in every case; different cancer cases may have different types of alterations that achieve the same result. In addition, there are limitations with using WGS data to infer mechanisms of SV-mediated deregulation, as SV breakpoint data primarily provides only genomic coordinates and whether the corresponding upstream or downstream sequence at each breakpoint would be involved at the breakpoint junction. Multiple breakpoints may be involved within a given genomic region, not all of which may be identifiable within a WGS profile, making it difficult for one to trace the precise upstream or downstream sequence near a gene resulting from a given SV breakpoint. Also, in many cases, the orientation of the SV breakpoint may not allow us to conjecture what new sequences are being positioned directly adjacent to the gene. Nevertheless, as our analysis approaches to identifying SV-associated molecular alterations do not assume a particular mechanism, we were able to identify recurrent patterns regardless of the mechanisms involved.*

*The nature of the difficulty of assuming a mechanism of SV-mediated altered expression or methylation, is illustrated by the figure below (taken from Figure 6B of our previously-published Cell Reports paper), which depicts the TERT locus and associated TADs and SVs. The TERT region is peppered with SV breakpoints, and a given case may have multiple adjacent breakpoints nearby the gene. Most any gene of interest would be similarly complex. One cannot state definitely what the mechanism would be for a given sample with altered TERT expression. The class of the nearby SV would not necessarily reveal the mechanism. For example, a deletion SV may remove a small repressive regulatory element near the gene, causing the gene to be over-expressed; in another case, a tandem duplication SV may amplify the region surrounding a gene, but in the process breaks a promoter element, causing the gene itself to not be expressed; in other cases, there may be an SV breakpoint that is closest to the gene but which is having no effect, but then another SV right next to the first SV is actually the one that is driving the gene (e.g. a translocation SV bringing an enhancer element in proximity). Any number of scenarios may be envisioned. The regulatory landscape for any given gene in most cases has not been fully elucidated. An analytical approach that assumes too much in terms of mechanistic effects is likely to be both overly complicated and wrong in most instances.*

*Regarding the Reviewer’s concerns involving CNA (i.e. CNV), we should note that the gene-level CNA was corrected for, in each of the mRNA or CGI feature sets put forth for downstream analyses. Models that did not correct for CNA were considered in our previous studies as well as in Figure 2C (shown below) of the present study. The observed widespread impact of somatic SVs on CNAs (notably with nearby breakpoints involving copy gain) has been reported previously and is further illustrated in our Figure 2C. With a linear model relating SVs to gene expression, without factoring in CNA (second group from the right), we find thousands of genes statistically significant for each region. However, when we incorporate CNA into the model (group on the right), most genes significant in the previous models are now not significant, meaning that CNA alone is able to explain the increased expression that might be associated with SVs. It is the set of genes represented in the last group below that we carry forward into the subsequent analyses represented in the other figures. We would agree that when describing the global impact of SVs on gene expression, it becomes important to document which associations might be attributable to corresponding amplification of the gene itself versus those that might be attributable to something else. By our models, it would be possible, for example, for a gene not to be amplified but for the region adjacent to the gene containing an enhancer to be amplified (e.g. by tandem duplication SV), thereby increasing gene expression. In other cases, the gene may be amplified but the amplification levels are unable to fully account for the SV-associated increase in expression. Both of the above scenarios would be of interest to us for inclusion in the final results.*

*While SVs may be balanced or unbalanced in terms of CNA within the immediate vicinity of the breakpoint (e.g. involving deletions, insertions, or tandem duplications), this may not reflect the copy levels of the nearby gene. For example, a tandem duplication event near a gene may or may not be associated with amplification of the gene itself (if it is, our analytical approach will adjust for the genelevel CNA). In our previous Cell Reports and PCAWG papers, we had observed broad associations involving SVs with increased copy number, which was consistent with similar observations made in other studies outside of our group (Weischenfeldt et al., 2013, PMID: 27869826; Yang et al., 2016, PMID: 27153396). We can keep in mind that an association in and of itself would not necessarily be causal; so, for example, if a specific deletion SV event is associated with copy gain of the nearby region, we understand the SV itself represents the deletion of the specific region defined by the SV, but that a number of other events can explain an observed gene-level or cytoband-level gain, for example the fact that breakpoints from many different SVs can occur within the same genomic region. As shown in the figures below (taken from Figure 1 of our Cell Reports paper), SV breakpoints associated with copy gain were significantly enriched for breakpoints involving inversion SVs or deletion with insertion SVs, while SV breakpoints associated with copy loss were significantly enriched for breakpoints involving deletion SVs; however, all classes of SVs could be associated with CNA.*

As it is the results are rather difficult to interpret and no conclusion can be drawn yet about whether "higher or lower methylation was found to contribute to SV-associated CGI methylation alterations" as the authors stated.

*The full statement in question from the Abstract is as follows: “Translocation of genomic regions normally having higher or lower methylation was found to contribute to SV-associated CGI methylation alterations.” We have edited this statement to read as follows: “Rearrangement of genomic regions normally having higher or lower methylation was involved with SV-associated CGI methylation alterations.” (in order not to assume a direct mechanistic link in every observed instance). This statement specifically refers to results presented Figure 6C and 6D.*

It is also premature to suggest a mechanistic link between SVs and DNA methylation, as it is premature to draw the conclusions that "Genomic rearrangement would represent an under-appreciated mechanism in shaping the cancer methylome" and "Tumor cells with high genomic rearrangement burden may have to overcome or evade the immune response".

*We have edited the abstract with regards to these concerns. Specifically, we have removed the statement “Tumor cells with high genomic rearrangement burden may have to overcome or evade the immune response” (though it represents a reasonable interpretation of some of our results, even if our study did not directly demonstrate this) and we have edited the other statement in question to read as follows: “Genomic rearrangement would appear to have a major role in shaping the cancer methylome.” It is already understood, for example, that DNA repair of double stranded breaks—which would be involved in genomic rearrangements—can lead to altered CpG methylation at the repair site, with corresponding changes in expression of the genes being associated with the repaired region (as described in refs 9-11 of our manuscript). In the Discussion section and elsewhere, we have similarly qualified statements related to the above.*

The authors claim that "to date there has been no global survey to identify CGI with altered methylation associated with nearby SV breakpoints." This does not seem to be entirely correct. There is one study published in Nucleic Acids Research in April of 2018 that performed association study between somatic copy number changes (a class of somatic structural variation) with DNA methylation and gene expression in tumor samples (PMID: 29529299), apparently using at least a subset of the same data that is used in the present study. In that study, the authors found that somatic copy number changes affect DNA methylation and gene expression independently. This is not what the authors found here. The authors should cite and discuss this other study's results in relationship to theirs. What might be happening?

*We have cited the above paper in the manuscript revision. As noted above and as more emphasized in the revised manuscript, we explicitly corrected for CNA in each of the CGI feature sets put forth for downstream analyses. So, the study we carried out would be looking at a very different phenomenon from the NAR paper. We would not be examining how somatic CNA affects DNA methylation, but rather how SVs affect methylation to a degree that would not be explainable by any associated CNA.*

One characteristic of the manuscript is that there are quite a few sentences and passages that are confusing at first and require to be read multiple times. This would ideally be fixed.

*We appreciate the Reviewer’s patience. For this revision, we have carefully worked through the manuscript text to address possible points of confusion, e.g. as indicated by some of the Reviewer’s concerns.*

Other than that, to recapitulate and give a few more details:

1. It seems that the authors did not consider the heterogeneous nature of somatic SV events. There are many SV types including deletions, duplications, inversions, insertions, and translocations. These events occur at vastly different frequencies and size, ranging from hundreds to millions of base-pairs. It is not clear what classes or fraction of each class and what size ranges of SVs the authors analyzed. For instance, it is difficult to imagine, especially if the aim is to gain possible mechanistic insight, that deletions and duplications (although both are genomic rearrangements with breakpoints) should be grouped together for analysis.

*In the revised manuscript, we explore differences involving SV class and size involving the SV-associated gene feature alterations. As we discuss above, our analytical approach by design does not assume the specific mechanism of altered expression or methylation. In the downstream analyses, we can examine the top features for enrichment patterns, which can lead to some interesting findings. In principle, all classes and sizes of SVs could conceivably lead to altered expression or methylation of nearby genes, in either direction, so we want to keep an open mind in defining top features but then as a next step we can see whether the results appear enriched for particular classes or sizes. The new Figures S3D and S3E, added to the revision and also shown below, provide breakdowns by SV class and SV size, for both the SV breakpoints associated with gene over-expression or gene underexpression (FDR0.4SD or <-0.4SD, respectively, from median for the case harboring the breakpoint). Enrichment p-values by chi-square test are also provided, comparing the distribution of the given top features set with the expected distribution as based on the entire SV call set.*

*From the above, we see that all classes and sizes are well represented among the top significant SV breakpoint associations. There is a statistical enrichment for inter-chromosomal translocation SV represented among the breakpoints associated with gene over-expression, which makes sense given our observations elsewhere (Figure 5A) of TAD disrupting SVs (many of which are inter-chromosomal) being enriched for SVs associated with over-expression. There is also a slight enrichment of tandem duplication SVs within the over-expression-associated breakpoints and a slight enrichment for deletion SVs within the under-expression associated breakpoints, which makes sense though these still respectively represent only a fraction of the breakpoints involved.*

*For DNA methylation, the new Figures S6D and S6E, added to the revision and also shown below, provide breakdowns by SV class and SV size, for both the SV breakpoints associated with higher or lower CGI methylation (FDR0.4SD or <-0.4SD, respectively, from median for the case harboring the breakpoint). Again, we see that all classes and sizes are well represented among the top significant SV breakpoint associations. There is a statistical enrichment for inter-chromosomal translocation SV represented among the breakpoints associated with either increased or decreased methylation, which again makes sense given our other observations involving TAD disrupting SVs. Some enrichment patterns according to size can be observed and provide some information, though these patterns respectively represent only a fraction of the breakpoints involved.*

*In addition, in the new Supplementary Figures S2 and S5 (also shown below under Reviewer 2’s comment #2), we have taken the top set of genes and CGIs associated with altered expression or methylation, respectively, and annotated these molecular features according to SV class and size representation.*

2. Do the observed trends still exist if SVs are separated by type and size (small, medium, and large)? Are large SVs weighted the same as smaller ones in the analysis? What about different window sizes outside of the SVs?

*As described above, the observed trends involved all SV sizes, even if some patterns of relative enrichment may be noted. As explained above, large SVs would be weighted the same as smaller ones. In theory, a small SV deletion could take out a repressive element, where another large SV deletion might be positioned such that it does not affect the regulation of a particular gene. If an SV spans a gene and results in CNA of that gene, the gene feature would be found significant only if the association expression or methylation could not be adequately explained by the CNA alone. By our models, it would be possible, for example, for a gene not to be amplified but for the region adjacent to the gene containing an enhancer to be amplified (e.g. by tandem duplication SV), thereby increasing gene expression, but this scenario would be of interest to us for inclusion in the final results.*

3. The study essentially stands and falls with the accuracy and validity of somatic SVs called. The authors claim that the Meerkat algorithm used has a >90% accuracy, which would be exceptionally good for an algorithm that uses split-reads and paired-ends but not read-depth (?). It seems like this value is derived from a handful of validations from a very confident callset. The authors should thoroughly investigate the false positive rate of their SV calling by class and size and take it into account in their regression models. Do any somatic SVs actually overlap those in DGV (i.e. many SV calls from the 1000 Genomes project), which would mean that they are germline and incorrectly called as somatic?

Also, for the genomes where SV calling had already been carried out by others, which analytical principles where used? Meerkat seems to be making calls using split-reads and paired-ends. If for the other sets of calls read-depth analysis was used, that would need to be noted and CNVs coming out of read-depth analysis might have to be treated differently from those coming out of split-read or paired-end analyses.

*At the Reviewer’s request, we checked the DGV germline call set, consisting of 392,583 SVs, against our somatic SV call set, consisting of nearly 270,000 SVs. Exactly four SVs from our set overlapped with SGV, and none of these four were actually involved in the top significant mRNAs or CGI methylation features by distance metric method (i.e. either the questionable SVs were not anywhere in proximity to a gene or CGI called significant, or, in the one case where an SVs was near a significant CGI, the associated sample was non-TCGA and did not have DNA methylation data and was therefore not used in the DNA methylation analysis). We have made note of this in the Methods of the revised manuscript. This result reflects the care that was initially taken in subtracting out any germline SVs when making somatic calls. The SV calls used in this study, as provided by PCAWG and by the TCGA WGS GCC (which team members are authors of this present study), represent the cumulative efforts of hundreds of researchers across multiple institutions over several years. There would be no viable path to our substantially improving upon these data. The TCGA GCC calls have been utilized effectively in previous TCGA consortium-led studies focusing on a specific cancer type (TCGA CRC, Nature 2012; TCGA KIRC, Nature 2013; TCGA HNSC, Nature 2015; TCGA SKCM, Cell 2015; TCGA Gastric, Nature 2014; TCGA BLCA, Nature 2014; TCGA PRAD, Cell 2015; TCGA CESC, Nature 2017; TCGA ESCA, Nature 2017; TCGA KICH, Cancer Cell 2014; TCGA UVM, Cancer Cell 2017). The PCAWG consortium main “theme” papers utilizing the SV data (currently uploaded on Biorxiv) are now under revision or resubmission to Nature. While our present study utilizes a high quality and carefully curated dataset, false positive and false negative calls remain an unavoidable factor that would be difficult for us to quantify; however, the integration between SV and gene expression or DNA methylation greatly limits the potential of our finding positive associations that are driven primarily by imperfections in the SV calling.*

*For PCAWG, SV calls were made by three different data centers using a number of algorithms: SvABA, BRASS, DELLY, and dRanger, all of which are well-established and widely used, although to our knowledge none of the four callers uses read depth. Calls made by at least two algorithms were used in the downstream analyses, along with additional filtering criteria being used as described by the PCAWG consortium (https://doi.org/10.1101/161638, https://doi.org/10.1101/187609). The fact that our previous Cell Reports and PCAWG studies observed the same overall phenomenon demonstrates that our findings would not be dependent upon the parameters of a particular SV calling pipeline. For this present study, all available SV calling information (both PCAWG calls and TCGA WGS GCC calls) was brought together, in the interests of the downstream integrative analyses with both gene expression and DNA methylation. True SV calls missed by one calling dataset would potentially supplemented by inclusion of results from another dataset. As one notable example, of the five cases of chromophobe renal cell carcinoma (KICH) for which an SV breakpoint upstream of TERT was both identified by Meerkat WGS analysis and independently confirmed by PCR (TCGA KICH paper, Cancer Cell 2014, 26:319-330), just three cases had the same SV breakpoints detected by PCAWG analysis, consistent with observations elsewhere that ensembles of SV analysis pipelines do not always outperform individual SV calling methods (Lee et al., Genome Biol 2018, 19:188). We would not be able to derive a set of general rules, e.g. based on our own past work or the work of PCAWG consortium, for weighting SVs differently in our models according to class or size, which would make for much more complicated models based on highly questionable assumptions.*

*The integration of results between orthogonal platforms (e.g. WGS and RNA-seq) is a key aspect of our study, as associations identified must be significant enough to rise above any noise. SV calling algorithms remain a developing area of research (Lee et al., Genome Biol 2018, 19:188), and no SV calling dataset, including ours, can be completely free of false positives and false negatives. However, a significant mRNA or CGI, as identified using our methods, must align non-randomly with an SV breakpoint pattern, making it much harder for false positive SV calls to drive a significant association. False negative SV calls can result from a number of factors including lack of read depth or strict filtering criteria, but missed calls would work against a significant association being found, resulting in false negatives rather than false positives. Results of a number of analyses in our study, including permutation testing results and model-based estimates, indicate that, despite the biological and technical noise involved in each of the data platforms, the vast majority of the significant gene features observed using the actual datasets would not be explainable by noise (whether technical or biological), chance, or multiple testing.*

5. The authors seem to show that there is a general association between SVs and DNA methylation from their large and impressive datasets, but this association and relationship should be analyzed more in depth particularly looking at SV classes and size into account. Given the power of the dataset, this should really be looked at more in-depth.

*In our response to the Reviewer’s point #1, we have examined SV classes and sizes with respect to the SV breakpoints associated with altered DNA methylation of specific CGIs (involving Figure 4 results). In addition, we have re-examined our reported relationship between overall structural variation burden and global decrease in overall methylation (involving Figure 6), when breaking down the SVs according to class and size. These results are presented in the new Supplementary Figure 8C (also shown below). Overall, we see the same basic trends for each SV subgroup.*

6. The authors sometimes use an FDR of <5% and sometimes FDR of <10%. What is the justification?

*In principle, both FDR cutoffs would be considered acceptable and are in common use (e.g. an FDR of <10% would indicate that on the order of ~90% of the nominally significant features should represent true positives). In our study, we would use FDR of 5% to define the top features as surveyed by various methods (e.g. looking at different genomic region windows), but in situations where fewer samples were involved (e.g. the cancer type-specific analyses of Figure 3) or where we examined the overlap between two results sets (e.g. the methylation versus expression results of Figure 4D) more relaxed statistical cutoffs (e.g. FDR<10%) were used. In the manuscript revision, we have added a note in Methods regarding the above.*

7. "Translocation of genomic regions normally having higher or lower methylation was found to contribute to SV-associated CGI methylation alterations." (Also, translocation here is used to describe genomic rearrangement in general, which is not the correct usage). How do you know that it is contributing when the two variables are simply associated (this is also an example for a confusingly written sentence)?

*The Reviewer’s points are well taken, as we have uncovered what appear to be global associations, but where we cannot necessarily with certainty whether any particular association involving a given sample and gene is truly a cause and effect relationship. We have revised the statement question in the Abstract to read as follows: “Rearrangement of genomic regions normally having higher or lower methylation was often involved in SV-associated CGI methylation alterations.” We have edited the related statements in the main text accordingly, and have used the term “rearrangement” rather than “translocation” as advised.*

8. "Across cancers, the overall structural variation burden was associated with global decrease in CGI methylation, increased expression in methyltransferase genes…" This would be counterintuitive finding and would merit some additional discussion (if it still holds after the analysis has been done separately on different SV types and size classes).

*As noted in Discussion section, regarding the observed increased expression in DNA Damage Response and of DNA methyltransferase genes with increasing structural variation burden, both of these perhaps represent a transcription program initiated without success to combat genomic instability. Elsewhere, genome-wide DNA hypomethylation despite an increase in DNA methyltransferase activity and gene-specific regional hypermethylation has been observed in cancer (refs 39 and 40). In general, the cancer transcriptome can reflect a set of programs being initiated by the cell, often in response to something, but these programs do not necessarily translate into a reversal of the global state of the cell. For example, it is entirely possible for the cancer cell to try and compensate for the lower overall methylation state by up-regulating methyltransferase genes, but these genes are apparently not able to keep up with the changes brought about by a high structural variation burden.*

9. Why would "many more genes" be positively corrected with SV events considering the generally disruptive nature of SVs (page 6, line 18; page 10 line 13)

*This present study represents our third study in which we have observed this phenomenon of more genes being positively correlated than negatively correlated in expression with nearby SV breakpoints, utilizing the same basic approach as before integrating SV data with gene expression data. Our previous two studies looking at separate cohorts made similar observations (one study in collaboration with the PCAWG consortium, manuscript uploaded to bioRxiv in 2017,* [*https://doi.org/10.1101/099861*](https://doi.org/10.1101/099861)*, and now accepted in principle at Nature Communications ; and the other study published in Cell Reports,* [*https://www.cell.com/cell-reports/fulltext/S2211-1247(18)30920-3*](https://www.cell.com/cell-reports/fulltext/S2211-1247(18)30920-3)*, in collaboration with members of the TCGA GCC WGS analysis team). This would represent an actual finding and the way we might understand it, is that genomic alterations that might happen at random versus alterations that would be selected for in the tumor are two separate things. Tumors will select for alterations that provide a survival benefit. This would be analogous to the types of genes we see globally impacted by CNA or by mutation. Algorithms like GISTIC or MutSig do not select for genes representing random events, but rather we see oncogenes and tumor suppressors being impacted in a consistent way. Similarly, our approach is not biased towards identifying over-expressed versus under-expressed genes, but the over-expressed genes include many well-known oncogenes and the under-expressed genes include well-known tumor suppressors, so the results overall make sense to us.*

10. from the Discussion, the first sentence: "This study provides a more comprehensive catalogue of mRNAs deregulated by nearby somatic genomic rearrangements". Again this should be revisited after analysis done separately for SV classes/sizes and genes within or outside SV boundaries.

*Our overall findings regarding SV-associated mRNA deregulation would be line with those of our two previous studies looking at the two separate cohorts (*[*https://doi.org/10.1101/099861*](https://doi.org/10.1101/099861) *and* [*https://www.cell.com/cell-reports/fulltext/S2211-1247(18)30920-3)*](https://www.cell.com/cell-reports/fulltext/S2211-1247(18)30920-3))*. However, we are able in the present study to make use of the combined dataset, where using the greater sample power and additional cancer types we could identify significant genes that were missed in the separate studies (Figure 1D). As might be expected in theory, the observed significant SV-gene feature associations involved all SV classes and sizes. Gene features impacted by SV-mediated CNA would be found significant only if the association expression or methylation could not be adequately explained by the CNA alone, and this CNA correction is carried out regardless of whether the SV boundaries span the gene or are outside of the gene.*

Additional minor comments:

DNA methylation also includes 5' hydroxylmethylcytosine (page 4, line 4)

*We have edited the statement in question to refer to “Classical DNA methylation” involving the classical epigenetic mark, 5-methylcytosine (Breiling and Lyko, Epigenetics Chromatin. 2015; 8: 24), versus other types of DNA methylation.*

Page 5 line 19, "here to harmonize the two datasets", what does harmonize mean specifically?

*Harmonize here refers to batch correction using Combat being carried out to account for different data platforms or processing methods being involved between PCAWG and TCGA. As seen in Supplementary Figure S1, hierarchical clustering of the RNA-seq datasets demonstrate that pre-Combat sample profiles segregate according to processing method while post-Combat sample profiles segregate according to cancer type, as desired.*

Page 11 line 44 "CGI probes were found consistently altered in expression…", what does expression here mean?

*This should read as “methylation” instead of “expression.” Thanks for catching that.*

Page 11 line 51 "More CGI features were positively correlated", what does CGI features positively correlated mean?

*“Positively correlated” here means showing increasing methylation with nearby breakpoint. We have noted this in the revision.*

Page 19, line 54: "Overall structural variation burden (i.e. high number of detected SVs) was also associated … parallels in other contexts from previous studies." Which previous studies? What is the "high number"?

*The high number of detected SVs would be relative to other cases, correcting for differences in coverage, as described in Methods. So, the overall SV burden would be essentially a relative measure, by which we compare cases with relatively high numbers of detected SVs with the other cases. The previous studies noted in this first sentence are described in the following sentences in this Discussion paragraph.*

Reviewer #2 comments:

The paper "Global impact of somatic structural variation on the DNA methylome of human cancers" by Chad Creighton and colleagues presents a pan-cancer correlative analysis of somatic SVs with both gene expression and DNA methylation (DNAm). In the former case, it presents an updated analysis using a larger number of samples from both TCGA and PCAWG, whereas for the DNAm case this is an entirely novel analysis not attempted before, although methodologically the authors use effectively the same statistical framework for DNAm as they do for expression.

The study is very comprehensive, somewhat lengthy though and hard to follow, and overall of great interest to the scientific community. The authors find many SVs that do correlate with nearby CGI methylation levels, in some cases targeting specific oncogenes and tumor suppressors where expression is also correspondingly altered.

*We thank the reviewer for evaluating our work and for patiently working through the manuscript to provide constructive comments.*

An interesting novel insight is the observation that the methylation level of translocated regions appears to correlate with the methylation of the CGI at the other side of the breakpoint, although as I argue below this analysis was perhaps done in the most sensible way, which means that results hard to interpret and their biological significance hard to ascertain.

*We address the related comments below under the Reviewer’s point #5.*

Overall SV burden is also correlated with measures of global CGI methylation, tumor purity and immune cell infiltration, but unfortunately CGI probes were not stratified according to promoter vs gene-body, in my opinion, a major flaw.

*We examine promoter versus gene body probes below under the Reviewer’s points #4 and #8.*

The authors also study the different classes of SVs in relation to TADs, which leads to some novel biological insight, such as SVs being associated with overexpression being generally TAD-disruptive. In general, although the paper conducts some complicated analyses, it is actually very descriptive, and the whole motivation/rationale for some of the analyses performed is somewhat unclear. There are also some other specific conceptual weaknesses which make this paper somewhat hard to interpret. I think the authors need to address the major concerns listed below, to ensure that the ensuing interpretations are correct, and also to establish the overall biological significance of the findings:

*Below we address the Reviewer’s major concerns. The goal of our present study was to survey DNA methylation patterns in the context of SVs in cancer. A systematic cataloguing of gene transcripts and CGIs impacted by genomic rearrangement, using multiple patient cohorts and cancer types, can provide insight into cancer-relevant alterations outside of exome boundaries. Our present study would be analogous to other pan-cancer genomics studies that have cataloged the set of recurrently and significantly mutated genes across human cancers (Chang et al., 2016; Gonzalez-Perez et al., 2013; Kandoth et al., 2013; Lawrence et al., 2014; Martincorena et al., 2017).*

Major concerns:

1) Justification for linear distance metric model is not provided: In the gene expression case the authors conduct an analysis based on specific genomic regions, as well as one based on using the distance metric, ie. the distance between the SV and the start of the gene. The authors seem to advocate the latter as it gives the authors more power to detect associations, as a region-based analysis may not contain enough SV events, specially when performing the cancer-specific analyses. However, the power-issue can be resolved by other means, for instance by simply aggregating the SV events over all gene regions and then binarizing the count, i.e. if a SV occurs in any of the gene regions up to +/-1Mb than consider it as 1, otherwise as 0. This approach has the same power, but also has another advantage, which is that it does not assume a linear dependence between the distance and expression (or methylation). Indeed, this brings me to the big problem I have with many of the analyses performed, which is that the authors assume, without proof, that a linear model is justified…..I did not see the data to justify the use of a linear model. And how about the type of SV, would this also not be a more important factor than the distance? Or if we assume a monotonic function, why should it be linear as opposed to an exponential? I agree that an advantage of using a linear model is that it allows in principle easy integration of covariates, but problem is that if the underlying linear model is not a good one, then the output from the multivariate analysis is also not going to be very meaningful and potentially misleading.

*This present study represents our third study utilizing the basic “genomic region windows” approach integrating SV data with gene expression data, using linear models. In collaboration with the Pan-Cancer Analysis of Whole Genomes (PCAWG) consortium, we carried out a previous study integrating SVs with gene expression, for just the PCAWG cohort, which manuscript was originally uploaded to bioRxiv in 2017 (*[*https://doi.org/10.1101/099861*](https://doi.org/10.1101/099861)*) and is now accepted in principle at Nature Communications (accepted July 2018, to be published in conjunction with the other PCAWG consortium papers now under revision or resubmission to Nature). Another study of ours, published in July 2018 in Cell Reports (*[*https://www.cell.com/cell-reports/fulltext/S2211-1247(18)30920-3)*](https://www.cell.com/cell-reports/fulltext/S2211-1247(18)30920-3))*, analyzed a different cohort from that of PCAWG, in collaboration with members of the TCGA Genome Characterization Center (GCC) for Whole Genome Sequencing (WGS) analysis.*

*In this present study, we developed a slightly different analytical approach, representing a variation of genomic region windows, to associate the expression of each gene with nearby SV breakpoints. This new approach, which we termed “distance metric,” appears to be the one for which the Reviewer has stated concerns, although the two different approaches are not all that different from each other. For the distance metric approach, we computed a “relative distance metric” for each sample and gene, which was the relative distance of the SV breakpoint closest to the gene start site (upstream or downstream). A data matrix of absolute relative distances for all 17998 genes and 2334 samples was assembled, with a relative distance metric of 1Mb being applied for any sample with no breakpoints within 1Mb of the gene. Using this breakpoint pattern matrix, the correlation between expression of each gene and the presence of nearby SV breakpoints could be assessed, using linear regression models that would allow for incorporation of any relevant covariates. One important detail concerning the linear modeling is that the relative distances are log-transformed to more closely align with model assumptions, something which we have made clearer in the revised text. Among other things, the distance metric method provides a single result for each gene across the samples, representing genes consistently altered across the entire +/- 1Mb region examined, avoiding the issue of multiple testing of a number of adjacent regions. At the same time, the distance metric method may not be the best fit for all genes, and so we provide results for both distance metric and genomic region windows.*

*In all of the linear models performed in our study, appropriate data transformations were used to make the data align better with the model assumptions. For example, gene expression data was log2-transformed, a ubiquitous practice for linear model-based methods for differential expression including SAM and limma. For DNA methylation beta values, the logit transformation was used, also a common practice for making DNA methylation data better align with linear model assumptions (Xie et al. Bioinformatics. 2019 35:1094-1097). Similarly, relative SV breakpoint distances from the gene in the breakpoint pattern matrix were also log-transformed. One of the main concerns with data not meeting model assumptions is that type II errors (i.e. false negatives) could result, and where model assumptions do not hold, this suggests that an alternative test might do better in detecting significant patterns, though every test has its limitations. This is illustrated below, as we explore the impact of data transformations on our results.*

*As an example of our effective use of data transformations, below we examine genes BCAR4 (top) and IGF2 (bottom), which genes were highlighted in main Figure 2E as examples of significant genes. Gene expression levels corresponding to SVs located in the genomic region 1Mb downstream to 1Mb upstream of the gene are shown, with absolute relative distance (upstream or downstream) represented on the x-axis. Each point represents a single case (closest SV breakpoint represented for each case). On the left, both expression and relative distance are untransformed; in the middle, expression but not relative distance is log-transformed; on the right, both expression and relative distance are log-transformed. The figure below illustrates how using data transformations better defines linear relationships. For each gene, all three plots would indicate some kind of linear relationship, but this is best revealed by the plots on the right, where the corresponding trend lines are steepest. With regards to relative distance, the log-transformation is doing exactly what we want it to do; the data points closest to the gene are expanded out, while data points closer to 1Mb are being brought in; in this way, the furthest data points are not being overly-weighted in the analysis. The data plots in the middle of the figure below are what are represented in Figure 2E and elsewhere in the paper, and this is possibly what the Reviewer thought was what we being inputted into the linear models, but it is the data on the right that is actually being used in the linear modeling.*

*The Reviewer suggests simply aggregating the SV events over all gene regions and then binarizing the count, i.e. if a SV occurs in any of the gene regions up to +/-1Mb than consider it as 1, otherwise as 0. We therefore explore this below. Essentially, this proposed approach is analogous to our “genomic region windows” approach in Figure 1 of the paper, but using a much larger region window than +/-100kb. One anticipated problem with using a +/-1Mb window is that we find that there are much fewer significant genes to be found at windows past 1Mb from the gene (Figure 1B), and we may not expect breakpoints at 1Mb to have the same weight as breakpoints much closer to the gene. The +/-1Mb region was originally selected due to the anticipated influence of enhancers, which could influence a gene at distances around ~0.5Mb. In practice (e.g. as illustrated in the figure above), we tend to see a linear relationship between expression and SV breakpoint distances, with farther distances having less of an effect rather than an equal effect.*

*With the proposed genomic region windows approach using a +/-1Mb window, we find many significant genes after CNA and cancer type correction.*

*As illustrated above, there is pretty good agreement between the +/-1Mb genomic region windows (t-statistic in y-axis) and distance metric (t-statistic in x-axis). However, the distance metric method is able to find many more significant genes or can assign a stronger pattern of significant for other genes, which indicates that the +/-1Mb genomic region windows is based on a hypothetical pattern that would not be a good fit for many of the genes. The figure below shows the number of significant genes based on the +/-1Mb genomic region windows (GRW) method, on distance metric (DM) using unlogged distances, and on distance metric using logged distances. While all three methods do uncover lots of significant genes, it is the method used in our paper that uncovers the most genes, because this method is based off of a pattern that would fit the observations we have made regarding many of the genes in our dataset, as well as uses data transformations that would be more suitable for a linear model and hence would result in fewer false negatives.*

*The Reviewer is correct that a real advantage of using a linear model is that it allows in principle easy integration of covariates, which is a critical aspect of our present study. Nonlinear models would not allow us to specify cancer type as a factor, for example, which would raise a host of concerns including our desire to screen out genes that represent tissue-specific markers rather than SV-altered genes. The use of nonlinear models would present other issues as well. For example, an exponential distribution would assume that the expression would go to infinity as the relative distance approaches zero, which is not reflected in the data. In general, nonparametric regression requires larger sample sizes than regression based on parametric models because the data must supply the model structure as well as the model estimates. Nonparametric regression models can become overfit either by including too many predictors or by using small smoothing parameters. This can make a big difference with special problems, such as small data sets or clumped distributions along predictor variables.*

*While it is true that the linear model represents a theoretical distribution, the model does a pretty good job of identifying significant patterns for many of our genes, given the appropriate data transformations used. The permutation testing in our paper, not based on model assumptions, also shows that the linear model is truly identifying large number of genes with non-random patterns. As part of our earlier study with PCAWG consortium, we did try other types of distributions, including binomial and Poisson, but again these models resulted in widespread obvious false negatives. It is quite possible that the phenomenon that we are examining here could be explored using a variety of methods, similar to there being numerous analytical approaches out there for identifying significantly mutated genes, and we could leave it to future studies to define and explore other methods for integrating SVs with expression and methylation.*

2) SV - expression analysis ignores normal-tissue reference: I think that a key issue that has been largely ignored (not just in this study but in many others), is that performing these correlations between SVs and gene expression across cancers may in fact identify many SVs where the aberrant expression is not really an event that is being selected for, i.e. did the authors check whether tumors not harboring the given SV exhibit higher/lower expression relative to the corresponding normal tissue? For instance, they may find a positive correlation between a SV and gene expression in one cancer-type, but maybe tumors of the same type not harboring the SV still exhibit much higher expression compared to the corresponding normal tissue, in which case the overexpression has nothing really to do with the SV event.

*The scenario that the Reviewer presents in the last sentence of the above would not be allowable by our analytic approach; one reason for this is that cancer type is incorporated as a factor into all the models that were used. Therefore, a marker that is generally high in a given cancer type compared to other cancer types as well as to normal adjacent tissues will not be found significant, unless the SV breakpoints can specifically be associated with altered expression to an extent that would not be explainable by cancer type alone.*

*Tumors not harboring the given SV could exhibit higher or lower expression relative to the corresponding normal tissues, and a positive correlation between SV breakpoint and gene expression would still be of interest. Otherwise, genes like TERT would be excluded, where multiple independent studies have already demonstrated the phenomenon of recurrent genomic rearrangements affecting the chromosomal region proximal to TERT and resulting in its up-regulation, in multiple individual cancer types (TCGA KICH paper Cancer Cell 2014; Valentijn et al. Nature genetics 2015 47, 1411-1414; Nagel, I. et al. Blood 2010 116, 1317-1320; Weischenfeldt, et al. Nature genetics 2017 49, 65-74; Peifer, et al. Nature 2015 526, 700-704; Zhang et al. Cell Reports 2018 24, 515-527). However, TERT is also on average more highly expressed in cancer versus normal adjacent tissues for most cancer types (*[*http://firebrowse.org/viewGene.html?gene=tert*](http://firebrowse.org/viewGene.html?gene=tert)*), but this would not invalidate the importance of the findings of the above studies. Regarding TERT in particular, a number of types of genomic alteration may act upon transcription, including nearby SV breakpoint, TERT amplification, promoter mutations, promoter viral integration, and MYC amplification. In addition, there would be issues, described below, regarding the cell of origin of cancers and how those would relate to samples of normal adjacent tissues.*

*There are some important caveats in interpreting differential expression patterns between cancer and “normal” tissues. The main reason is that cancers of a given type arise from a specific cell of origin, while “normal” samples represent a collection of cells of many different types, usually sampled near the tumor itself. In the gene expression space, cancer versus “normal” truly represents an apples-to-oranges comparison. The normal adjacent samples usually consist of stroma and other types of cells, the vast majority of which would not represent a true normal, i.e. the cancer cell of origin. We often do not know for a particular type of cancer what its cell of origin is, and where we may in fact know the cell or origin it is often difficult to sample in the surgical setting. For example, on the basis of immunohistochemical analyses and global gene expression studies (Prasad et al., 2007; TCGA KICH paper 2014), ChRCC kidney cancer is understood to arise from intercalated cells in the distal convoluted tubule of the nephron, while ccRCC kidney cancer arising from cells of a different type in the proximal convoluted tubule; however, “normal adjacent” kidney tissues as sampled by TCGA and other studies in no way represents the respective cells of origin of the above kidney cancer types. “Normal adjacent” kidney does not even represent the nephron; there would presumably be cells from the nephron mixed in with other cells, resulting in a mixture of cellular profiles. To get normal cells representing the cell of origin of a given kidney cancer, one would have to carefully micro-dissect these from the nephron, which is simply not feasible in the surgical setting of cancer patient care. In addition to the above concerns, many cancer types in our study do not have corresponding non-cancer sample profiles, or at most one or two, and there is no pairing of normal to cancer samples.*

*Given the above, we would not use cancer versus normal comparisons as a primarily filter for declaring significance, though we can provide results from tumor-normal comparisons to the reader, perhaps for use as a secondary filter as warranted. In the new Supplementary Figure S2 (also shown below), we provide addition annotation information for the top set of mRNAs with increased or decreased expression associated with nearby SV breakpoint (by distance metric method, FDR<10%). We focus on the set of cases with SVs associated with altered expressed (>0.4SD from the median for positively correlated genes, <-0.4SD for negatively correlated genes). For samples with these SVs, we compared the normalized expression in the affected case, with the average normalized expression of corresponding normal adjacent samples (where data were available). The gene-level average difference in z-normalized values between SV+ cancer cases and the corresponding normals are represented in the first yellow-blue heat map of the new figure below. The second heat map represents cancer versus normal differences for the remaining cases that do not appear impacted by SVs.*

*We see that, overall, genes for which a positive association with SV was found also had higher expression in the SV+ cases versus normal; genes for which a negative association with SV was found also tended to have lower expression in the SV+ cases versus normal. This would seem in line with the expectations of some, though the above caveats regarding tumor versus normal comparisons certainly apply. As noted above, it is possible for genes like TERT to have non-SV+ cases that would also be higher (or lower) in cancer versus “normal” but the SV+ cases must show even higher (or lower) expression than what would be observed in the non-SV+ cases, as one can see by comparing the “tumor vs normal SV-affected cases” heat map with the “tumor vs normal, other cases” heat map, where the former shows more dramatic contrasts from normal. Genes that simply represent differential expression markers for a given cancer type will not make it into the top significant results, rather the SV breakpoints must specifically be associated with altered expression to an extent that would not be explainable by cancer type alone. The information represented in Supplementary Figure S2 is provided as a separate Excel tab in the revised Supplementary Data Files S4, and so readers would be free to use the information from our cancer SV+ versus normal comparisons in using these data as resource, e.g. to focus on particular genes of interest.*

*Analogous to Figure S2, we also provide a new Supplementary Figure S5 (also shown below), where we provide addition annotation information for the top set of CGIs with increased or decreased methylation associated with nearby SV breakpoint (by distance metric method, FDR<5%). We focus on the set of cases with SVs associated with altered methylation (>0.4SD for positively correlated CGIs, <-0.4SD for negatively correlated CGIs). For samples with these SVs, we compared the methylation beta value in the affected case, with the average beta value of the corresponding normal adjacent samples (where data were available). The CGI-level average difference in beta values between SV+ cancer cases and corresponding normals are represented in the purple-cyan heat map of the new figure below. We see that, overall, CGIs for which a positive association with SV was found also had higher methylation in the SV+ cases versus normal; CGIs for which a negative association with SV was found also tended to have lower methylation in the SV+ cases versus normal. The above caveats regarding tumor versus normal comparisons and cancer cell of origin would apply here as well, but the information represented in Supplementary Figure S5 is also provided as a separate Excel tab in the revised Supplementary Data Files S6, and so readers would be free to use this information in examining the top significant CGIs.*

3) Fig.3D: It was unclear to me why the average DNAm difference for the CGI probes was done by comparing one cancer-type to all other cancer-types as opposed to comparing the cancer to the corresponding normal. Why this matters, is because a significant proportion of DNA methylation changes in cancer is the result of cell proliferation, and so comparing across different cancer-types is going to confound interpretation in relation to how proliferative or less proliferative the given cancer is.

*Figure 3D was intended to explore a specific pattern of interest that we had found (Figure 3C), whereby highly significant overlaps were observed, between the genes with positive correlations between expression and nearby SV breakpoint for a given cancer type and the genes with high overall DNA methylation being associated with that same cancer type. One of the involved genes was TERT, a gene of particular interest to our group, for which several CGI probes were highly methylated in chromophobe renal cell carcinoma (chRCC) and for which associations between SV breakpoints and TERT expression were significant specifically for that cancer type (Figure 3D). Of note, the discovery of SV-mediated deregulation of TERT in solid tumors was first made in the chRCC cancer type by our group (TCGA KICH paper, led by Creighton), though the associations involving DNA methylation had not previously been made. This observation suggests that for some genes, such as TERT, higher levels of DNA methylation for some cancer types would represent a barrier to gene over-expression, which SVs appear to help overcome in many cases of our patient cohort. For Figures 3C and 3D, the differential DNA methylation patterns of interest would not necessarily need to be somatic, but may pertain to the cancer cell of origin. In cancer versus normal comparisons, the impact of cell proliferation is likely to be more pronounced that it would be in our analysis.*

*In light of the Reviewer’s concern, we re-examined the 893 CGI DNA methylation probes from Figure 3D, i.e. the top features showing both high cancer type-specific methylation and significant positive correlation between expression and SV breakpoint for any one of the 20 cancer types surveyed. We computed differential methylation in each cancer type versus other cases (t-statistic using logit-transformed values), using cell proliferation as a covariate (the average of the normalized values for a set of cell cycle genes from Whitfield et al. 2002, Mol Biol Cell). The results are shown below, where overall the CGI probes significant in the original analysis remain significant in the second analysis. The second analysis involves a more complicated model with an additional continuous variable, so the t-statistics in the second analysis may be a bit lower, even if the significance or trend is maintained. We therefore conclude that the relative proliferation rates of samples are unable to explain away the observed differential patterns presented in Figure 3D.*

4) Promoter vs gene-body CGI methylation: One of the biggest problems with this paper is that the authors don't stratify the CGI probes according to whether they are promoter or gene-body 3'UTR related, which makes the interpretation of most of the findings somewhat unclear. Even when they do make a distinction such as in SuppFig.S3C, the figure itself does not make it clear where the CPGs map to in relation to the gene. Or for instance, in Fig.3D, how many of the CGI probes being shown are gene-body related, how many are promoter related?

*In the revision, promoter vs gene-body CGI patterns are examined throughout, e.g. in the new Figures 4B and 4C (shown below), Figure 3D, Figure 6E, new Figure S6C, new Figure S8B, and new Figure S5. The results are quite interesting and are now being highlighted in main figures as well as in the Abstract. Figure 4B shows the fraction of CGI methylation probes that are promoter-associated (by ENCODE addition of Illumina 450K array), for all CGI probes, for CGI probes with positive SV correlation (FDR<5% by distance metric method), and for CGI probes with negative SV correlation. Figure 4C shows the fractions of CGI methylation probes according to gene feature regulatory category (UCSC 450K annotation), including gene body (Between the ATG and stop codon; irrespective of the presence of introns, exons, TSS, or promoters).*

*Figures 4B and 4C reveal very striking patterns, whereby CGI probes with positive SV correlation are highly enriched for promoter-associated probes, and CGI probes with negative SV correlation are highly enriched for gene body-associated probes. These patterns are also evident in our new Supplementary Figures S5 (shown above in our response to Review comment #2), as well as in the new Figure S6C (shown below), which stratifies the CGI probes from Figure 4A according to whether they are promoter or gene-body associated.*

*By design, our analytical approach treats all CGI probes the same and makes no assumptions. There would be no technical reason that we might explain, for example, why the SV breakpoints associated with higher methylation would be promoter-associated. The above finding would represent an important biological finding. Interestingly, while promoter methylation is known to silence genes, gene body methylation is often positively correlated with gene expression (Yang et al. Cancer Cell 2014 26: 577-590), though in our case this would apply to only a fraction of the associated genes (Figure 4D). At any rate, it is important that we put forth these observations. We are happy that the Reviewer suggested that we look into this.*

*We have also revised Fig.3D to denote gene-body related versus promoter related CGI probes (similar to the figure shown in our response to comment #3). It turns out that all of the probes in Figure S4C are gene body associated. TERT-associated CGI probe cg02545192 targets a CpG site known to contain a repressive element in close proximity to the TERT core promotor (Peifer et al. Nature 2015, 526:700-704; Lewis and Tollefsbol Front Genet 2016, 7:83) but the 450K annotation does not indicate this, as it requires domain-specific knowledge regarding TERT.*

5) Fig.5C: Fig.5C is an important panel in this paper, and yet here again the authors choose to do the analysis in a way which is perhaps not the most sensible one: in the barplot, the y-axis shows the average difference in DNAm between the rearranged region vs unaltered region based on normal-adjacent tissue. However, the x-axis refers to SV-CGIs groups that were deemed to be of higher or lower methylation by comparing the tumors with the SV to tumors without the SV, regardless of tissue-type. Would it not be more sensible to compare methylation of SV+ tumors within a given tumor type to the normal-adjacent tissue to define the categories on the x-axis? It would certainly make the data more interpretable, and I think it is also the more meaningful comparison?

*In the revised manuscript, we have carried out additional analyses related to Figure 5C, involving additional comparisons between cancer and normal. Based on normal tissues, Figure 5C examines the average DNA methylation represented by the rearranged region versus the unaltered region, with the average difference in methylation beta values computed for all SV-CGI associations, as well as for the subset of SV-CGI associations involving higher or lower DNA methylation. The revised Supplementary Figure S7D (also shown below) also compares methylation of SV+ tumors within a given tumor type to the normal-adjacent tissue (CGI nearby the gene on the other side of the SV breakpoint), for the above SV-CGI association subsets. The revised Supplementary Figure S7E (also shown below) compares methylation of SV+ tumors within a given tumor type to the normal-adjacent tissue to define the categories on the x-axis; methylation of SV+ tumor was compared with corresponding normal, and all SV-CGI associations for which the cancer-normal beta difference was either greater than 0.4 or <-0.4 were respectively examined for the normal tissue differences represented by rearranged region versus unaltered region.*

*In Figure S7D, we see that, for the subset of SV-CGI associations for which the SV is associated with either higher or lower methylation across cancers, the methylation in the cancer cases also tends to be respectively higher or lower versus the corresponding normal on the other side of the breakpoint. In Figure S7E, we see that the subsets of SV-CGI associations showing higher or lower methylation in the SV+ cases versus normal also respectively show higher or lower average differences in normal methylation as represented by SV breakpoint. The above trends are more pronounced for the SV-CGI associations involving lower methylation, which were the original main focus of Figures 5C and 5D.*

6) Cell-type heterogeneity: the authors do adjust for tumor purity throughout their analysis, but when generating the data for Fig.5C, they also seem to ignore the fact that some of the loci could be cell-type specific markers (say marking immune cells), which could then confound the categorization of the SV-CGI groups on the x-axis i.e. differences in methylation between SV+ and SV- tumors could be due to varying amounts of immune cells.

*In light of the Reviewer’s concern, we examined the correlation between estimated tumor purity and the average methylation beta change metric from Figure 5C (comparing rearranged vs unaltered region, based on normal tissues). We computed the overall correlation for all SV-CGI associations, as well as for the subset of SV-CGI associations involving higher or lower DNA methylation. In none of the above tests, was a significant correlation observed between purity and the average difference in methylation beta values (lowest p-value = 0.14 by Spearman’s). In addition, none of the top genes represented in Figure 5D would seem to represent markers of non-cancer cells.*

7) Pan-cancer analysis vs cancer-specific analysis: It would appear that in the expression analysis at least, that most of the cancer-specific associations are indeed not shared with other cancer-types. In the pan-cancer analysis, I worry that some of the associations which were significant in the pan-cancer analysis are perhaps driven by the larger number of cancer samples for one given cancer-type. So, some of the pan-cancer hits may not really be pan-cancer or only appear so driven by the larger number of samples in one cancer-type: whilst an association in another cancer-type, say if seen for 2 samples of this cancer-type may appear to justify the pan-cancer status, statistically this may be the incorrect interpretation….

*We did find that a large number of genes found significant in analysis of individual cancer types did not reach significance when analyzing the combined pan-cancer set of 2334 cases (Figure 3B), but this would be consistent with findings from genomic surveys for significantly mutated genes (Lawrence et al., Nature 2014, 505:495-501) and significantly altered copy regions (Zack et al. Nature Genetics 2013, 45: 1134-40). In fact, our Figure 3B was modeled after a similar type of figure from the Lawrence et al. paper. So, the observations involving Figure 3B should presumably not be of concern. It is a feature of our dataset that some cancer types would be more represented than others. Also, under-representation would not be limited to numbers of cases, as some cancer types are much more “quiet” than others in terms of SVs or associated CNA; for example, Thyroid, Kidney, and Lymphoma cancers simply have fewer SVs on average than other cancer types. This would be an aspect of most any TCGA pan-cancer or PCAWG study. The way to deal with this is for us to understand that “pan-cancer analysis” does not result in molecular features equally impacted in all cancer types, but rather in molecular features that have arisen from a survey of cases from several different cancer types analyzed together. By the second definition, we would remain interested in molecular features predominantly impacted in a particular cancer type.*

*In the new Supplementary Figures S2 and S5 (shown above under comment #2), we have taken the top set of genes and CGIs associated with altered expression or methylation, respectively, and annotated these molecular features according to impacted cancer types. For each significant feature, the cases involving an SV breakpoint with expression or methylation>0.4SD or <-0.4SD from median are broken down by cancer type. It can be observed from these figures that most cancer types are contributing to the top significant molecular features by pan-cancer analyses. The information represented in Supplementary Figures S2 and S5 is provided as separate Excel tabs in the revised Supplementary Data Files S4 and S6, respectively. Most all of the significant features involved breakpoints associated with altered levels that span multiple cancer types. Out of the 919 significant features in Figure S2, 916 involve breakpoints from more than one cancer type. We do see from Figure S2 that cases from specific cancer types, like breast, ovary, and uterus on average tend to contribute a higher percentage of involved SVs, but this would have to do with more than sample representation, as breast cases for example constitute less than 5% of the total cohort (96 out of 2334) but at a genomics level these breast cancers tend to be very busy.*

8) Confounding effect of cell-proliferation on DNAm: the whole manuscript ignores the effect of cell proliferation on DNA methylation, which is significant and a major reason why the aberrant DNAm landscape across cancer-types is so similar. This, together with the fact that CGI probes were not stratified according to gene region, makes some of the results hard to interpret. For instance, overall SV burden was associated with lower overall DNAm, but treating all CGI probes equally does not allow for this to be correctly interpreted. I would like to see all analyses performed depending on where the CGI probe maps to, so e.g. for Fig.6C we should display this for CGI promoter and CGI gene body probes separately.

*Regarding the analyses involving SV associations for specific CGI-altered methylation (analyses surrounding Figure 4), we have added an analysis evaluating significant associations when correcting for cell proliferation as a covariate. To define a cell proliferation index for each sample, we used the average of the normalized values for a set of cell cycle genes from Whitfield et al. (2002, Mol Biol Cell 13: 1977–2000). Results are shown below as well as in the revised Figure S6A (the model with proliferation as a covariate being the last one in the plot). As with the other possible covariates considered, proliferation was not found to represent a major confounder of the methylation-SV associations reported.*

*Regarding the observation of overall SV burden being associated with lower overall DNA methylation (related to Figures 6 and 7), high overall SV burden definitely correlates with expression of cell cycle genes, as reported. This would be a different phenomenon from that of the more targeted associations involving specific CGI as shown in Figure 4. At the Reviewer’s request, we have carried out the analysis of Figure 6C separately for CGI probes, promoter-associated CGI probes, and gene body-associated probes. (Figure 6C involves overall methylation, including non-CGI probes, where overall methylation for CGI probes is much lower than for the rest of the probes, e.g. as shown in Figure S7C.) Results are shown below as well as in the new Figure S8B.*

*Overall, the inverse trends observed when considering overall DNA methylation patterns (based on all 450K Illumina probes) are similar when considering median overall methylation for just the CGI probes (left) and for the promoter-associated CGI probes (middle). The trends are a bit weaker, and this could be attributed in part to the fact that CGI methylation overall is much lower. The trend between gene body-associated CGI methylation and SV burden may look positive (right) but this was found not to be significant after corrections were made for cancer type (“corrected p”).*

*In addition, for Figure 6E, we have also added “promoted-associated” and “gene body-associated” tracks off to the side of the top different methylation heat map.*

Minor points:

-Adjustment for CNA: When adjusting for CNAs it is not clear how the CN-state of the gene was defined, since the copy-number state refers to a genomic segment, and the correlation analysis is done between expression and distance to BP. So for instance, was a CN- gain including the regulatory element of the gene required for a gene to be assigned the status of 1-copy gain? Approximations/errors like these could easily lead to apparent "independence" in multivariate regression, but only because the CNA covariate is subject to error.

*Gene-by-sample CNA tables, based on either SNP array or WGS, were provided by TCGA Network and PCAWG consortium, respectively. We harmonized the two datasets for our analyses, as illustrated in Figure S1. The CNA is computed within each gene boundary for each sample. By our analysis approach, it would therefore be possible, for example, for a gene not to be amplified but for the region adjacent to the gene containing an enhancer to be amplified (e.g. by tandem duplication SV), thereby increasing gene expression, though this scenario would be of interest to us for inclusion in the final results. Promoter elements being amplified or deleted (e.g. by tandem duplication or deletion SVs, respectively), but with the gene itself not having CNA, would represent SV-mediated disruptions of regulatory regions not explainable by gene-level CNA, which would the primary focus of the present study.*

- Correction for CN change when inferring DNAm: I wonder if the authors considered that measured DNAm levels could also be confounded/affected by the underlying CN-state in which the CGI probes are found. Could the authors please comment? See e.g. Zhou W, Triche TJ Jr, Laird PW, Shen H. Nucleic Acids Res. 2018 Nov 16;46(20):e123.

*As a core aspect of our own study, we explicitly corrected for CNA in each of the CGI feature sets put forth for downstream analyses. The approaches we used would be conceptually similar to the approach used in the NAR paper to identify methylation patterns after correcting for genomic deletions. In our own study, we would not be examining how somatic CNA affects DNA methylation, but rather how SVs affect methylation to a degree that would not be explainable by any associated CNA. While the Zhou et al. study notes that the boundary resolution of using SNP6 arrays to identify CNA regions may be limited by the distribution of probes on the SNP6 array, our own study is unique in that we have both WGS-based copy profiles and SNP-based copy profiles for a large proportion of our samples, where there was very good agreement between the two platforms in terms of gene-level CNA calling (Supplementary Figure S1).*

*At the same time, it might be theoretically possible for a CGI probe itself to be amplified, for example, but the nearby gene to have no CNA. Such phenomenon would be of potential interest, as it would suggest precise targeting of an element near the gene, and might be explored on a case-to-case basis. However, we don’t believe that the above scenario would be at all common for the following reasons: 1) most CGI probes are very close to the gene boundary (e.g. within 200 bases); 2) CNA regions in human cancers are typically broad and involve many genes; 3) only a minority of the SVs associated with CGI methylation increases involve tandem duplications (new Supplementary Figure S6 and response to Reviewer #1, point 1); 4) most all significant CGI probes associated with decreased methylation in particular are highly enriched for CGI probes within the gene body (response to Reviewer #2, point 4). Notably, of the 1286 significant CGI probes (FDR<5% by distance metric), 954 probes fall within the gene boundary as defined by Ensembl, which boundaries would have been used in calling gene-level CNA, and the remaining CGI probes had a median distance of just 143 bases from the gene start. In contrast, many of the methylation probes considered in the Zhou study would be in non-CGI regions far away from genes.*

**Second round of review**

**Reviewer 1**

I have carefully read the revised manuscript and the author’s responses to the earlier critiques. I think the manuscript is much improved in all aspects, and especially regarding how it addresses the core concern I had, that the analysis was not sufficiently taking into account copy number changes associated with many of the SVs. The authors explain, both in the manuscript and in their responses, how correction for CNAs is carried out, which is, I think, a key step in the analysis. 

All my other comments were also addressed, about how the SVs were called, their size distribution, etc. Here a minor point about nomenclature that just occurred to me in this context: the authors have a point in using CNA as acronym for the copy number changes, not CNV, I think the term CNV indeed seems to be more common for variants in the germline, while the A in CNA reminds one of aberration (although it is meant to spell out alteration if I understand correctly), which is appropriate for cancer. But then why use SV and not SA, or SGA (structural genome alteration), or something along that line? But this is just some musing, no need to make any changes if SV is what the field uses.

Two more minor points: 
The manuscript describes gene expression signatures for some of the cancers that indicate immune cell infiltration. It could be helpful to specify the types of tumors in which this is happening, maybe also in the form of a table in the Supplement – and then also add literature that shows that in these types of tumors this kind of immune infiltration is indeed a known phenomenon. One would expect that there are studies on the histopathology of the tumor types in question that show for example with microscopy the presence of infiltrates?

And about the SVs disrupting TADs, I understand that an SV that spans two TADs has a large potential to be disruptive of these domains, as it will delete (for example) the boundary regions that are important factors in forming the TADs in the first place. But couldn’t a large deletion that takes out, for example, 50% or even 80% of the sequence under a give TAD be disruptive? The results of the analyses on this in this manuscript show that the former are more disruptive in general than the latter, and that makes sense. But with these analyses done, could one take just the part that deals with SVs entirely within TADs a check if there is a gradient of disruptiveness based on the size of the SV? This is not essential to be done, I am just curious.

**Authors Response**

**Point-by-point responses to the reviewers’ comments:**

Reviewer #1 comments: 

I have carefully read the revised manuscript and the author’s responses to the earlier critiques. I think the manuscript is much improved in all aspects, and especially regarding how it addresses the core concern I had, that the analysis was not sufficiently taking into account copy number changes associated with many of the SVs. The authors explain, both in the manuscript and in their responses, how correction for CNAs is carried out, which is, I think, a key step in the analysis.

*WE DEEPLY THANK THE REVIEWER FOR THE INVESTMENT OF TIME IN HELPING TO IMPROVE OUR MANUSCRIPT.* 

1) All my other comments were also addressed, about how the SVs were called, their size distribution, etc. Here a minor point about nomenclature that just occurred to me in this context: the authors have a point in using CNA as acronym for the copy number changes, not CNV, I think the term CNV indeed seems to be more common for variants in the germline, while the A in CNA reminds one of aberration (although it is meant to spell out alteration if I understand correctly), which is appropriate for cancer. But then why use SV and not SA, or SGA (structural genome alteration), or something along that line? But this is just some musing, no need to make any changes if SV is what the field uses.

*THE REVIEWER MAKES A GOOD POINT. IN ORDER TO ENSURE THAT THE READER UNDERSTANDS THAT SOMATIC, NOT GERMLINE STRUCTURAL VARIANTS ARE THE FOCUS OF THE STUDY, WE HAVE REPLACED THE PREVIOUS "SV" ABBREVIATION (FOR STRUCTURAL VARIANT) WITH "SSV," FOR *SOMATIC* STRUCTURAL VARIANT, AS FIRST DEFINED. ALL PREVIOUS INSTANCES OF "SV" IN THE MANUSCRIPT AND FIGURES HAVE BEEN EDITED ACCORDINGLY IN THE REVISION.* 

Two more minor points: 
2) The manuscript describes gene expression signatures for some of the cancers that indicate immune cell infiltration. It could be helpful to specify the types of tumors in which this is happening, maybe also in the form of a table in the Supplement – and then also add literature that shows that in these types of tumors this kind of immune infiltration is indeed a known phenomenon. One would expect that there are studies on the histopathology of the tumor types in question that show for example with microscopy the presence of infiltrates?

*IN THE REVISION, WE HAVE INCLUDED A REFERENCE TO OUR PREVIOUS PAN-CANCER PAPER IN THE CONTEXT OF THE BINDEA IMMUNE SIGNATURE RESULTS. IN THIS PREVIOUS PAPER (PMID: 29440175), WE SCORE ALL THE TCGA CANCER FOR THE BINDEA IMMUNE SIGNATURE, WHERE TWO MAJOR GROUPS OF TUMORS (C3 AND C10) SHOW PARTICULARLY HIGH IMMUNE INFILTRATE. PIE CHARTS SHOWN IN FIGURE 3D OF THIS PREVIOUS PAPER (AS WELL AS ASSOCIATED SUPPLEMENTARY TABLES) PROVIDE BREAKDOWNS BY CANCER TYPE.* 

3) And about the SVs disrupting TADs, I understand that an SV that spans two TADs has a large potential to be disruptive of these domains, as it will delete (for example) the boundary regions that are important factors in forming the TADs in the first place. But couldn’t a large deletion that takes out, for example, 50% or even 80% of the sequence under a give TAD be disruptive? The results of the analyses on this in this manuscript show that the former are more disruptive in general than the latter, and that makes sense. But with these analyses done, could one take just the part that deals with SVs entirely within TADs a check if there is a gradient of disruptiveness based on the size of the SV? This is not essential to be done, I am just curious.

*THE SCENARIO THE REVIEWER PUTS FORTH IS ENTIRELY PLAUSIBLE AND WOULD BE INCLUDED IN OUR TOP RESULTS. WHILE WE DO SEE AN ASSOCIATION OF TAD DISRUPTION WITH ALTERED EXPRESSION OR METHYLATION (FIG 5A), THIS STATISTICALLY SIGNIFICANT ASSOCIATION DOES NOT IMPLY THAT *ALL* SSV-ASSOCIATED DISRUPTIONS WOULD SPAN TAD BOUNDARIES. IN FACT A SIZABLE PERCENTAGE OF SSV EVENTS ASSOCIATED WITH ALTERED GENE REGULATION (~25-40%) DO NOT INVOLVE TAD DISRUPTION, AND A SIZABLE PERCENTAGE OF THESE WOULD INVOLVE LARGE SSVS, AS INDICATED IN FIG S3E AND S6E.*
